# Genome-wide association study of agronomic traits in bread wheat reveals novel putative alleles for future breeding programs

**DOI:** 10.1186/s12870-019-2165-4

**Published:** 2019-12-05

**Authors:** Yousef Rahimi, Mohammad Reza Bihamta, Alireza Taleei, Hadi Alipour, Pär K. Ingvarsson

**Affiliations:** 1 Department of Agronomy and Plant Breeding, Faculty of Agriculture, University of Tehran, Karaj, Iran; 2 Department of Plant Breeding and Biotechnology, Faculty of Agriculture, Urmia University, Urmia, Iran; 3 Linnean Centre for Plant Biology, Department of Plant Biology, Swedish University of Agricultural Sciences, Uppsala, Sweden

**Keywords:** Wheat, Agronomic traits, GWAS, MTAs, Gene annotation

## Abstract

**Background:**

Identification of loci for agronomic traits and characterization of their genetic architecture are crucial in marker-assisted selection (MAS). Genome-wide association studies (GWAS) have increasingly been used as potent tools in identifying marker-trait associations (MTAs). The introduction of new adaptive alleles in the diverse genetic backgrounds may help to improve grain yield of old or newly developed varieties of wheat to balance supply and demand throughout the world. Landraces collected from different climate zones can be an invaluable resource for such adaptive alleles.

**Results:**

GWAS was performed using a collection of 298 Iranian bread wheat varieties and landraces to explore the genetic basis of agronomic traits during 2016–2018 cropping seasons under normal (well-watered) and stressed (rain-fed) conditions. A high-quality genotyping by sequencing (GBS) dataset was obtained using either all original single nucleotide polymorphism (SNP, 10938 SNPs) or with additional imputation (46,862 SNPs) based on W7984 reference genome. The results confirm that the B genome carries the highest number of significant marker pairs in both varieties (49,880, 27.37%) and landraces (55,086, 28.99%). The strongest linkage disequilibrium (LD) between pairs of markers was observed on chromosome 2D (0.296). LD decay was lower in the D genome, compared to the A and B genomes. Association mapping under two tested environments yielded a total of 313 and 394 significant (−log_10_ *P* >3) MTAs for the original and imputed SNP data sets, respectively. Gene ontology results showed that 27 and 27.5% of MTAs of SNPs in the original set were located in protein-coding regions for well-watered and rain-fed conditions, respectively. While, for the imputed data set 22.6 and 16.6% of MTAs represented in protein-coding genes for the well-watered and rain-fed conditions, respectively.

**Conclusions:**

Our finding suggests that Iranian bread wheat landraces harbor valuable alleles that are adaptive under drought stress conditions. MTAs located within coding genes can be utilized in genome-based breeding of new wheat varieties. Although imputation of missing data increased the number of MTAs, the fraction of these MTAs located in coding genes were decreased across the different sub-genomes.

## Background

Bread wheat (*Triticum aestivum* L.) is a staple crop both in developing and developed countries and there is a constant need to balance supply and demand [1]. The projected increase of human population is expected to increase the demand of wheat thereby highlighting the need for plant breeders to utilize all accessible tools to find new ways to sustainably increase the production of bread wheat over the coming decades [1, 2]. Wheat production increased significantly after green revolution in the 1960s and 1970s by better farm management practices and introduction of well-adapted wheat varieties. However, the global consumption of wheat has also steadily been increasing due to population boom [1]. The comparison between wheat production and consumption in 1962 and 2012 shows a substantial increase in demand for wheat, with China, EU, India, and the USA being the major consumers. However, looking at the increase in demand over the 50-year period, it becomes clear that Asian countries, including Indonesia, Bangladesh, and Thailand are at the top of the list [1]. There are several factors drastically limiting wheat production throughout the world, such as water deficiency, salt and cold stress, resulting in significant losses in both grain and biomass [3, 4].

Understanding drought tolerance mechanisms and identifying loci responsible for mediating drought tolerance are key steps for any breeding approach aimed at increasing stress tolerance induced by water deficiency in bread wheat. Recent progress in sequencing technologies have become genome databases available for model plants, such as *Arabidopsis thaliana* [5], *Oryza sativa* [6], and a number of important crop species, including *Hordeum vulgare* [7] and *T. aestivum* [8]. The availability of large-scale genomic resources provides an opportunity to discover genetic and molecular mechanisms behind plant responses to different environmental stresses. As most agronomically important traits are likely controlled by a large number of genes, quantitative trait loci (QTL) mapping has been widely used to dissect the genetic architecture of such traits [9–11]. However, QTL-mapping has several drawbacks, such as low resolution and a limited number of alleles that can be screened per study. The high-throughput genotyping technologies providing large number of single nucleotide polymorphism (SNP) data has drastically improved the resolution of QTL mapping by providing high-resolution linkage maps [12].

Furthermore, due to the increased availability of large-scale genomic resources, genome-wide association studies (GWAS) are now a viable alternative to QTL mapping for dissecting the genetic architecture of quantitative traits [13]. In comparison to QTL-mapping, GWAS help accelerates the assessment of a more representative set of individuals in both time and cost-effective way [14]. GWAS are based on establishing correlations between genotype and phenotype, with the idea that linkage disequilibrium (LD) has been formed in a population across generations so that regions harboring QTLs can be detected even if the causal mutations aren’t necessarily included among the set of available genetic markers. Access to high-density genotyping spanning the entire genome makes GWAS invaluable tools for identifying genomic regions underlying the observed phenotypes. Several recent studies have successfully applied GWAS to identify the genetic basis of important traits in a number of crop species, including rice [15], barley [16], corn [17] and wheat [18]. These studies have also provided information about MTAs, which can help breeders in marker-assisted selection. In particular, a number of recent studies have focused on wheat by identifying QTLs associated with grain yield and related traits [19–21].

Genotyping by sequencing (GBS) can provide access to a large number of SNPs in a cost-efficient manner but it is often plagued by a high fraction of missing data that can limit the accuracy of any genome-wide association study. One approach to deal with missing data is through imputation and this has successfully been implemented in many studies on human and plant genomes [22–26]. Imputation can increase the number of variants that are included in a GWAS by relying on linkage information derived from common haplotypes after considering SNPs which are not directly genotyped [27]. Low depth sequencing and library complexity may contribute to missing information in SNP data and genotype imputation can thus be utilized to partly compensate for such issues through available reference genomes without the need for additional expensive resequencing [28, 29]. The main objective of the current study was to perform a GWAS experiment using GBS-SNP data [30] and SNPs imputed based on the W7984 reference genome for bread wheat, which has previously been demonstrated to yield the highest imputation accuracy [31]. A set of three categories of agronomic traits were measured among Iranian wheat landraces and varieties and employed in an association study to explore putative QTLs to identify genes, which may be involved in important developmental pathways providing drought tolerance. The second objective of the study was to determine if there are any differences in the results produced using the original SNP data compared to the imputed SNP data by assessing the influence of imputation on MTAs.

## Results

### Phenotypic evaluation

The datasets for well-watered and rain-fed conditions were analyzed separately. ANOVA identified significant differences (*P< 0.01*) among varieties and landraces for all studied traits under both environments for two years, except thousand kernel weight under rain-fed conditions (Additional file 2: Tables S2 and 3). Under rain-fed conditions, early emergence was delayed, yet genotypes completed their lifespan earlier compared to the well-watered conditions through a 14.9 days reduction in physiological maturity (Table 1). The grain filling period was 27.2 and 24.0 days for well-watered and rain-fed conditions, respectively. The greatest variation under well-watered conditions was observed for seed number per spike and thousand kernel weight (std. deviation 7.35 and 7.10, respectively), whereas plant height and peduncle length were more variable under rain-fed conditions (SD 15.55 and 7.26, respectively). A significant positive association was observed between grain yield, spike weight, seed number, thousand kernel weight, leaf greenness, and grain filling period under well-watered conditions (*P < 0.01*), whereas phenology traits and canopy temperature were negatively correlated with grain yield (*P < 0.01* and *0.05*, Additional file 2: Table S4). Under rain-fed conditions, grain yield was negatively correlated with phenological traits, plant height, peduncle length, spike length, and canopy temperature, whereas significant positive correlations were observed between grain yield, spike weight, seed number per spike, and thousand kernel weight (*P < 0.*01, Additional file 2: Table S5).

**Table 1:** Descriptive statistics for agronomic traits of Iranian wheat accessions under well-watered and rain-fed conditions

	well-watered				Rain-fed	
Trait	Range	Mean	Std. Deviation	Range	Mean	Std. Deviation
DE	19.9–27.5	24.7	1.6	22.9–37.8	28.5	2.4
DH	160.0–188	175.9	9	135.4–179.8	167.1	6.6
DA	167.6–196.6	184.9	5.9	158.7–185.9	173	6.1
DM	194.6–224.7	211.9	6.2	183.2–209.4	197	6.4
GFP	20.5–35.5	27.2	2.5	16.7–31.5	24	2.8
H	–	–	–	53–130	87.1	15.6
PL	–	–	–	19.9–64.2	36.3	7.3
SW	1.1–4	2.5	0.44	0.90–2.6	1.7	0.35
SL	5.7–18.7	10.8	2.1	6.7–13.2	9.9	1.1
GY	0.66–2.8	1.8	0.35	0.47–1.8	1.1	0.24
SN	23.8–72.3	43.4	7.4	23.5–57.9	37.7	7
TKW	14.9–74.5	41.0	7.1	15.6–44.7	29.6	5.7
LG	37.94–66.1	51.3	4.9	33.7–62.5	49.1	5.1
CT	20.1–27.8	23.9	1.4	22.1–33.1	27.6	1.6

DE: Days to emergence, DH: Days to heading, DA: Days to anthesis, DM: Days to physiological maturity, GF: Grain filling period, H: Plant height (cm), PL: Peduncle length (cm), SW: Spike weight (g), SL: Spike length (cm), GY: Grain yield (g per plant), SN: Seed number per spike, TKW: Thousand kernel weight (g), LG: Leaf greenness, CT: Canopy temperature (°C)

### Evaluation of SNP markers

**Fig. 1:**
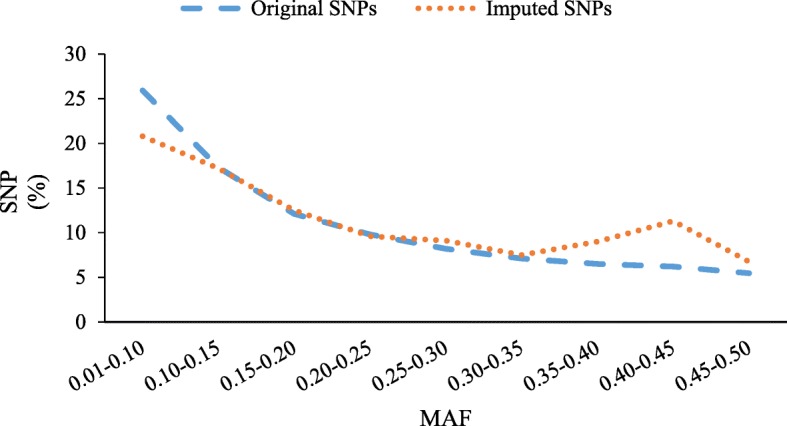
The distribution of SNPs according to different MAF for the original and imputed datasets

A total of 458,363,607 unique reads were identified in total 566,439,207 reads after sequencing (~ 81% non-redundant reads). After de-duplication and alignment, 133,039 SNPs were called for which 10,938 had < 10% missing data, heterozygosity < 10% and a minor allele frequency (MAF) >1%. These SNPs were selected for further analysis. Among the 10,938 SNPs identified, the highest (2835, 25.92%) and lowest (597, 5.46%) number of markers were observed for MAFs in the range of 0.01–0.1, and 0.45–0.50, respectively (Fig. 1). In addition, we obtained a set of 46,862 imputed SNPs using the W7984 reference genome and these SNPs were also used to estimate genetic diversity.

### Linkage disequilibrium (LD)

The analysis of linkage disequilibrium shows that LD differs between sub-genomes, chromosomes, and across each chromosome and that LD generally declines with increasing distance between SNPs. A total of 368,310 marker pairs (MP, according to combinations of SNPs across different chromosomes) with average squared allele frequency correlations or *r*^*2*^ = 0.132 were observed in varieties, of which 96,541 (26.2%) had significant linkage at *P< 0.01* (Table 2). Around 93% of all MPs and 94% of significant MPs were located at distances <10 cM. Genomes B and D harbored the highest and lowest number of MPs (182,271, 49.49% and 50,395, 13.68%), respectively. Moreover, the strongest LD was observed between MPs on chromosome 2D (0.296), followed by chromosome 1D (0.214).

**Table 2:** A summary of observed LD among marker pairs and the number of significant marker pairs per chromosome and genome using original SNPs

Chromosome	varieties				Landraces			
	TNMP	*r*^*2*^	Distance (cM)	SMP	TNMP	*r*^*2*^	Distance (cM)	SMP
1A	17,718	0.1106	5.9776	4338 (24.48)	22,647	0.0813	5.1594	5835 (25.77)
1B	24,887	0.1465	3.8458	6559 (26.36)	28,687	0.0963	3.8538	9506 (33.14)
1D	10,568	0.2104	9.3147	3773 (35.70)	13,133	0.1195	10.5104	4337 (33.02)
2A	21,453	0.1341	4.2847	5487 (25.58)	25,306	0.1154	3.9947	8527 (33.70)
2B	36,066	0.1327	3.3614	9728 (26.97)	33,314	0.0925	3.4718	10,666 (32.02)
2D	12,523	0.2959	6.0466	4741 (37.86)	16,319	0.1976	6.1044	5473 (33.54)
3A	21,696	0.1159	7.5667	4831 (22.27)	19,424	0.0748	7.4690	4629 (23.83)
3B	31,120	0.1327	3.8233	8632 (27.74)	33,719	0.0974	3.8089	10,860 (32.21)
3D	4274	0.1117	14.9170	713 (16.68)	7601	0.0994	17.2124	1782 (23.44)
4A	16,982	0.1484	6.6126	4548 (26.78)	17,092	0.1164	7.0726	5002 (29.27)
4B	11,382	0.1679	6.8900	3505 (30.79)	8498	0.0608	8.6884	1554 (18.29)
4D	1918	0.1836	22.8230	492 (25.65)	2329	0.1422	22.8137	1037 (44.53)
5A	15,226	0.1217	6.3518	3614 (23.74)	17,683	0.0867	6.8862	5281 (29.86)
5B	28,463	0.1427	5.4429	8533 (29.98)	29,599	0.0728	5.4563	7454 (25.18)
5D	5524	0.1049	23.4950	848 (15.35)	6152	0.0742	27.2205	1339 (21.77)
6A	16,916	0.1120	6.4506	3578 (21.15)	18,115	0.1161	6.4866	6739 (37.20)
6B	23,696	0.1456	3.5509	7080 (29.88)	28,304	0.0729	3.9161	7225 (25.53)
6D	6899	0.1150	16.5648	1375 (19.93)	8454	0.0828	16.0911	2112 (24.98)
7A	25,653	0.1506	4.8667	6132 (23.90)	30,419	0.1052	4.7313	8988 (29.55)
7B	26,657	0.1136	4.1457	5843 (21.92)	27,880	0.0807	3.9193	7821 (28.05)
7D	8689	0.1822	16.5774	2191 (25.22)	11,063	0.0996	15.7344	2766 (25.00)
A genome	135,644	0.1289	5.9344	32,528 (23.98)	150,686	0.0994	5.9714	45,001 (29.86)
B genome	182,271	0.1372	4.1911	49,880 (27.37)	190,001	0.0819	4.7307	55,086 (28.99)
D genome	50,395	0.1928	13.2910	14,133 (28.04)	65,051	0.1165	16.5267	18,846 (28.97)
Total	368,310	0.1321	6.0783	96,541 (26.21)	405,738	0.0968	6.3498	118,933 (29.31)

TNMP: Total no. of marker pairs, SMP: Significant marker pairs (*P<0.01*)

Performing a similar analysis on landraces identified a total of 405,738 MPs with an average LD of 0.097 which is considerably lower than in varieties (Table 2). However, a greater fraction of significant MPs (29.31%) was observed in the landrace data. Eighty-nine percent and 88% of the total and significant MPs had distances < 10 cM. Moreover, the greatest number of MPs was observed in the B genome (190,001). The overall number of SNPs located in different sub-genomes in landraces was slightly higher than in varieties. Similarly, LD was highest in chromosome 2D (0.198). The LD decay is visualized in Additional file 3: Figs. S2–4. LD on chromosomes of the D genome shows a distinct trend, where LD decay occurred more slowly compared to either A or B genomes. For most chromosomes of the A and B genomes, LD declined to 0.1 over distances of < 5 cM, whereas the corresponding distances were 5–10 cM in D-genome.

### Population structure and kinship matrix

**Fig. 2:**
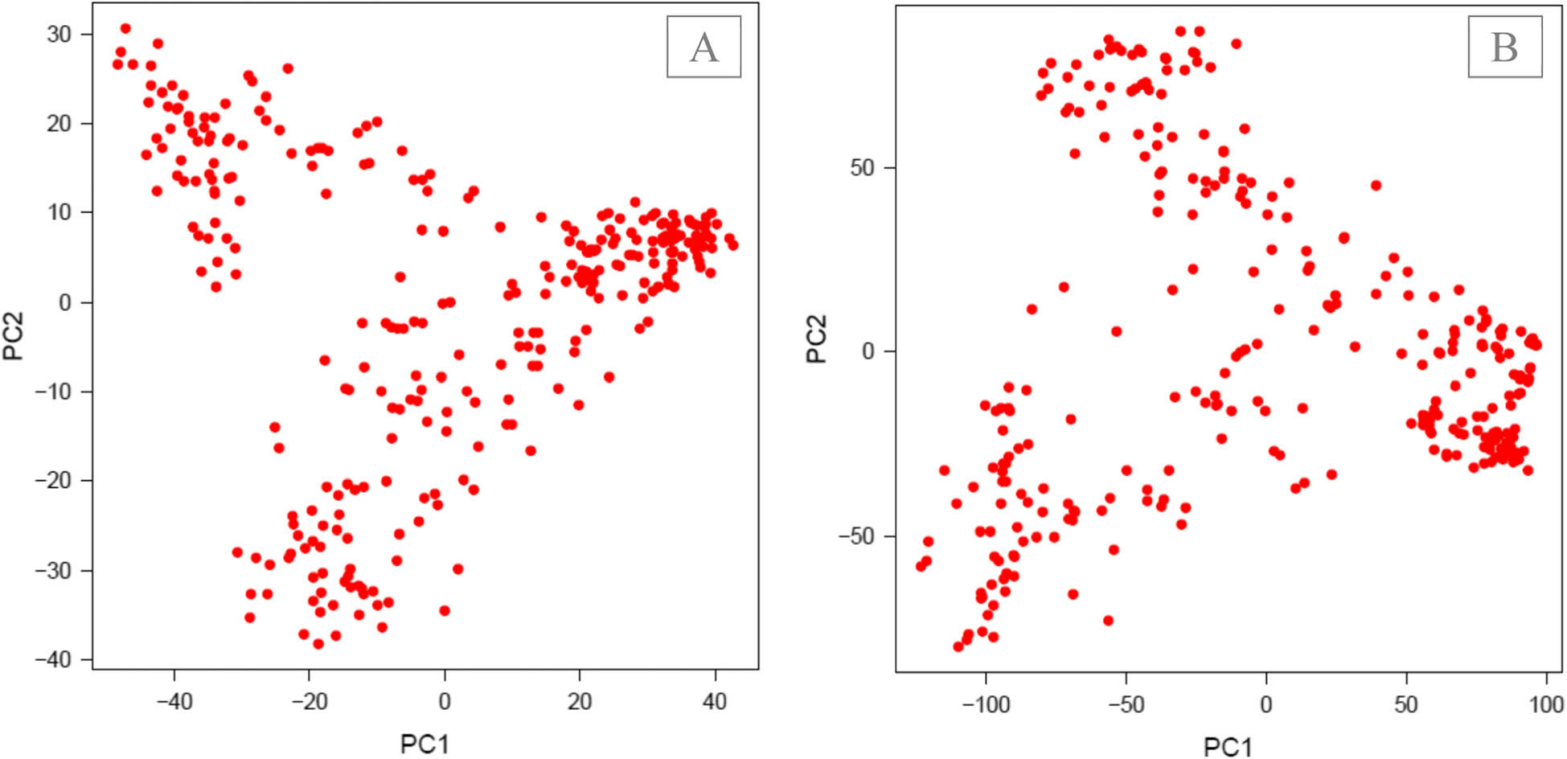
Principal component analysis of Iranian accessions using original SNPs (A), and imputed SNPs (B)

We evaluated population structure using the variance-covariance matrix of individuals (Kinship matrix) obtained from both original and imputed SNPs. For both datasets, the analyses identified three main groups with varying degrees of admixture. For the original dataset, the first two principal components explained 17.2% of the genetic variance (Fig. 2a), whereas the variance was 23.2% for the imputed SNPs (Fig. 2b). Moreover, analysis of population structure showed the highest value of ∆K for K = 3.

**Fig. 3:**
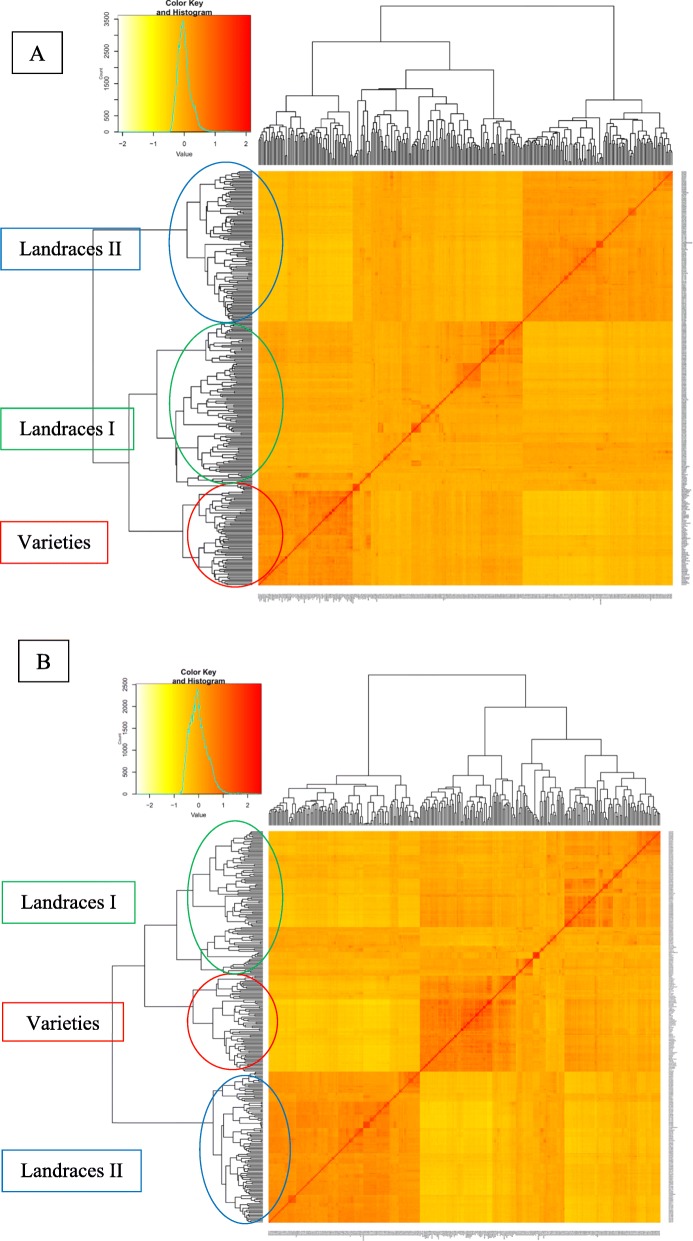
Cluster analysis using Kinship matrix of original data (A) and imputed data (B) for Iranian wheat accessions

Group I contains 69 accessions with 66 varieties and 3 landraces; Group II contains 120 accessions with 102 landraces and 18 varieties, and Group III contains 103 landraces and 6 varieties (Fig. 3a). Accessions also clustered into three main groups when we used the imputed SNPs, where Group I contains 113 accessions with 108 landraces and 5 varieties, Group II contains 74 studies with 70 varieties and 4 landraces; Group III contains 110 accessions with 97 landraces and 13 varieties (Fig. 3b). According to the original SNP data, twenty-four varieties appear to be admixed with the two landrace groups, while for the imputed SNP data, only 19 such admixed varieties were identified. The admixed varieties originated from Iranian landraces and varieties including Shahi, 4820, Mahdavi, Azadi, Ghods, Neishabour and Sivand derived from other materials.

**Fig. 4:**
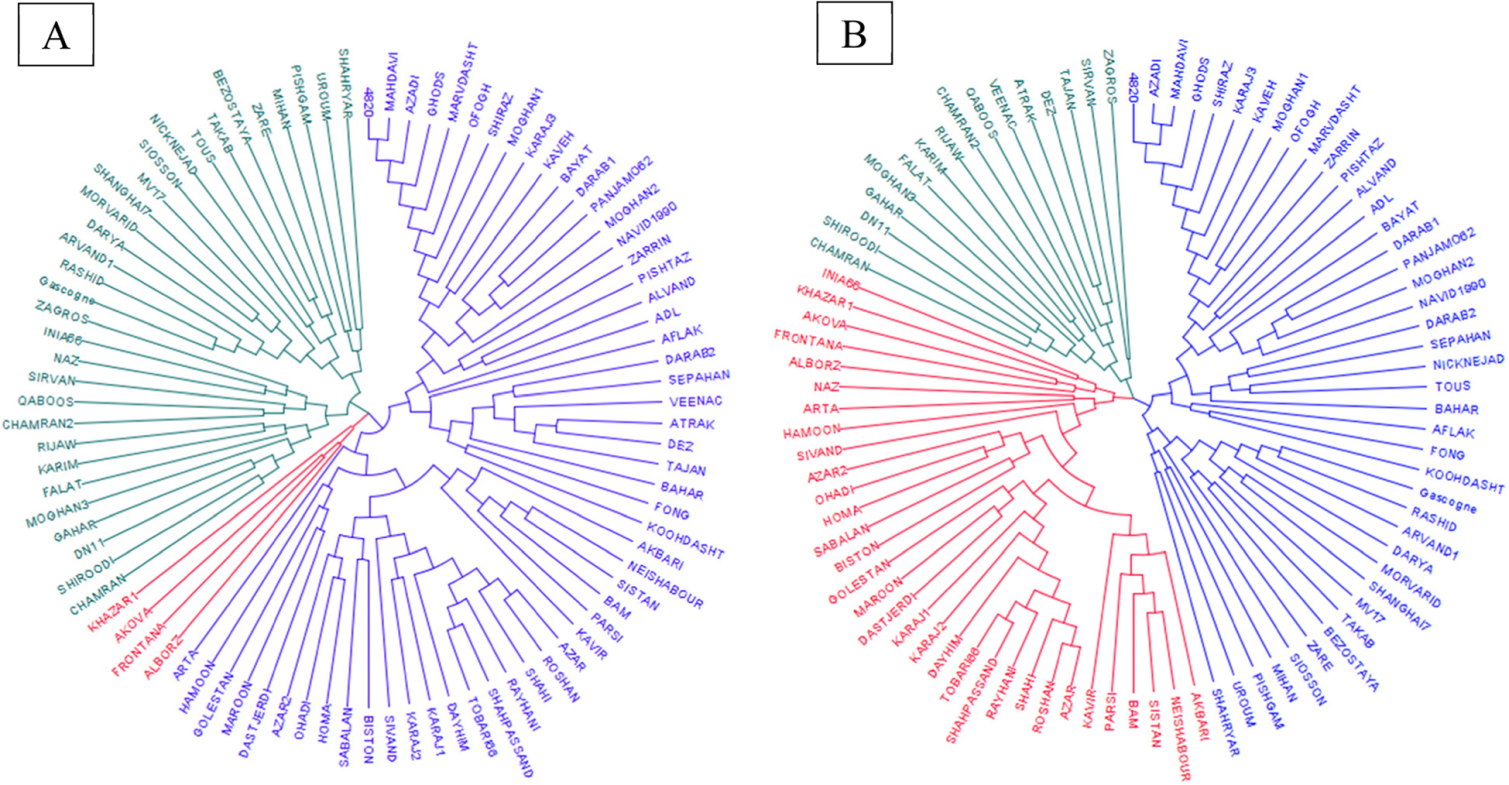
The dendrogram of Neighbor-Joining clustering constructed using 10,938 (A) and 46,862 (B) SNPs and 90 Iranian hexaploid wheat varieties

**Fig. 5:**
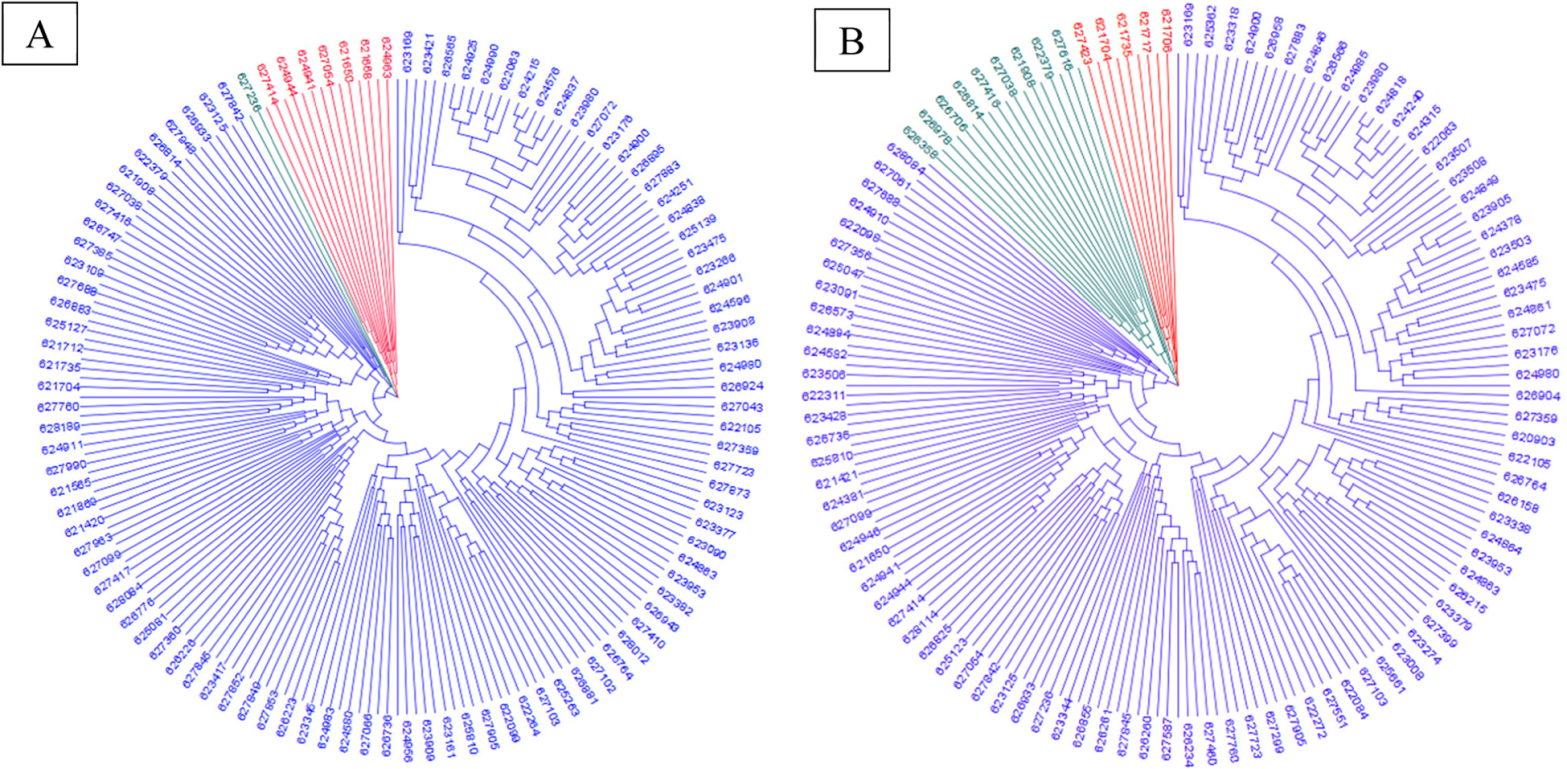
The dendrogram of Neighbor-Joining clustering constructed using 10,938 (A) and 46,862 (B) SNPs and 208 Iranian hexaploid wheat landraces collected from different zones

A neighbor-joining tree of all varieties also clearly showed the clustering into three subgroups for both datasets (Fig. 4), with the exception of the varieties Khazar1, Akova, Frontana, and Alborz which shifted into two series of 12 and 25 varieties of two neighbor’s groups (Fig. 4b). Even though landraces clustered into three groups based on the two SNPs datasets, their differentiation was more clearly distinguished using imputed SNPs (Fig. 5). Accessions PI627236 and PI625433 were grouped into the same group using original SNPs, while accession PI625433 shifted into the largest group of landraces and the distribution of the two small groups changed when the imputed SNPs were used for clustering.

### MTAs for agronomic and physiological traits

A total of 313 MTAs were identified using the original SNP dataset at a significance value of –log_10_ *P* >3 for both well-watered and rain-fed conditions. A total of 394 MTAs were detected for the imputed data (Table 3). The highest number of MTAs was located on chromosomes from the B genome for both the original and imputed SNPs whereas D genome showed the smallest number of MTAs. Among the traits that were studied under well-watered conditions, grain filling period and spike length showed the highest number of associated MTAs for both original and imputed SNPs, respectively. Moreover, 13 and 4 MTAs were observed for grain yield per plant under well-watered conditions using original and imputed datasets, respectively. For drought stress conditions, 23 MTAs were observed for grain filling period and peduncle length based on non-imputed SNPs, whereas 10 and 18 MTAs showed significant association with these traits using imputed SNPs. However, the highest number of imputed MTAs was obtained for seed number per spike (25 MTAs).

**Table 3:** A summary of marker-trait associations for agronomic traits of Iranian wheat accessions under well-watered and rain-fed conditions

Genome	Well-watered		Rain-fed	
	Original data	Imputed data	Original data	Imputed data
MTA	117	177	196	217
Genome A	36	89	60	85
Genome B	51	83	92	110
Genome D	12	5	28	22
Unassembled Chromosomes	18	–	16	–

For the original SNP data, the highest number of significant markers under well-watered conditions was observed on chromosomes 2B and 7B with 12 and 11 SNPs respectively, followed by chromosomes 2A, 3A, and 6B with 8 markers per chromosome. Under rain-fed conditions, a total of 29 and 15 associated SNPs were identified on chromosome 5B and 6B. For the imputed dataset, 47, 32, and 26 significant markers were identified on chromosomes 4B, 1A, and 5B under well-watered conditions, whereas 85, 40, and 28 markers were identified on chromosomes 5B, 4A, and 1A while using the imputed-SNPs under rain-fed conditions. A number of markers on chromosomes A and B showed pleiotropic effects among different traits.

### Gene annotation

The gene ontology of the 313 MTAs that we identified using the original SNP dataset shows that 27 and 27.5% of the MTAs were located within protein-coding genes under well-watered and rain-fed conditions, respectively (Additional file 4: Tables S6, and 7). In contrast, among 394 MTAs identified using the imputed SNP data set, 22.6 and 16.6% were located within coding genes under well-watered and rain-fed conditions, respectively (Additional file 4: Tables S8, and 9). The genes with MTAs mostly encode proteins involved in ubiquitination, oxidation-reduction, protein phosphorylation, histone deubiquitination, negative regulation of transcription, response to abscisic acid, catabolic process, multicellular organism development, xanthophyll biosynthetic, response to UV, ion transportation, cytokinin biosynthetic, DNA methylation, DNA replication, cellular response to DNA damage stimulus, response to oxidative stress, cellular protein modification process, and carbohydrate metabolic process.

We have summarized the results of the SNPs showing the strongest association in Tables 4 to 7. Under well-watered conditions, 5 and 7 markers within coding genes were located on chromosomes 1A, 4B, 4D, 5A, 5B, 6B and 7B for original and imputed SNPs, respectively (Tables 4 and 5). These markers were associated with canopy temperature, seed number per spike, thousand kernel weight, grain filling period, grain yield, spike weight and length, leaf greenness and days to emergence. Most of these SNPs are located on the B genome, followed by the A genome and finally the D genome (Tables 4 and 5). Under rain-fed conditions, 14 and 11 SNPs were associated with genes involved in regulating spike weight and length, thousand kernel weight, plant height, peduncle length, leaf greenness, grain filling period, seed number, grain yield, canopy temperature, days to emergence, heading and physiological maturity (Tables 6 and 7). All these markers were located on the B and A genomes and there was no significantly associated marker located on the D genome under rain-fed conditions. Overall, the B genome contains a considerable portion of all highly significant SNPs for agronomic traits. Markers rs36032 on chromosome 1A and rs56337 on chromosome 7A were linked to genes that are involved in providing grain yield under well-watered and rain-fed environments, respectively.

**Table 4:** Description of expected MTAs using original SNPs for agronomic traits of Iranian wheat accessions exposed to the well-watered conditions

Row	Marker	sequence	Trait	Chromosome	Position (bp)	Molecular process	Biological process
1	rs62576	TGCAGTTACGGATGGCAGTCATCTGGTCCATGAATCATGACAGAGGCACCTGCTCCATAAACAG_47	Canopy temperature	1A	570,131,664-570,140,605	oxidoreductase activity,	oxidation-reduction process
2	rs48893	TGCAGGCTCCGCTAAACCCTAGACTTGACGGCGAGGGTGCGTCGGGTGGGGAAAGGGGGAGAAA_11	Seed number	4D	318,493,437-318,496,592	GTPase activity	–
3	rs4772	TGCAGACTCACACACAAGCTGCTACAACTAAGCGCTGGGCAGATACATCCACCCGAGATCGGAA_44	Thousand kernel weight	5B	435,156,029-435,159,077	protein kinase activity ATP binding	protein phosphorylation
4	rs46504	TGCAGGCATATGCTCGCCCCACATGTTCGTAGACAGGCTATCCTGCCGTTACGCATTGTGGTAC_30	Grain filling period	6B	534,921,073-534,927,092	guanyl-nucleotide exchange factor activity Rho guanyl-nucleotide exchange factor activity	–
5	rs10316	TGCAGATTGGGCTTGAGGAAATCTAACAAAACTTGGTGGATCGGCAAAGCCTGGATGAAATTCA_6	Seed number	7B	675,187,632-675,190,824	DNA binding	–

**Table 5:** Description of expected MTAs using imputed SNPs for agronomic traits of Iranian wheat accessions exposed to the well-watered conditions

Row	Marker	sequence	Trait	Chromosome	Position (bp)	Molecular process	Biological process
1	rs36032	TGCAGCTCATCACTAGTCTCGCGCTCGGGCAGCAGGACCGAGCTCGTCTCGCGCCCG_25	Grain yield and spike weight	1A	206,792,054-206,805,538	nucleotide binding DNA binding damaged DNA binding ATP binding mismatched DNA binding	DNA repair pyrimidine dimer repair cellular response to DNA damage stimulus negative regulation of reciprocal meiotic recombination
2	rs34075	TGCAGCGTTCGACCAGCTCATCACCCGCTTCCGAGATCGGAAGAGCGGGATCACCGACTGCCCA_19	Leaf greenness	1A	60,954,701-60,956,424	peroxidase activity oxidoreductase activity heme bindingmetal ion binding	response to oxidative stresshydrogen peroxide catabolic process oxidation-reduction process cellular oxidant detoxification
3	rs34314	TGCAGCTAACTAGCCTGAGATAATGCCAGCAACTCTGCTCGGTAGCTTTCTTAAGAAGGCCTTA_45	Spike length	4B	386,744,409-386,747,753	catalytic activity tRNA-specific adenosine deaminase activity zinc ion binding hydrolase activity	tRNA modification
4	rs736	TGCAGAAAGGTACCACTCATTCGTACATCACTCCAACTGATGTATGAAGGTTGTTCATGGCGAC_18	Spike weight	4B	481,233,765-481,237,258	hydrolase activity	phosphatidylinositol dephosphorylation
5	rs40819	TGCAGCTTCCATTTCATTCCTTCCTGCGCCATGGGTAACAAAAATTCAACTTCTTCAGTTAACA_32	Spike length	4B	667,563,369-667,564,460	protein binding	–
6	rs57386	TGCAGTATCGCAAGAGTAAAATGAAGTAGACAAAAACCTTGTATCATTAAAAGAGGCAGTCACC_18	Days to emergence	5A	467,397,067-467,403,109	serine-type endopeptidase activity serine-type peptidase activity serine-type exopeptidase activity	proteolysis
7	rs36808	TGCAGCTCCGTGTCAGTGGTGTCGCGGGTGAGGCTCTTCTGCTCATCGGCGCGGATCGGAACTT_44	Spike length	5B	287,752,969-287,780,293	ATP binding ATPase activity	–

**Table 6:** Description of expected MTAs using original SNPs for agronomic traits of Iranian wheat accessions exposed to the rain-fed conditions

Row	Marker	sequence	Trait	Chromosome	Position (bp)	Molecular process	Biological process
1	rs63808	TGCAGTTGAAGTCGCGGTGGATGACGGCGGGGGAGGTGTGCTCGTGCAGAAACTCCAGCGCGCG_49	Spike weight	1B	457,750,965-457,756,510	protein kinase activity ATP binding	protein phosphorylation
2	rs2237	TGCAGAAGGGGACGCCTCGGAATCTACGGCAGAGGACCGCCTCAGCGGCCTTCCCGACGGCGTC_30	Spike length	1B	26,855,662-26,857,170	protein binding (F-box domain)	–
3	rs26577	TGCAGCCTCCAATCGTGTACACACCTCCGTAAACAGATCTCGATTCTTCACTCCCTGTAGAGAG_5	Thousand kernel weight	2B	134,240,300-134,249,722	protein binding (Armadillo) Involved in membrane	–
4	rs15903	TGCAGCAGAGAATAATAGATGGAGGGAGGGGTGGTGCAAGTATAGCACCCGAGATCGGAAGAGC_41	Spike weight	2B	47,175,539-47,181,332	ADP binding (NB-ARC)	–
5	rs46075	TGCAGGCACGACCGCATGACCTTCTCGAACTTGGCGTCCTTGGCATGGGCGAGCGCAGACTCGA_25	Peduncle length	3B	44,856,819-44,857,576	enzyme inhibitor activity (Pectinesterase inhibitor domain)	negative regulation of catalytic activity
6	rs61706	TGCAGTGGGTCGTCGGAGCATCCAATCAGATCTCCACTACACGAACGAGACTAGCAGCAAGAGG_43	Thousand kernel weight	3B	783,413,489-783,414,580	GTPase activity GTP binding (Small GTPase AND Small GTP-binding protein domain)	
7	rs25700	TGCAGCCGCTCTTCGGCGGCTCTTGCATCGATGAGCTCGCGGGTGCGGGTAAGGGGCAAGTCGT_35	Plant height	5B	513,646,921-513,649,139	catalytic activity D-arabinono-1,4-lactone oxidase activity oxidoreductase activity flavin adenine dinucleotide binding FAD binding	oxidation-reduction process
9	rs57846	TGCAGTCAGAGATGATCAAGTTAAGGTCGTCGAACCCGTCATGGCAGCCGCCGCCGAGATCGGA_17	Seed number	5B	637,387,009-637,389,605	protein binding (BTB/POZ domain)	
10	rs46504	TGCAGGCATATGCTCGCCCCACATGTTCGTAGACAGGCTATCCTGCCGTTACGCATTGTGGTAC_30	Grain filling period	6B	534,921,073-534,927,092	guanyl-nucleotide exchange factor activity Rho guanyl-nucleotide exchange factor activity (PRONE domain AND protein binding	
11	rs30520	TGCAGCGCGACCCCTCTGCTGGCGAGCTGGGTTGGCCCATATATGTCTGCTTATTTTATAAAAA_57	Days to emergence	6B	532,043,561-532,045,921	anaphase-promoting complex binding ubiquitin-protein transferase activator activity	positive regulation of ubiquitin protein ligase activity
12	rs51526	TGCAGGGTACGTGAGTGATTAAACTGGCTGAGTTAATTGTGATCGGCATTTGATGGTTATGGCC_47	Grain yield	6B	664,500,180-664,501,715	–	asymmetric cell division
13	rs56337	TGCAGTACCGCTCTTCCCGAGCTGGCACTACTGTTCCACCCGTCCAACGATCTGTTGGGGCATC_32	Grain yield	7A	80,142,837-80,144,941	galactoside 2-alpha-L-fucosyltransferase activity (Xyloglucan fucosyltransferase	fucosylation cell wall biogenesis
14	rs53016	TGCAGGTCCCATGGCCTCTACCATAGTCGAACGGAGGTGGATGCGCTTTGAGGTGGATGCCTGA_35	Grain filling period	7B	15,713,548-15,714,633	DNA binding DNA-binding transcription factor activity (NA-binding domain superfamily, AP2/ERF domain)	regulation of transcription, DNA-templated

**Table 7:** Description of expected MTAs using imputed SNPs for agronomic traits of Iranian wheat accessions exposed to the rain-fed conditions

Row	Marker	sequence	Trait	Chromosome	Position (bp)	Molecular process	Biological process
1	rs64750	TGCAGTTTATGTACGAACTTTGAGAATTCTCATCAGTGGCCAAACGCCCAAACTAACAATTGAA_34	Canopy temperature	4A	630,897,051-630,899,273	DNA binding (3 DNA binding domain)	transcription, DNA-templated regulation of transcription, DNA-templated
2	rs11116	TGCAGCAAATTAATCTAGCTTTTAGTTTCCTTCAGGTATTTTGGATATGCCAGCAAATCGAAAG_29	Peduncle length	4A	739,791,132-739,802,785	ADP binding (NB-ARC)	–
3	rs736	TGCAGAAAGGTACCACTCATTCGTACATCACTCCAACTGATGTATGAAGGTTGTTCATGGCGAC_18	Spike weight	4B	481,233,765-481,237,258	hydrolase activity	phosphatidylinositol dephosphorylation
4	rs55557	TGCAGGTTTTGCCTAAGAAAAACTCAGAATTCACTCAAAAAAATCAGATTGCTGTAAACTGCAC_15	Canopy temperature	4B	613,031,990-613,041,407	drug transmembrane transporter activity antiporter activity (Multi antimicrobial extrusion protein)	drug transmembrane transport transmembrane transport
5	rs50187	TGCAGGGCAGTCGAAGCAGTTGCTGGGTCAGAGGCGTGGAGTTGCACTGGAGCAACAGGAGTCG_54	Spike length	4B	222,603,782-222,615,097	transmembrane transporter activity (Major facilitator, sugar transporter-like)	transmembrane transport
6	rs41689	TGCAGCTTGTCGGTCCTCTCCGACATGGCGTCGAGCACCCGCCGAGTCTGGGCCGAGGGTTTGG_15	Leaf greenness	5B	334,871,156-334,874,981	catalytic activity ATP binding zinc ion binding pyridoxal phosphate binding cysteine desulfurase activity (Cysteine desulfurase IscS)	iron-sulfur cluster assembly [2Fe-2S] cluster assembly
7	rs59282	TGCAGTCGTGGATAATGCACCTTGCGGTGTCAGGGGGTGACGTCAGCGATGAGTCCACCG_39	Days to heading	5B	11,550,484-11,556,238	catalytic activity hydrolase activity, hydrolyzing O-glycosyl compounds alpha-galactosidase activity hydrolase activity hydrolase activity, acting on glycosyl bonds raffinose alpha-galactosidase activity (Glycoside hydrolase, family 27)	carbohydrate metabolic process metabolic process
8	rs44154	TGCAGGAGCACCAGCGCGGCAGCGGTGGCGACGACGGGGCTACCAGCTGCCCGCCGAGATCGGA_20	Spike length	5B	363,662,473-363,670,095	catalytic activity hydrolase activity, hydrolyzing O-glycosyl compounds cellulase activity hydrolase activity hydrolase activity, acting on glycosyl bonds	polysaccharide catabolic process carbohydrate metabolic process metabolic process cellulose catabolic process
9	rs17806	TGCAGCAGGCAAGGTATCTCCAGGCGAACTATATCATCGCAATATACGAGCTTCAGGTGCTCCA_61	Days to heading, anthsis and physiological maturity	5B	457,966,329-457,970,659	protein binding (F-box-like domain superfamily)	–
10	rs25700	TGCAGCCGCTCTTCGGCGGCTCTTGCATCGATGAGCTCGCGGGTGCGGGTAAGGGGCAAGTCGT_35	Peduncle length	5B	513,646,921-513,649,139	catalytic activity D-arabinono-1,4-lactone oxidase activity oxidoreductase activity flavin adenine dinucleotide binding FAD binding	oxidation-reduction process

### Mining of highly associated favorable alleles

In the current study, SNPs with positive effects, causing an increase in grain yield, seed number, thousand kernel weight, grain filling period, spike weight, leaf greenness and the reduction in the days to emergence, days to heading, days to anthesis, days to physiological maturity, canopy temperature, plant height, peduncle length and spike length were defined as favorable alleles. The phenotypic effect of strongly associated SNPs under well-watered conditions using both original and imputed dataset was quantified using *ai*, where a reduction was observed for rs62576 by 0.72 in canopy temperature, rs34314 by 1.09, rs40819 by 0.70, rs36808 by 0.50 in spike length, and rs57386 by 0.11 in days to emergence (Table 8). The positive increasing effect of rs48893 and rs10316 on seed number per spike was 0.96 and 1.20, respectively. Moreover, thousand kernel weight, grain filling period, grain yield and leaf greenness were increased by 0.77 g, 0.90d, 0.01 g, and 1.82, respectively. Spike weight was associated with two markers, rs36032, and rs736, which increased the trait by 0.02 g and 0.33 g, respectively. The phenotypic variance explained (PVE) by the associated SNPs ranged from 7 to 40% (Table 8). The SNPs rs48893, rs10316, and rs36032 all show relatively high variance explained for the associated traits.

**Table 8:** The effect of favorable alleles on agronomic traits of Iranian wheat accessions exposed to the well-watered conditions

SNPs	Trait	Marker	*ai*	Typical accession	Allele	Favorable allele	MAF	p(−log10)	*R*^*2*^
Original	CT	rs62576	−0.72	623,266	A/G	A	0.14	3.68	0.40
	SN	rs48893 rs10316	0.96 1.20	624,251	C/T C/T	T C	0.32 0.14	3.51 3.59	0.24 0.20
	TKW	rs4772	0.77	623,345	A/C	C	0.43	3.23	0.11
	GFP	rs46504	0.90	626,158	A/C	C	0.19	4.30	0.13
Imputed	GY	rs36032	0.01	622,098	C/T	C	0.10	3.16	0.21
	SW	rs36032 rs736	0.02 0.33	622,098 Neishabour	C/T A/G	C G	0.10 0.11	3.16 3.56	0.21 0.10
	SL	rs34314 rs40819 rs36808	−1.09 −0.70 −0.50	Neishabour Chamran 621,650	C/T G/A A/G	T A G	0.04 0.06 0.39	4.71 4.29 3.41	0.12 0.12 0.10
	DE	rs57386	−0.11	625,081	A/C	A	0.22	3.61	0.07
	LG	rs34075	1.82	627,852	A/C	A	0.26	3.88	0.15

**Fig. 6:**
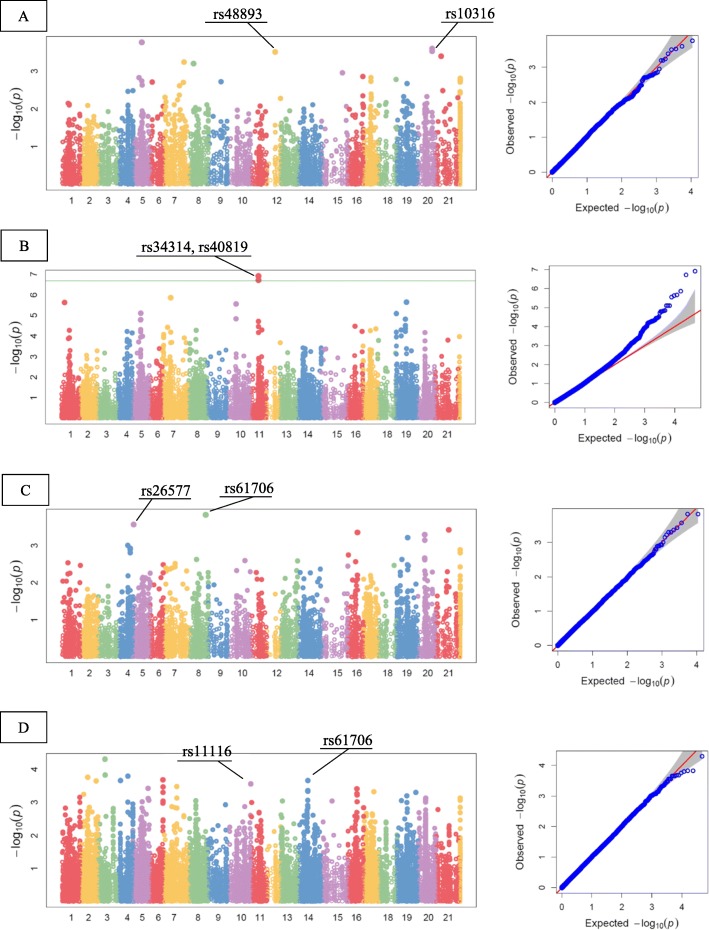
Manhattan and QQ-plots of highly associated haplotypes for agronomic traits under well-watered and rain-fed conditions. A) seed number per spike, B) spike length, C) thousand kernel weight, D) peduncle length. X axis represents chromosomes: 1)1A, 2) 1B, 3) 1D, 4) 2A, 5) 2B, 6) 2D, 7) 3A, 8) 3B, 9) 3D, 10) 4A, 11) 4B, 12) 4D, 13) 5A, 14) 5B, 15) 5D, 16) 6A, 17) 6B, 18) 6D, 19) 7A, 20) 7B, 21)7D

As illustrated in Table 9, under rain-fed conditions, 3, 2, 2, 2, 1, and 1 of the original SNPs were positively associated to spike weight (by 0.02, 0.03, and 0.03 g), thousand kernel weight (by 1.59, and 1.90 g), grain yield (by 0.03 and 0.03 g), and grain filling period (by 1.31, and 1.57 d), seed number per spike (by 0.58), and leaf greenness (by 1.69). In contrast, rs2234, rs25700, rs46075, and rs30520 had positive effects on spike length (by 0.53 cm), plant height (by 11.06 cm), peduncle length (by 1.76 cm), and days to emergence (by 0.28 d). However, the number of favorable alleles for spike length, spike weight, peduncle length, days to heading, days to anthesis, days to physiological maturity, canopy temperature and leaf greenness were 2, 1, 2, 2, 1, 1, 2, and 1, respectively. Rs11116 and rs25700 decreased peduncle length by 2.66 and 4.56 cm, rs59282 and rs17806 also decreased days to heading, anthesis, and maturity by 4.24, 7.59, 7.04, and 6.78 d and rs64750 caused a decline in canopy temperature by 2.61 °C. Under rain-fed conditions, PVE ranged from 7 to 38%. SNPs associated with grain yield, spike weight and seed number all explained a considerable proportion of the phenotypic variance. Moreover, the SNP rs17806 adjusted days to anthesis and physiological maturity by about 36 and 38%, respectively. Manhattan and QQ-plots of highly associated haplotypes for agronomic traits are shown in Fig. 6.

**Table 9:** The effect of favorable alleles on agronomic traits of Iranian wheat accessions exposed to the rain-fed conditions

SNPs	Trait	Marker	*ai*	Typical accession	Allele	Favorable allele	MAF	p(−log10)	*R*^*2*^
Original	SW	rs63808 rs15903 rs51526	0.02 0.03 0.03	623,125 BAHAR 623,125	A/G A/G A/G	G A A	0.15 0.32 0.19	3.60 3.47 3.26	0.32 0.32 0.32
	SL	rs2237	−0.53	627,057	A/G	G	0.17	4.62	0.13
	TKW	rs26577 rs61706	1.57 1.90	625,123 623,909	C/T A/G	T G	0.48 0.21	3.57 3.82	0.09 0.09
	SN	rs57846	0.58	Bahar	A/T	T	0.22	3.43	0.37
	GY	rs51526 rs56337	0.03 0.03	627,410 625,047	A/G G/T	A G	0.19 0.25	3.44 3.91	0.21 0.22
	PH	rs25700	−11.06	623,318	C/G	G	0.14	3.93	0.24
	PL	rs46075	−1.76	623,139	C/T	C	0.19	3.51	0.09
	GFP	rs46504 rs53016	1.31 1.57	626,360 623,905	A/C G/T	C T	0.19 0.16	3.63 5.21	0.09 0.11
	DE	rs30520	−0.28	626,825	A/G	G	0.13	3.41	0.07
	LG	rs33549	1.69	Gascogne	A/G	G	0.19	3.65	0.10
Imputed	SL	rs50187 rs44154	−0.39 − 0.14	626,924 627,057	A/G A/G	A G	0.22 0.21	4.04 3.42	0.14 0.13
	SW	rs736	0.29	Alvand	A/G	G	0.11	3.81	0.32
	PL	rs11116 rs25700	−2.66 −4.56	627,410 623,344	C/T C/G	C G	0.12 0.11	3.55 3.65	0.08 0.08
	DH	rs59282 rs17806	−4.24 −7.59	Dastjerdi Kavir 624,818	A/C C/T	C T	0.23 0.11	3.45 3.69	0.36 0.36
	DA	rs17806	−7.04	Shanghai7 Kavir	C/T	T	0.11	3.30	0.38
	DM	rs17806	−6.78	Frontana 624,818	C/T	T	0.11	3.44	0.32
	CT	rs64750 rs55557	−2.61 −0.39	624,315 622,105	A/G A/T	G T	0.03 0.49	4.32 3.58	0.11 0.09
	LG	rs41689	0.82	Roshan 623,344	C/G	C	0.24	3.83	0.12

## Discussion

Improving wheat grain yield is a high priority of wheat breeders in order to meet increasing demands worldwide. In the current study, we have explored the diversity of the Iranian hexaploid wheat population and performed association mapping studies for a number of important agronomic traits. These traits influence grain yield either directly or indirectly under well-watered or rain-fed conditions. Significant positive or negative relationships were observed among these traits, which can be used to gauge their impact on target traits like grain yield and grain yield-related attributes. Given that most agronomic traits are polygenic and drought tolerance is a complex mechanism involving many pathways, we focused on three categories of agronomic traits employing a large diversity panel. Furthermore, using a diverse gene pool help increase the resolution of association mapping. We, therefore, tested both historical and modern varieties as well as representative landraces from different climate zones in order to include sufficient genetic variation to be able to map trait-relevant variation.

Most Iranian varieties originate from International Maize and Wheat Improvement Center (CIMMYT) materials with twenty-four varieties in advanced and segregating lines, originating from this center. At least, forty varieties were obtained through a cross-breeding program where one of the CIMMYT advanced lines was one of the parents (Additional file 1: Table S1). A large number of these varieties were released after green revolution. A previous study on historical wheat varieties from Pakistan illustrated the considerable contribution of CIMMYT germplasm, which has been used in the development of Iranian varieties as well [21]. We identified three clusters of varieties with mixed genetic backgrounds with no clear relation to the release year. The relatively small number of the varieties derived from Iran suggests a relatively narrow utilization of Iranian landraces among the current and old varieties, which could be a substantial genetic bottleneck.

In line with earlier studies, we identified most SNPs in the B and A genomes whereas the younger D genome showed a lower number of SNPs [32, 33]. We observed the same trend also for the number of marker pairs in LD, where SNPs mapping to the B genome were approximately three times more common than those mapping to the D genome. The most significant marker pairs were observed on chromosomes 2B and 3B in both, varieties and landraces (Table 2). The higher diversity seen in the A and B genomes could be the result of their older evolutionary background and due to gene flow from *T. turgidum* as opposed to lack of gene flow from *Ae. tauschii* to bread wheat [34, 35]. Moreover, a bottleneck effect likely occurred due to strong selection among ancestral hexaploid landraces in modern varieties breeding programs and this may have further effects on the D genome. Such a bottleneck would result in a reduction in the effective population size, which increases the rate of loss of low-frequency alleles in the A and B genomes, and on the other hand, a higher proportion of rare alleles in the D genome indicate a reduction in the allelic variant for younger genome [36]. Our results show that most markers that are in significant LD are located at distances < 10 cM. However, LD and marker distances across the D genome were much higher than in the other two sub-genomes. The greater extent of linkage across all genomes in varieties clearly indicates the effect of selection during the history of those accessions (Table 2). Selection, recombination, mutation, genetic drift, mating systems and population relatedness are all major factors, which influence linkage disequilibrium [37–39]. The fact that varieties show significantly overall higher LD compared to landraces, in particular in the D genome, is likely the result of selection during breeding for important agronomic traits [40, 41].

While mapping traits with low heritability may not result in a desirable gain, utilizing highly correlated traits can increase power and therefore help with the rapid advancement of breeding programs. Although grain yield is the most reliable selection criterion in different target environments, particularly for water deficit conditions, the complex genetic architecture of this trait has thus far limited direct genome-based selection. However, the pleiotropic effect of genes controlling this trait and the close connection between grain yield and drought tolerance mechanisms highlights the role that other morphological, phenological and physiological traits play and which should be considered in any selection strategy.

Among 313 and 394 identified MTAs for the original and imputed SNPs, respectively, 86 and 76 falls within coding genes with *P*-values < *0.001*. To eliminate possible false-positive associations, we selected the most strongly associated markers which yielded 19 and 17 markers located across all chromosomes and were identified in both environments using the two SNP datasets. Using the original SNPs under well-watered conditions, we identified QTLs for canopy temperature (1A), seed number (4D, and 7B), thousand kernel weight (5B), and grain filling period (6B). Using the imputed SNPs, we observed QTLs for grain yield and spike weight on chromosomes 1A and 4B, for leaf greenness on chromosome 1A, three QTLs on 4B and 5B for spike length and one QTL on 5A for days to emergence. These results are in line with previously detected QTLs for spike length [42], grain yield [42], seed number per spike [43] and thousand kernel weight [44].

For rain-fed conditions, we identified QTLs on chromosomes 1B, 2B for spike weight, 6B and 7A for grain yield, 1B for spike length, 2B and 3B for thousand kernel weight, 3B for peduncle length, 5B for plant height, 5B for leaf greenness, 5B for seed number per spike, 6B and 7B for grain filling period and 6B for days to emergence. These results are in line with findings by Ain et al. [21] for plant height, thousand kernel weight and grain yield. Bossolini et al. [45] and Acuna-Galindo et al. [46] identified stable QTLs on chromosomes 6B and 5B for grain yield and plant height, respectively. MTAs for seed number per spike on chromosome 5B have been previously reported [47, 48]. Neumann et al. [14] reported a QTL on chromosome 2B for spike weight. Using imputed SNPs and phenotypic data under rain-fed conditions, all identified MTAs were located on chromosomes 4A, 4B and 5B, with QTLs for canopy temperature (4A and 4B), peduncle length (4A, and 5B), spike weight (4B), spike length (4B and 5B), leaf greenness (5B) and days to heading, anthesis, maturity (5B). These results are in agreement with previously reported QTLs for these traits [14, 33, 47, 48]. In addition, we also identified a number of novel chromosomal regions that harbored MTAs for physiological parameters and phenological growth stages. For instance, under well-watered conditions, rs62576 (1A), rs34075 (1A) and rs57386 (5A) were associated with canopy temperature, leaf greenness and days to emergence. Under rain-fed conditions, markers rs64750 (4A) and rs55557 (4B) were associated with canopy temperature whereas rs4607 (3B) and rs41689 (5B) were associated with peduncle length and leaf greenness, respectively. Moreover, marker rs17806 (5B) has pleiotropic effects on days to heading, anthesis, and physiological maturity.

Although imputation of missing data significantly increased the potential number of MTAs, mainly on the A and B genomes, the fraction of SNPs presents within coding genes declined, from about 27% in the original SNP data set to 19.6% for the imputed dataset. This suggests that most SNPs with missing data are located in noncoding DNA regions where read mapping and SNP calling are known to be more problematic in most plant genomes. Dissecting strongly associated chromosomal regions through, for instance, positional cloning to identify putative causal genes is the next logical step following association mapping studies. Apart from using comparative genomics approaches to identify the function of associated genes, independent functional validation is also required to guarantee the success of either positional cloning or transgenic experiments [21, 49–51]. We obtained the flanking sequence of putative SNPs and aligned them against the IWGSC RefSeq v1.0. This information showed that most genes identified through the association study are involved in important biosynthesis pathways such as oxidation-reduction, carbohydrate metabolism, ion transportation and cell wall biogenesis. The protein encoded by these genes are mostly involved in DNA-binding, ATP-binding, peroxidase activity, protein kinase activity, metal ion binding, enzyme inhibitor activity, serine-type endopeptidase activity, hydrolase activity, antiporter activity and transmembrane activity. Such associations have also been reported in earlier research [52–58]. These genes are all located in chromosomal regions, which show a strong association with important agronomic traits and they can thus be considered as suitable target genes for future breeding programs. We calculated the phenotypic effect of favorable alleles, as described by Dong et al. [59] to show that they affected grain yield only slightly but had much larger effect on thousand kernel weight, spike length and leaf greenness. On the other hand, alleles that contribute to an improvement in thousand kernel weight and leaf greenness also have pleiotropic effects thereby decreasing plant height, peduncle length and canopy temperature under stressful conditions. Most identified MTAs exist across genes which are involved in multilayer processes and complex networks, therefore their minor impact on agronomic traits is not too far-fetched.

## Conclusions

In the present study, GWAS was performed for important agronomic traits of bread wheat in a diverse panel of 298 varieties and landraces of Iran collection. The highest number of marker pairs in both varieties and landraces was observed on B genome. In total, 313 and 394 MTAs were identified for 14 phenological, agronomic and physiological traits using original and imputed SNPs, respectively. The identified association between markers and traits generally lied in a range of 10^− 3^ and 10^− 4^. It seems that complex inheritance of such quantitative traits and high number of controlling genes exclude greater association. However, a major part of found MTAs explained more than 20% of total phenotypic variation for relevant traits. Although, further studies are required to validate the detected markers in this study using other populations and environments. Gene ontology of identified markers in original and imputed SNPs showed approximately 27% of these markers represent within coding genes, thereby have potential to be used in genome-based breeding of new varieties. Although imputation of missing data could increase the number of associated markers, the percentage of MTAs located in coding regions was decreased across different sub-genomes. The identified markers in this study could provide useful genetic resources to initiate marker-assisted selection, fine mapping and cloning of the underlying genes and QTLs.

## Methods

### Plant material and experiment conditions

A set of 320 Iranian wheat accession, including 102 varieties released between 1942 and 2014, and 218 landraces collected between 1931 and 1968 (Additional file 1: Table S1) were tested under a well-watered system and rain-fed conditions using an alpha-lattice design with two replicates at the agricultural research lands of the Department of Agronomy and Plant Breeding, University of Tehran. Plant materials were kindly provided by the University of Tehran and Seed and Plant Improvement Institute (SPII), Karaj, Iran. Both phenotypic and genotypic data were available for 298 accessions (90 varieties and 208 landraces). The field site is located at N 35′.80° and E 50′.95° in Karaj, Iran, and experiments were conducted during the cropping seasons of 2016–17 and 2017–18 (weather conditions are given in Additional file 3: Fig. S1).

### Field trial

Plant development was scored according to the Zadoks scale and included i) days to emergence (Zadoks 12), ii) days to heading (Zadoks 50), iii) days to anthesis (Zadoks 65), iv) days to physiological maturity (Zadoks 91), and v) grain filling period when half of each plot had reached to corresponding stages. The Soil Plant Analysis Development (SPAD, Minolta Camera Co., Osaka, Japan, SPAD502 Plus Chlorophyll Meter) and LIHERO Infrared thermometer were used to measure leaf greenness and canopy temperature at Zadoks 60, respectively. Grain yield and related traits including spike weight, spike length, seed number per spike and thousand kernel weight were measured after harvesting for both years.

### Genotyping by sequencing and imputation method

The development and sequencing of a GBS library for the Iranian wheat have previously been described by Alipour et al. [30]. Briefly, after trimming sequencing reads to 64 bp and grouping them into sequence tags, SNPs were identified using internal alignment allowing for mismatch up to 3 bp. The UNEAK (Universal Network-Enabled Analysis Kit) GBS pipeline was used for SNPs calling, where reads with a low-quality score (<15) and SNPs with low minor allele frequency <1% were removed to avoid false-positive markers arising from sequencing errors. The data was also subjected to imputation using BEAGLE v3.3.2 [60] based on available allele frequencies obtained after specifying the haplotype phase for all individuals. Four different reference genomes were assessed during imputation and W7984 reference genome was shown to have the greatest imputation accuracy [31]. The LD decay of different chromosomes was obtained using the ggplot2 package in RStudio [61] based on LOESS regression.

### Population structure and kinship matrix

Population structure in the sample was estimated using STRUCTURE v.2.3.4 [62] with an admixture model and with a burn-in and simulation phase consisting of 10,000 steps for values of K = 1 to 10. ∆K was plotted for consecutive K values and used to determine the most likely number of subpopulations. The values of observed and expected allele frequencies were used to calculate LD among markers in TASSEL v.5 [63]. A structure matrix (Q-matrix) was then obtained for all accessions used for association studies. To determine the relationship between varieties and landraces, a neighbor-joining tree was constructed based on a pairwise distance matrix calculated in TASSEL v.5 [63] and visualized using Archaeopteryx (https://sites.google.com/site/cmzmasek/home/software/archaeopteryx).

### Genome-wide association study

We used both general linear model (GLM) and mixed linear model (MLM) to obtain the unbiased estimation of marker effects. The MLM approach resulted in the most accurate association of marker-traits and different versions of the MLM model, including Q, K or Q + K, were used to control both effects of population structure (Q) and more diffused relationships (K) among accessions using TASSEL v.5 [63]. The GAPIT package [64] was used to perform association mapping for the MLM model in RStudio [61]. Results from both TASSEL [63] and GAPIT [64] evaluated based on the significance of associated loci using *t*-tests. In general, GAPIT [64] provided a stronger control of confounding effects. We, therefore, only reported results from GAPIT [64]. In the MLM model, individuals are considered as random effects and the relatedness among individuals is conveyed through a kinship matrix. To perform cluster analysis, kinship matrix elements were used as similarity measures and clusters visualized using unweighted pair group method with arithmetic mean (UPGMA) through the heat map plot. A Manhattan plot is a visualized form of associations between phenotype and genotype, in which SNPs are ordered based on their chromosome and base-pair positions. In a Manhattan plot, the x-axis thus represents the genomic position of each SNP and y-axis represents the negative logarithm of the *P*-value generated from the F-test for testing H_0_. Here, both heat map and Manhattan plots were obtained from an enhanced comparison scenario using the GAPIT package [64].

### Gene annotation

Sequences surrounding all significantly associated SNPs were obtained from the wheat 90 K SNP database [65] used for assessing gene annotation using Gramene (http://www.gramene.org/) by aligning them to the IWGSC RefSeq v1.0 annotation (https://wheat-urgi.versailles.inra.fr/Seq-Repository/Annotations). The function of putative genes was explored by investigating the pathways which the encoded enzymes were involved in. After aligning SNPs sequences to the reference genome, overlapping genes with the highest identity percentage and blast score were selected for further processing. The gene ontology of each selected gene, including molecular function and biological process, were extracted from the ensemble-gramene database (http://ensembl.gramene.org).

### Phenotyping data analysis and calculation of favorable allele effect

Phenotypic data were analyzed using SAS v.9.4 [66] separately for the two environments. The adjusted means were then obtained from the alpha-lattice design used for advanced linear analysis. Adjusted means were estimated using GLM and Mixed procedures. The phenotypic effect of favorable alleles (*ai*) was estimated using the following formula:

$$ ai=\left(\frac{\varSigma {x}_{ij}}{n_i}\right)-\left(\frac{\varSigma {N}_K}{n_K}\right) $$

where, *x*_*ij*_ is the phenotypic value of the *j*th individual for the *i*th allele, *n*_*i*_ is the number of individuals carrying the *j*th allele, *N*_*K*_ is the nth individual phenotypic value for all entries, and *n*_*K*_ is the number of individuals. Positive and negative effects of all alleles are represented by *ai* >0, and *ai* <0, respectively.

## Supplementary information

**Additional file 1 Table S1.** The information of Iranian wheat accessions including varieties released between 1942 and 2014, and landraces collected between 1931 and 1968

**Additional file 2 Table S2.** Analysis of variance for agronomic traits of Iranian wheat accessions exposed to the well-watered conditions over two years. **Table S3.** Analysis of variance for agronomic traits of Iranian wheat accessions exposed to the rain-fed conditions over two years. **Table S4.** Person’s coefficient of correlation between agronomic traits of Iranian wheat accessions over two years under well-watered conditions. **Table S5.** Pearson’s confidents of correlation between agronomic traits of Iranian bread wheat accessions over two years under rain-fed conditions.

**Additional file 3 Fig. S1.** Climate condition include precipitation and temperature of field trail site during the 2016–17 and 2017–18 cropping seasons. **Fig. S2.** The pattern of LD decay in different chromosomes of genome A in *T. aestivum* based on original SNPs. **Fig. S3.** The pattern of LD decay in different chromosomes of genome B in *T. aestivum* based on original SNPs. **Fig. S4.** The pattern of LD decay in different chromosomes of genome D in *T. aestivum* based on original SNPs.

**Additional file 4 Table S6.** Description of expected MTAs using original SNPs for agronomic traits of Iranian wheat accessions exposed to the well-watered condition. **Table S7.** Description of expected MTAs using original SNPs for agronomic traits of Iranian wheat accessions exposed to the rain-fed condition. **Table S8.** Description of expected MTAs using imputed SNPs for agronomic traits of Iranian wheat accessions exposed to the well-watered condition. **Table S9.** Description of expected MTAs using imputed SNPs for agronomic traits of Iranian wheat accessions exposed to the rain-fed condition.

## Data Availability

The datasets generated and analysed during the current study are available in Figshare. SNP data files for 5, 6, 7, and 8 runs can be each accessed at: https://figshare.com/s/92cd9f23d9dddb224304; https://figshare.com/s/b402031d86d969a7cb0f; https://figshare.com/s/83b4abe205a8a5409017; https://figshare.com/s/253e39735d226b78b6bd.

## Abbreviations

CT: Canopy temperature
DA: Days to anthesis
DE: Days to emergence
DH: Days to heading
DM: Days to physiological maturity
GBS: Genotyping by sequencing
GF: Grain filling period
GWAS: Genome-wide association study
GY: Grain yield
H: Plant height
LD: Linkage disequilibrium
LG: Leaf greenness
MAF: Minor allele frequency
MAS: Marker-assisted selection
MP: Marker pairs
MTA: Marker-trait associations
PL: Peduncle length
QTL: Quantitative trait loci
SL: Spike length
SN: Seed number per spike
SNP: Single nucleotide polymorphism
SW: Spike weight
TKW: Thousand kernel weight
