# Supplementary material for: Genome-wide association study of agronomic traits in bread wheat reveals novel putative alleles for future breeding programs
Source: BMC Plant Biol. 2019 Dec 5;19:541. doi: 10.1186/s12870-019-2165-4 (PMC6896361; doi:10.1186/s12870-019-2165-4)
Supplement: Supplementary file 1 — Additional file 1 Table S1. The information of Iranian wheat accessions including varieties released between 1942 and 2014, and landraces collected between 1931 and 1968 [file 12870_2019_2165_MOESM2_ESM.docx]

Table S2

Analysis of variance for agronomic traits of Iranian wheat accessions exposed to the well-watered conditions over two years

| S.O.V | DF | MS | | | | | | | | | | | |
| --- | --- | --- | --- | --- | --- | --- | --- | --- | --- | --- | --- | --- | --- |
|  |  | Days to emergence | Days to heading | Days to anthesis | Days to physiological maturity | Grain filling period | Leaf greenness | Canopy temperature | Spike weight | Spike length | Grain yield | Seed number | Thousand kernel weight |
| Year | 1 | 148759.69 ^**^ | 61272.45 ^**^ | 40017.70 ^**^ | 66010.05 ^**^ | 2784.8 ^**^ | 365.41 ^**^ | 14829.45 ^**^ | 66.82 ^**^ | 80.85 ^**^ | 60.14 ^**^ | 16852.5 ^**^ | 2516.2 ^**^ |
| Year × Replication | 2 | 79.21 | 0.27 | 96.95 | 1.86 | 124.62 | 36.08 | 170.22 | 1.06 | 20.78 | 0.68 | 64.35 | 2422.36 |
| Year × Rep × Block | 124 | 5.02 | 2.28 | 2.48 | 3.75 | 5.26 | 5.58 | 556.1 | 0.33 | 11.99 | 0.25 | 77.15 | 92.05 |
| Genotype | 319 | 4.09 ^**^ | 126.05 ^**^ | 124.51 ^**^ | 139.55 ^**^ | 22.27 ^**^ | 81.35 ^**^ | 1544.15 ^**^ | 0.64 ^**^ | 14.62 ^**^ | 0.37 ^**^ | 169.41 ^**^ | 173.68 ^**^ |
| Year × Genotype | 319 | 4.28 ^**^ | 31.13 ^**^ | 31.70 ^**^ | 44.37 ^**^ | 22.31 ^**^ | 46.63 ^**^ | 2021.31 ^**^ | 0.37 ns | 11.20 ns | 0.26 ^**^ | 134.81 ^**^ | 135.66 ^*^ |
| Error | 514 | 2.71 | 1.86 | 2.35 | 4.11 | 3.41 | 5.44 | 2.70 | 0.33 | 10.88 | 0.2 | 73.34 | 111.66 |
| C.V | - | 6.66 | 0.78 | 0.83 | 0.96 | 6.78 | 4.55 | 6.91 | 22.83 | 30.39 | 25.46 | 19.69 | 25.70 |

^*^ P <0.05, ^**^ P <0.01 and ns: non-significant

Table S3

Analysis of variance for agronomic traits of Iranian wheat accessions exposed to the rain-fed conditions over two years

| S.O.V | DF | MS | | | | | | | | | | | | |
| --- | --- | --- | --- | --- | --- | --- | --- | --- | --- | --- | --- | --- | --- | --- |
|  |  | Days to emergence | Days to heading | Days to anthesis | Days to physiological maturity | Grain filling period | Leaf greenness | Canopy temperature | Spike weight | Spike length | Grain yield | Seed number | Thousand kernel weight | Straw weight |
| Year | 1 | 211099.88 ^**^ | 364557.52 ^**^ | 347655.49 ^**^ | 276830.45^**^ | 4029.25^**^ | 14986.44 ^**^ | 17647.59 ^**^ | 17.02 ^**^ | 23.95 ^**^ | 8.47 ^**^ | 59.08 ns | 10705.25 ^**^ | 1.48 ^**^ |
| Year × Replication | 2 | 0.70 | 3.77 | 56.24 | 99.76 | 280.82 | 66.43 | 69.28 | 2.47 | 14.73 | 1.07 | 1696.28 | 182.92 | 0.88 |
| Year × Rep × Block | 124 | 28.17 | 15.84 | 3.35 | 4.84 | 1.64 | 4.34 | 3.15 | 0.19 | 2 | 0.12 | 63.76 | 62.80 | 0.08 |
| Genotype | 319 | 17.98 ^**^ | 148.91 ^**^ | 124.86 ^**^ | 144.50 ^**^ | 26.64^**^ | 88.07 ^**^ | 8.87 ^**^ | 0.43 ^**^ | 4.22 ^**^ | 0.18 ^*^ | 158.42 ^**^ | 107.81 ns | 0.17 ^**^ |
| Year × Genotype | 319 | 15.81 ^**^ | 52.07 ^**^ | 43.28 ^**^ | 59.25^**^ | 25.09^**^ | 76.32 ^**^ | 7.67 ^**^ | 0.20 ^**^ | 3.14 ^**^ | 0.12 ns | 66.26 ns | 76.79 ns | 0.11 ns |
| Error | 514 | 0.50 | 23.40 | 2.96 | 4.26 | 2.06 | 4.08 | 3.41 | 0.14 | 1.79 | 0.15 | 68.36 | 126.41 | 0.1 |
| C.V | - | 2.49 | 2.90 | 0.99 | 1.048 | 5.98 | 4.10 | 6.68 | 21.85 | 13.63 | 35.51 | 21.88 | 38.01 | 48.99 |

^*^ P <0.05, ^**^ P <0.01 and ns: non-significant

Table S4

Person’s coefficient of correlation between agronomic traits of Iranian wheat accessions over two years under well-watered conditions

| Trait | DE | DH | DA | DM | GFP | SW | SL | GY | SN | TKW | STW | LG | CT |
| --- | --- | --- | --- | --- | --- | --- | --- | --- | --- | --- | --- | --- | --- |
| DE | 1 |  |  |  |  |  |  |  |  |  |  |  |  |
| DH | -0.15^**^ | 1 |  |  |  |  |  |  |  |  |  |  |  |
| DA | -0.15^*^ | 0.99^**^ | 1 |  |  |  |  |  |  |  |  |  |  |
| DM | -0.20^**^ | 0.92^**^ | 0.92^**^ | 1 |  |  |  |  |  |  |  |  |  |
| GFP | -0.13^*^ | -0.060 | -0.11 | 0.30^**^ | 1 |  |  |  |  |  |  |  |  |
| SW | -0.07 | -0.12^*^ | -0.09 | -0.001 | 0.20^**^ | 1 |  |  |  |  |  |  |  |
| SL | -0.007 | 0.40^**^ | 0.39^**^ | 0.36^**^ | 0.003 | -0.06 | 1 |  |  |  |  |  |  |
| GY | -0.03 | -0.15^**^ | -0.13^*^ | -0.042 | 0.19^**^ | 0.94^**^ | -0.12^*^ | 1 |  |  |  |  |  |
| SN | -0.036 | -0.21^**^ | -0.18^**^ | -0.12^*^ | 0.13^*^ | 0.64^**^ | -0.11 | 0.67^**^ | 1 |  |  |  |  |
| TKW | -0.039 | -0.009 | -0.007 | 0.04 | 0.10 | 0.48^**^ | -0.10 | 0.55^**^ | -0.17^**^ | 1 |  |  |  |
| STW | -0.12^*^ | 0.008 | 0.02 | 0.08 | 0.15^*^ | 0.71^**^ | 0.08 | 0.41^**^ | 0.33^**^ | 0.13^*^ | 1 |  |  |
| LG | -0.040 | -0.12^*^ | -0.12^*^ | -0.07 | 0.13^*^ | 0.21^**^ | -0.17^**^ | 0.24^**^ | 0.17^**^ | 0.11 | 0.06 | 1 |  |
| CT | 0.086 | -0.22^**^ | -0.21^**^ | -0.21^**^ | -0.006 | -0.09 | -0.12^*^ | -0.04 | -0.008 | 0.08 | -0.16^**^ | -0.06 | 1 |

DE: Days to emergence, DH: Days to heading, DA: Days to anthesis, DM: Days to physiological maturity, GF: Grain filling period, H: Plant height, PL: Peduncle length, SW: Spike weight, SL: Spike length, GY: Grain yield, SN: Seed number per spike, TKW: Thousand kernel weight, STW: Straw weight, LG: Leaf greenness, CT: Canopy temperature, ^*^ P <0.05, ^**^ P <0.01

Table S5

Pearson’s confidents of correlation between agronomic traits of Iranian bread wheat accessions over two years under rain-fed conditions

|  | DE | DH | DA | DM | GFP | H | PL | SW | SL | GY | SN | TKW | STW | LG | CT |
| --- | --- | --- | --- | --- | --- | --- | --- | --- | --- | --- | --- | --- | --- | --- | --- |
| DE | 1 |  |  |  |  |  |  |  |  |  |  |  |  |  |  |
| DH | 0.10 | 1 |  |  |  |  |  |  |  |  |  |  |  |  |  |
| DA | 0.12^*^ | 0.95^**^ | 1 |  |  |  |  |  |  |  |  |  |  |  |  |
| DM | 0.06 | 0.87^**^ | 0.90^**^ | 1 |  |  |  |  |  |  |  |  |  |  |  |
| GFP | -0.12^*^ | -0.07 | -0.12^*^ | 0.33^**^ | 1 |  |  |  |  |  |  |  |  |  |  |
| H | -0.11 | 0.37^**^ | 0.41^**^ | 0.36^**^ | -0.06 | 1 |  |  |  |  |  |  |  |  |  |
| PL | -0.03 | 0.09 | 0.09 | 0.06 | -0.07 | 0.63^**^ | 1 |  |  |  |  |  |  |  |  |
| SW | -0.11 | -0.48^**^ | -0.47^**^ | -0.41^**^ | 0.10 | -0.18^**^ | -0.09 | 1 |  |  |  |  |  |  |  |
| SL | -0.13^*^ | 0.32^**^ | 0.30^**^ | 0.30^**^ | 0.02 | 0.18^**^ | 0.03 | -0.10 | 1 |  |  |  |  |  |  |
| GY | -0.08 | -0.46^**^ | -0.47^**^ | -0.44^**^ | .03 | -0.19^**^ | -0.06 | 0.80^**^ | -0.12^*^ | 1 |  |  |  |  |  |
| SN | -0.02 | -0.40^**^ | -0.40^**^ | -0.34^**^ | 0.08 | -0.18^**^ | -0.05 | 0.70^**^ | -0.10 | 0.56^**^ | 1 |  |  |  |  |
| TKW | -0.08 | -0.17^**^ | -0.20^**^ | -0.19^**^ | -0.004 | -0.09 | -0.03 | 0.25^**^ | -0.03 | 0.58^**^ | -0.26^**^ | 1 |  |  |  |
| STW | -0.08 | -0.27^**^ | -0.25^**^ | -0.18^**^ | 0.12^*^ | -0.08 | -0.09 | 0.74^**^ | -0.02 | 0.18^**^ | 0.52^**^ | -0.25^**^ | 1 |  |  |
| LG | -0.06 | 0.006 | 0.01 | 0.09 | 0.17^**^ | -0.09 | -0.11^*^ | 0.23^**^ | 0.07 | 0.10 | 0.16^**^ | -0.01 | 0.27^**^ | 1 |  |
| CT | 0.26^**^ | -0.09 | -0.09 | -0.10 | -0.03 | -0.26^**^ | -0.20^**^ | 0.002 | -0.17^**^ | -0.02 | -0.03 | -0.03 | 0.02 | 0.001 | 1 |

DE: Days to emergence, DH: Days to heading, DA: Days to anthesis, DM: Days to physiological maturity, GF: Grain filling period, H: Plant height, PL: Peduncle length, SW: Spike weight, SL: Spike length, GY: Grain yield, SN: Seed number per spike, TKW: Thousand kernel weight, STW: Straw weight, LG: Leaf greenness, CT: Canopy temperature, ^*^ P <0.05, ^**^ P <0.01
