# Supplementary material for: Genome-wide association study of agronomic traits in bread wheat reveals novel putative alleles for future breeding programs
Source: BMC Plant Biol. 2019 Dec 5;19:541. doi: 10.1186/s12870-019-2165-4 (PMC6896361; doi:10.1186/s12870-019-2165-4)
Supplement: Supplementary file 3 — Additional file 3 Fig. S1. Climate condition include precipitation and temperature of field trail site during the 2016–17 and 2017–18 cropping seasons. Fig. S2. The pattern of LD decay in different chromosomes of genome A in T. aestivum based on original SNPs. Fig. S3. The pattern of LD decay in different chromosomes of genome B in T. aestivum based on original SNPs. Fig. S4. The pattern of LD decay in different chromosomes of genome D in T. aestivum based on original SNPs. [file 12870_2019_2165_MOESM3_ESM.docx]

Figure S1. Climate condition including precipitation and temperature of field trail site during 2016-17 and 2017-18 cropping seasons


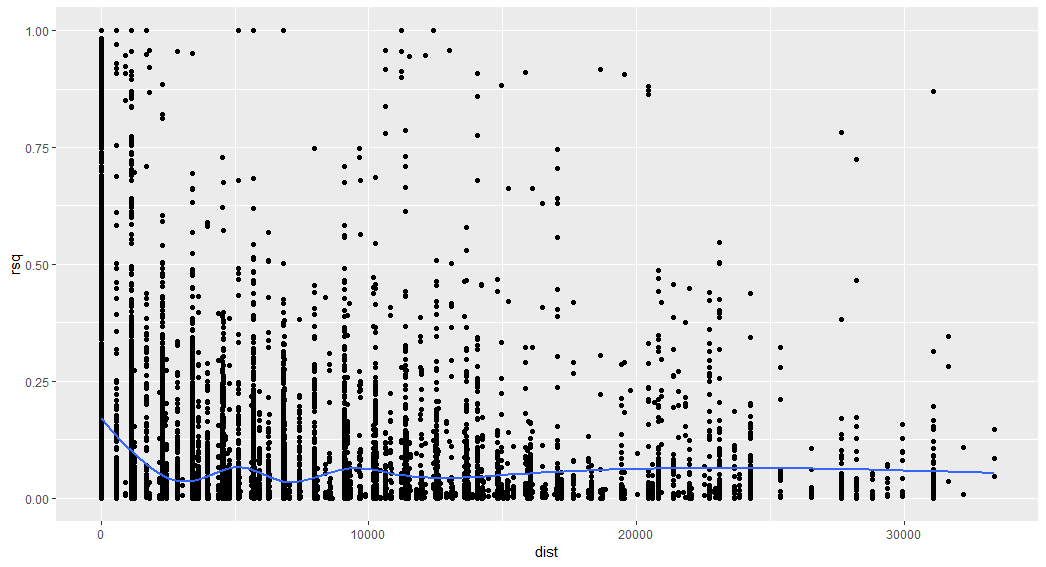

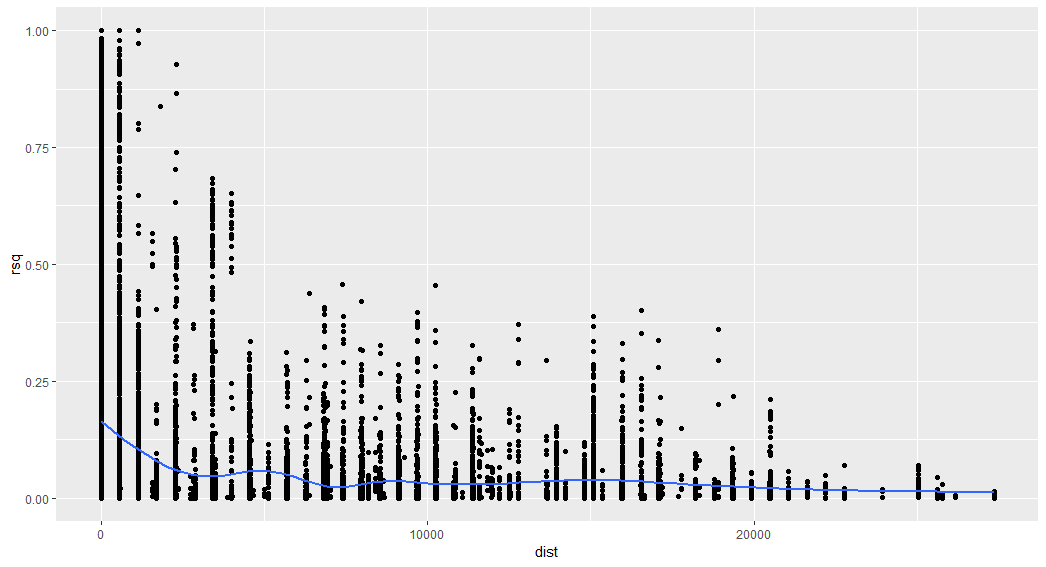


2A

1A


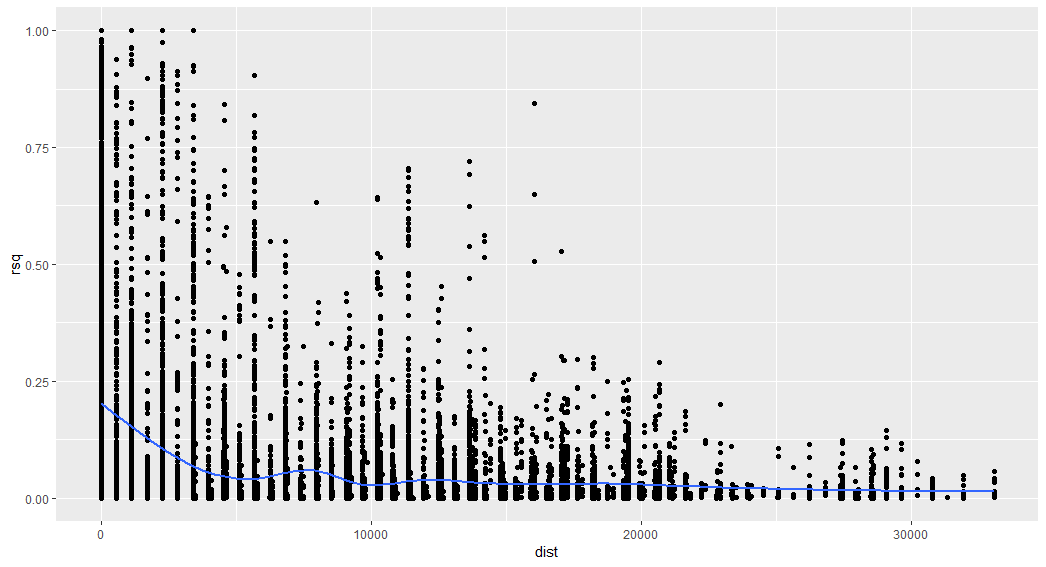

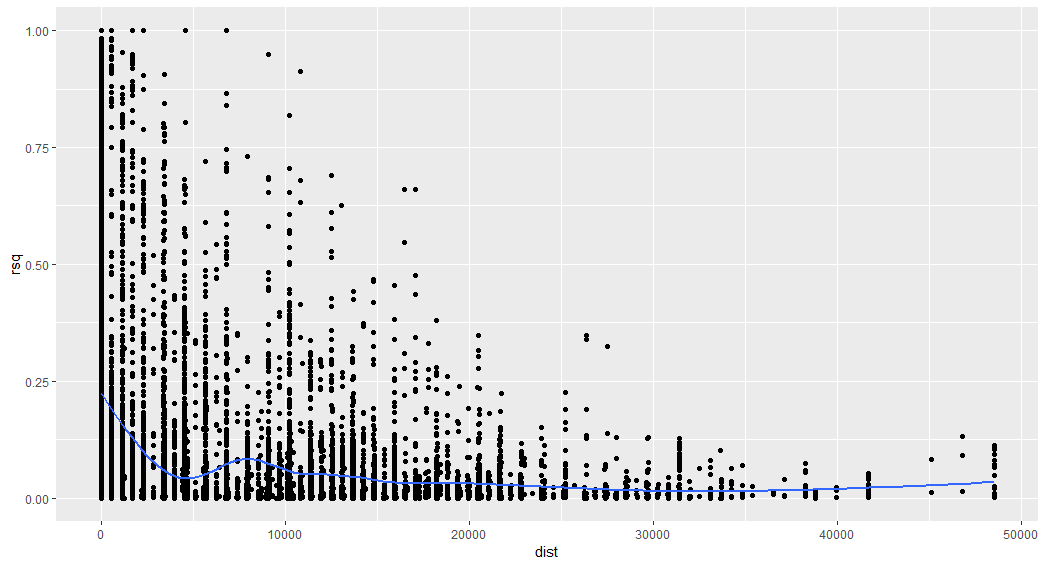


4A

3A


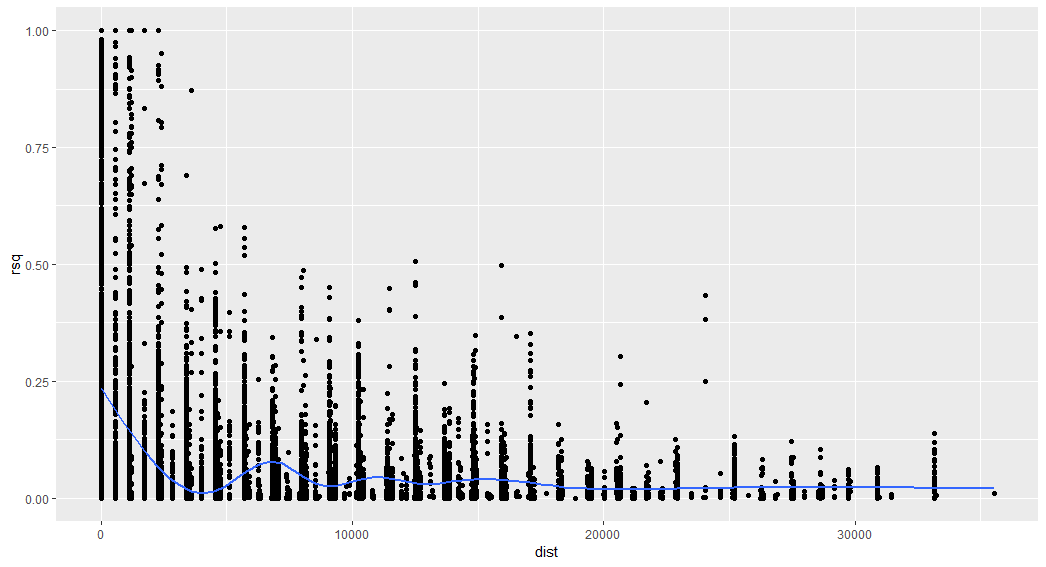

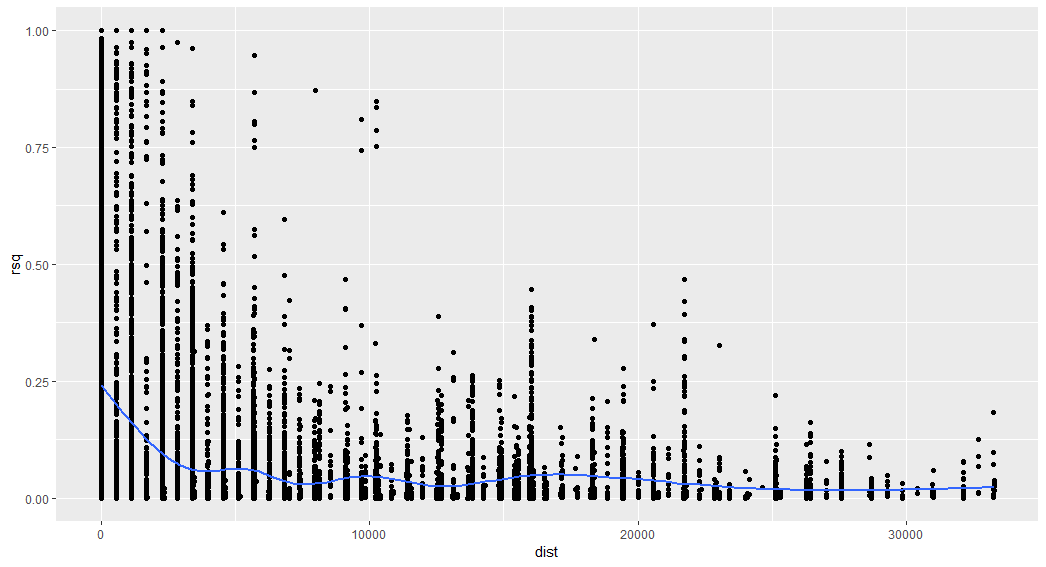


6A

5A


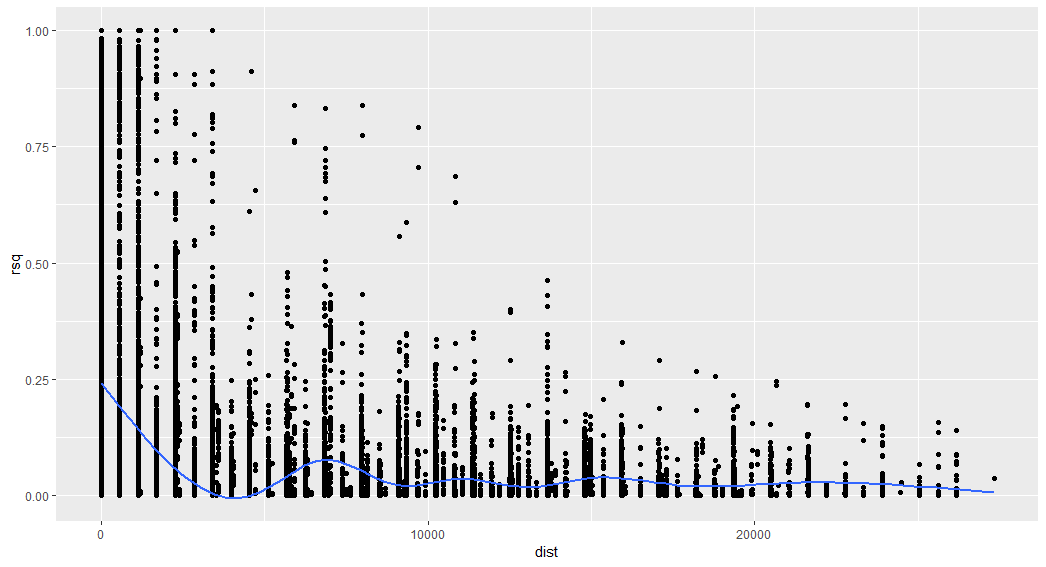


7A

Figure S2. The pattern of LD decay in different chromosomes of genome A in *T. aestivum* based on original SNPs


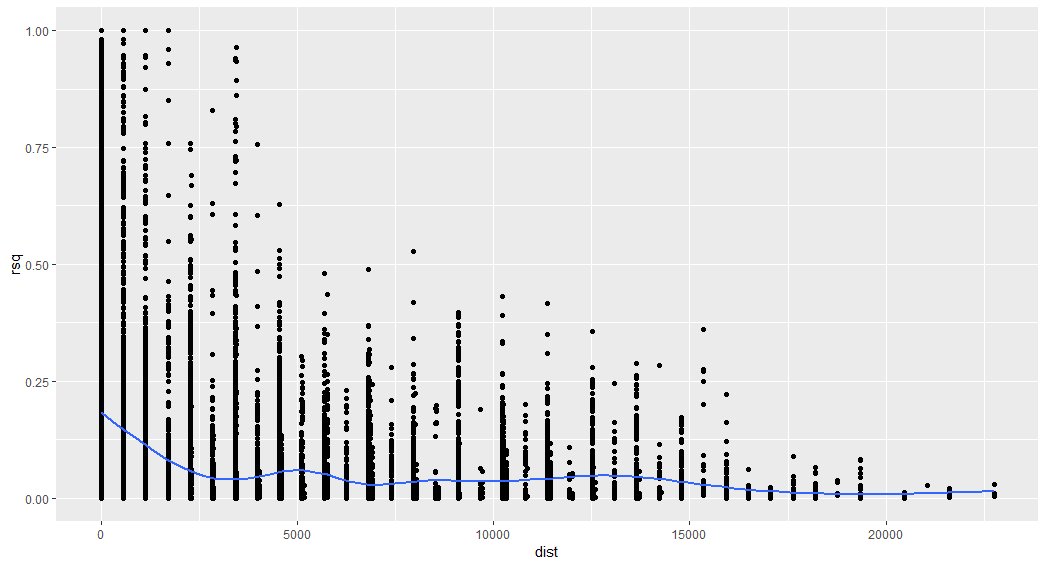

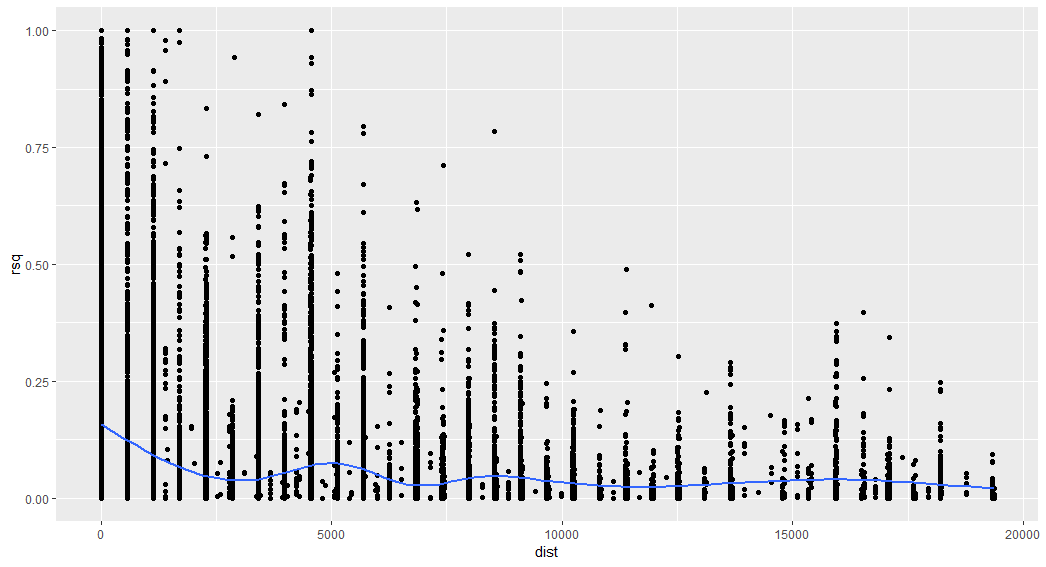


2B

1B


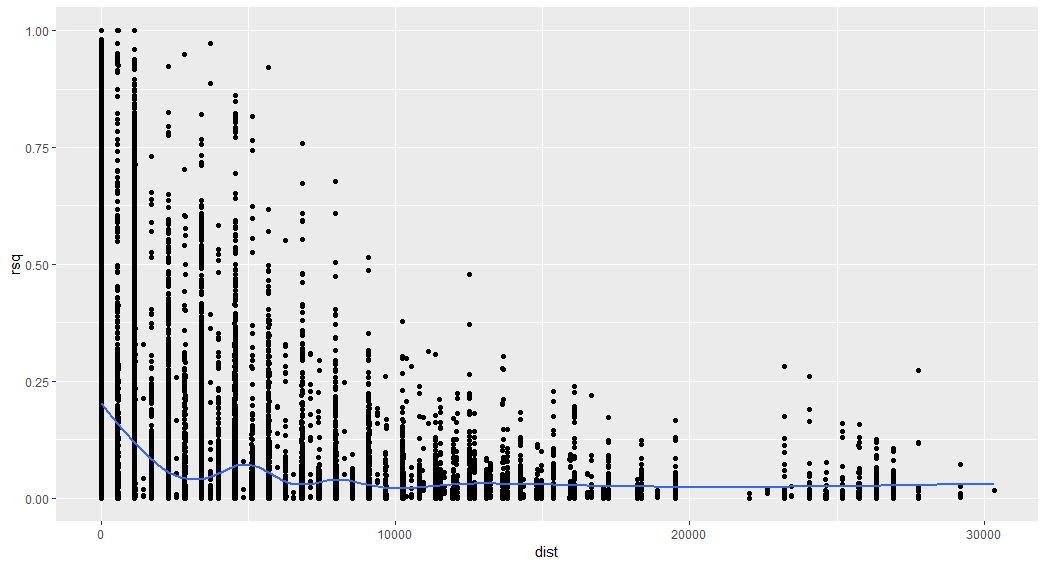

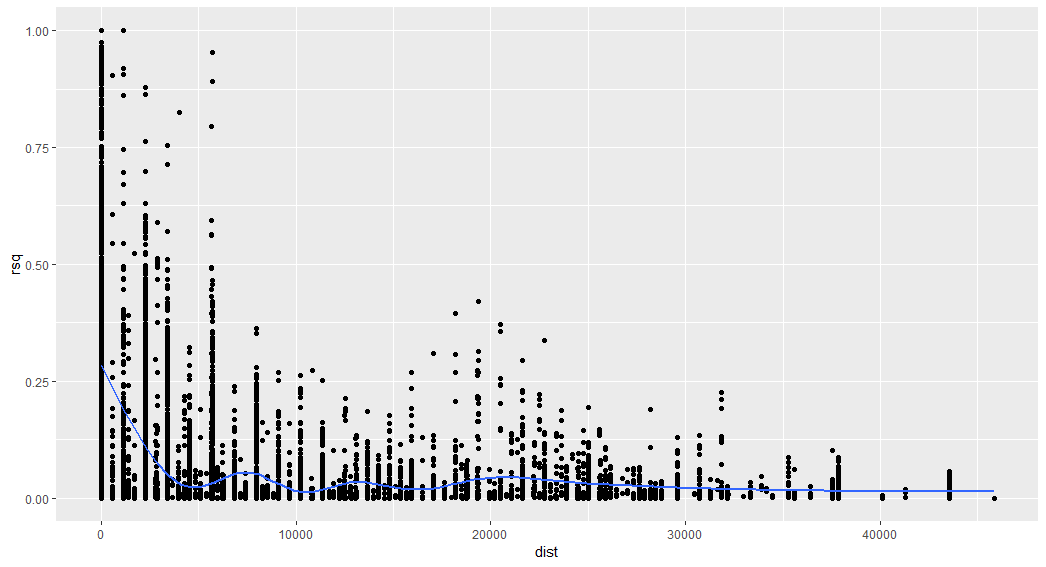


4B

3B


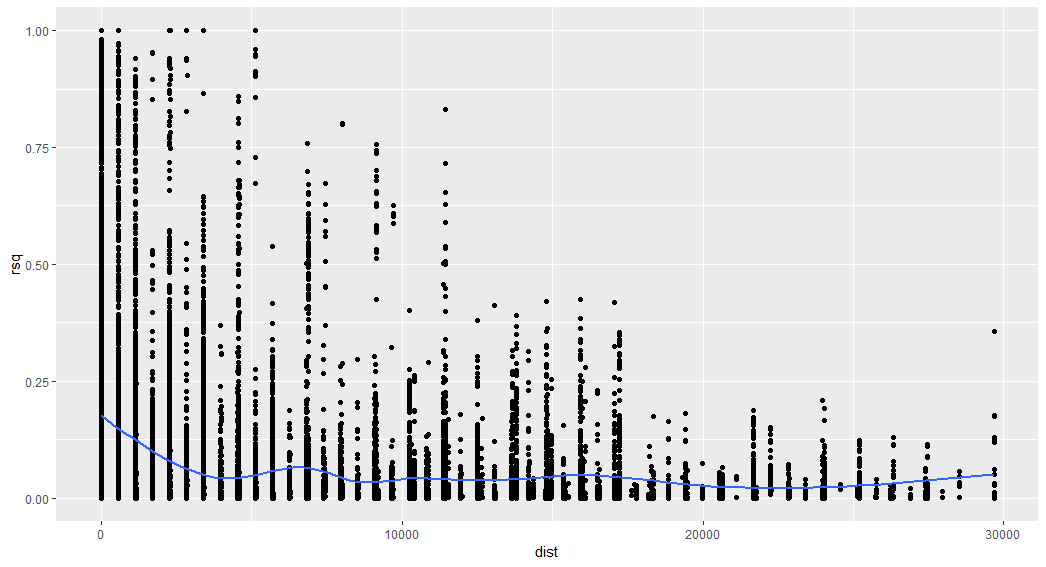

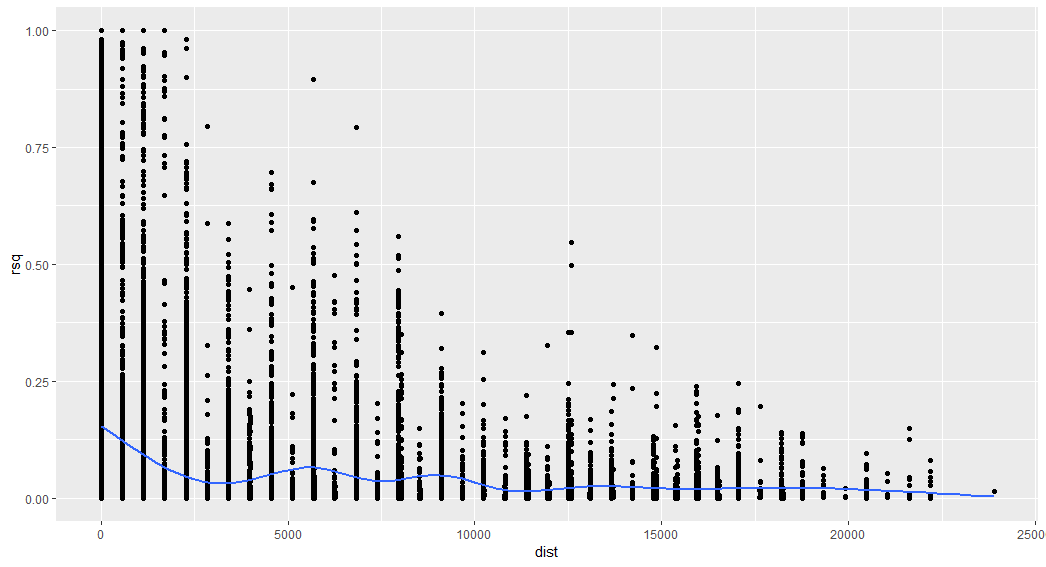


6B

5B


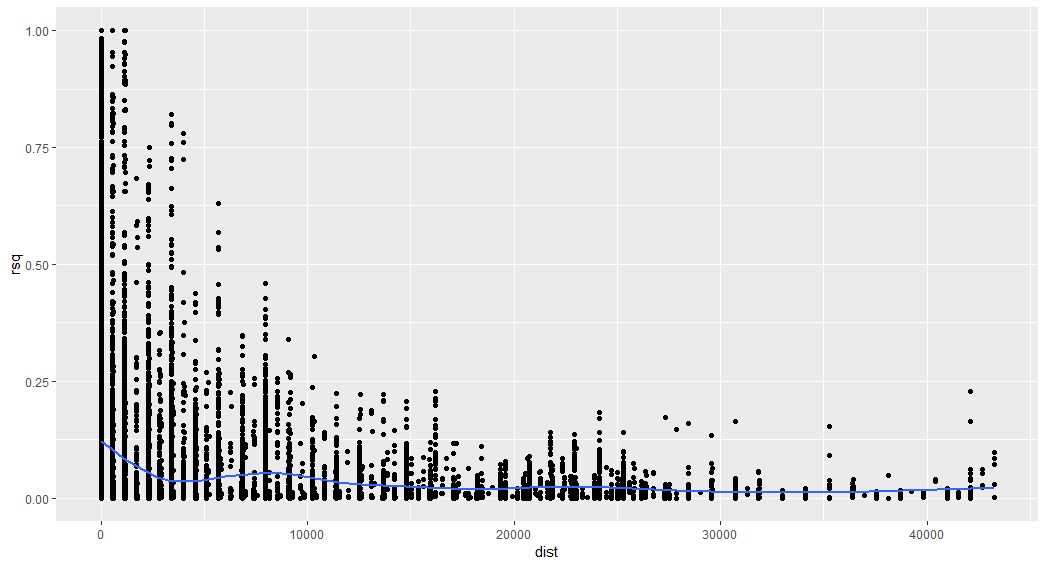


7B

Figure S3. The pattern of LD decay in different chromosomes of genome B in *T. aestivum* based on original SNPs


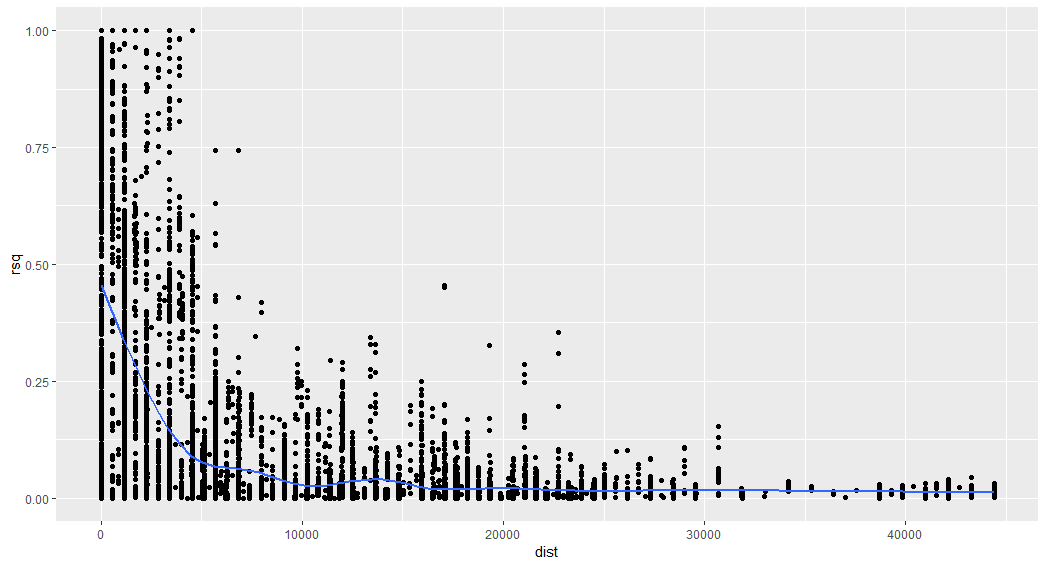

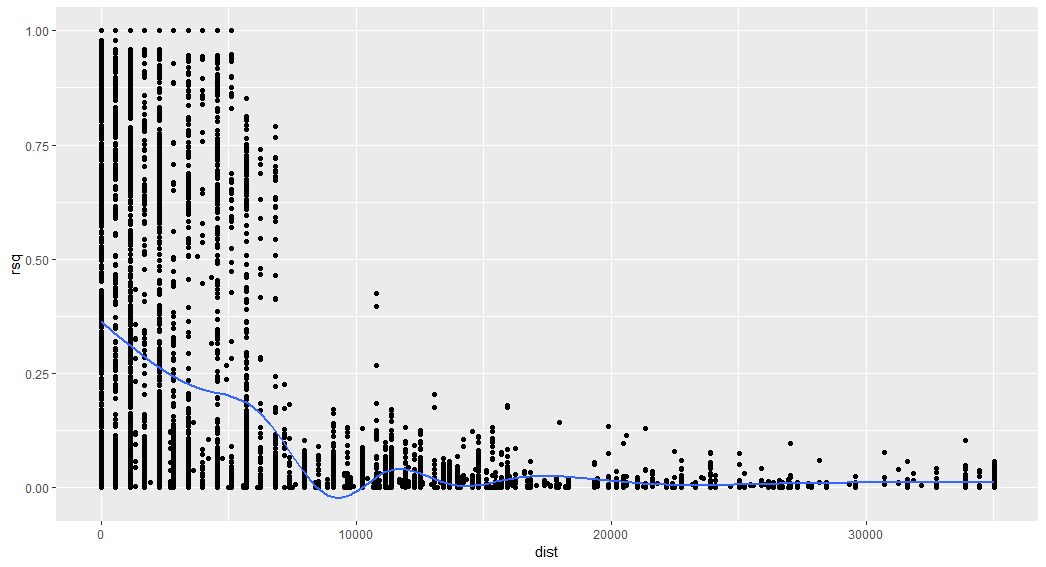


2D

1D


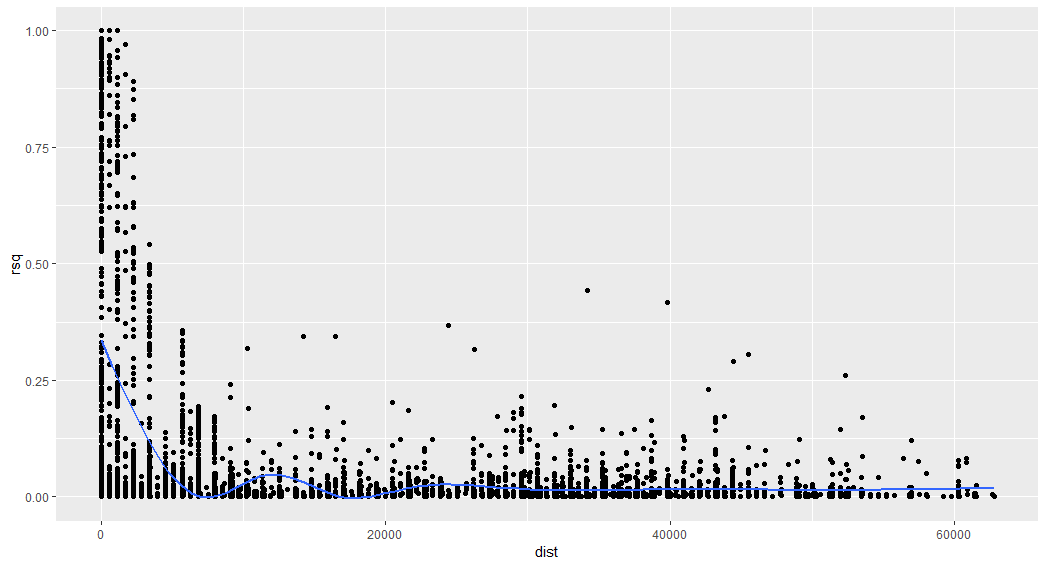

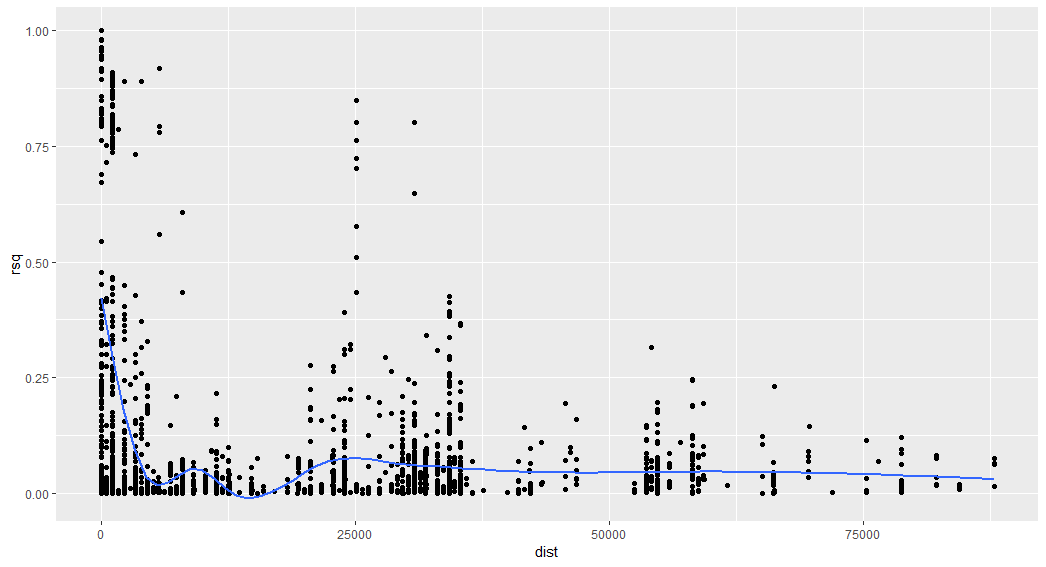


4D

3D


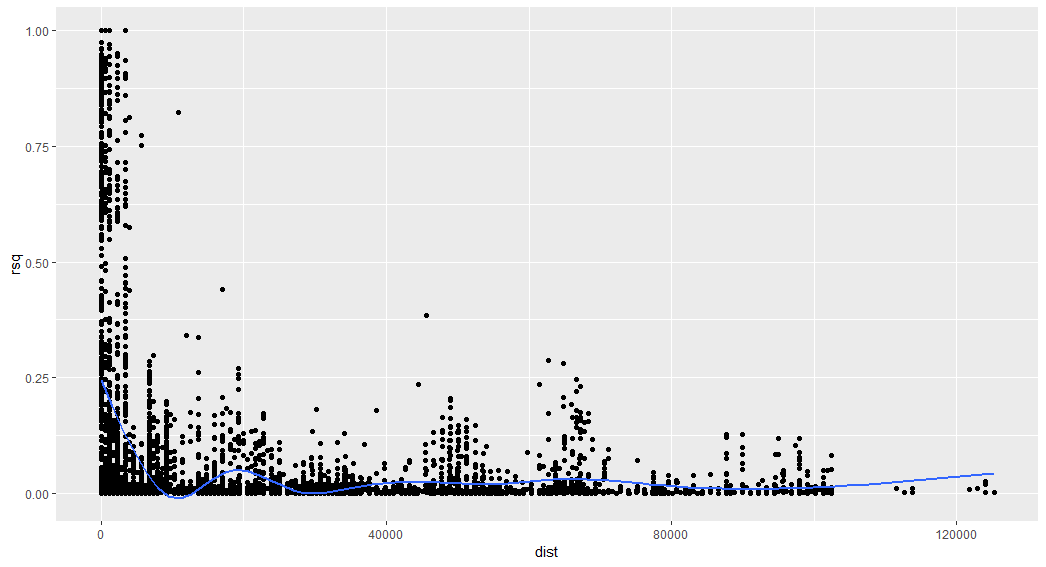

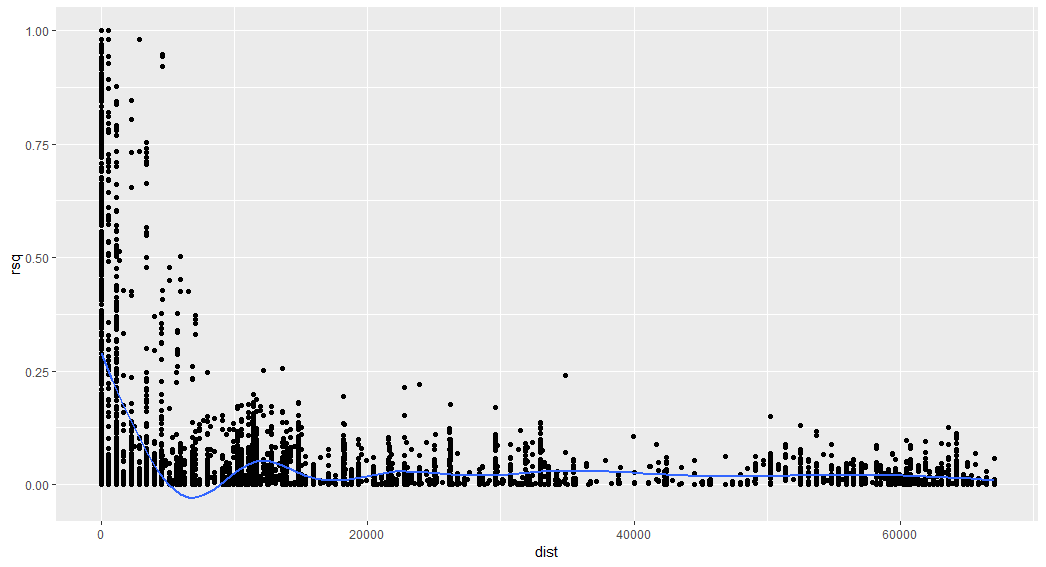


6D

5D


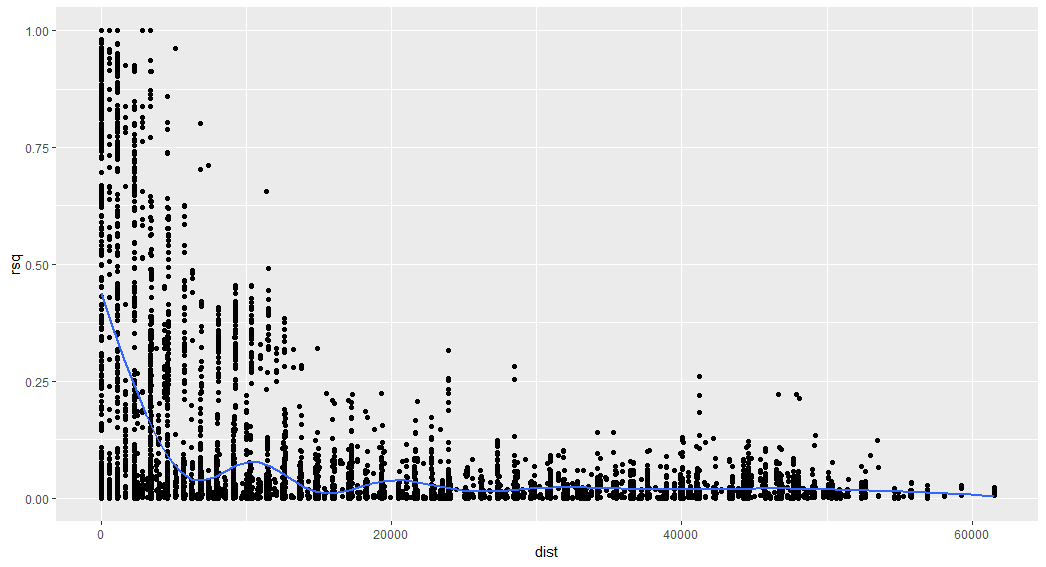


7D

Figure S4. The pattern of LD decay in different chromosomes of genome D in *T. aestivum* based on original SNPs
