# Supplementary material for: Genome-wide association study of agronomic traits in bread wheat reveals novel putative alleles for future breeding programs
Source: BMC Plant Biol. 2019 Dec 5;19:541. doi: 10.1186/s12870-019-2165-4 (PMC6896361; doi:10.1186/s12870-019-2165-4)
Supplement: Supplementary file 4 — Additional file 4 Table S6. Description of expected MTAs using original SNPs for agronomic traits of Iranian wheat accessions exposed to the well-watered condition. Table S7. Description of expected MTAs using original SNPs for agronomic traits of Iranian wheat accessions exposed to the rain-fed condition. Table S8. Description of expected MTAs using imputed SNPs for agronomic traits of Iranian wheat accessions exposed to the well-watered condition. Table S9. Description of expected MTAs using imputed SNPs for agronomic traits of Iranian wheat accessions exposed to the rain-fed condition. [file 12870_2019_2165_MOESM4_ESM.docx]

Table S6

Description of expected MTAs using original SNPs for agronomic traits of Iranian wheat accessions exposed to the well-watered condition

| Row | Marker | sequence | Trait | Chromosome | Position  (bp) | Molecular process | Biological process |
| --- | --- | --- | --- | --- | --- | --- | --- |
| 1 | M37707 | TGCAGCTCGTTATATAACAAGCCAACACTCACTTTGGTAATTCACCCAAGGGTCAAAGAAAGGA_35 | Days to heading and physiological maturity | 1A | [34,425,461-34,427,079](http://ensembl.gramene.org/Triticum_aestivum/Location/View?db=core;g=TraesCS1A02G052400;r=1A:34425461-34427079;t=TraesCS1A02G052400.1;tl=Y4HtqRMAbVzVSdOW-6323-1942647) | ubiquitin-protein transferase activity, | Ubiquitination |
| 2 | M7503 | TGCAGATAAATCAGAAAGGCTGAAATCCCAGATCTGATGCCCTAAGGAAGAATCATCACCGCTA_27 | Emergence | 1A | [152,407,743-152,434,315](http://ensembl.gramene.org/Triticum_aestivum/Location/View?db=core;g=TraesCS1A02G126500;r=1A:152407743-152434315;t=TraesCS1A02G126500.1;tl=Y4HtqRMAbVzVSdOW-6324-1942658) | - | - |
| 3 | M62576 | TGCAGTTACGGATGGCAGTCATCTGGTCCATGAATCATGACAGAGGCACCTGCTCCATAAACAG_47 | Canopy temperature | 1A | [570,131,664-570,140,605](http://ensembl.gramene.org/Triticum_aestivum/Location/View?db=core;g=TraesCS1A02G407600;r=1A:570131664-570140605;t=TraesCS1A02G407600.1;tl=Y4HtqRMAbVzVSdOW-6325-1942662) | oxidoreductase activity, | oxidation-reduction process |
| 4 | M30740 | TGCAGCGCGGACACGGCGGAGCTCTGGAAGCGGAGGTCGATCTTGAAGTCCTGCGCGATCTCCC_39 | Spike length | 1B | [434,409,017-434,411,291](http://ensembl.gramene.org/Triticum_aestivum/Location/View?db=core;g=TraesCS1B02G245000;r=1B:434409017-434411291;t=TraesCS1B02G245000.1;tl=Y4HtqRMAbVzVSdOW-6328-1942908) | DNA binding  protein heterodimerization activity | - |
| 5 | M6102 | TGCAGAGCGCCACGGACGCGGTCAACCCATCTTGAGCGGCCACGCACTGGTCTCTGTGATGTCC_27 | Spike weight and grain yield | 1B | [637,948,814-637,949,773](http://ensembl.gramene.org/Triticum_aestivum/Location/View?db=core;g=TraesCS1B02G412000;r=1B:637948814-637949773;t=TraesCS1B02G412000.1;tl=Y4HtqRMAbVzVSdOW-6332-1943005) | protein binding | - |
| 6 | M6287 | TGCAGAGCTTCTGCGTGCAAAGCAACTTTAGCCTCTCAAGTTTATACTCGTTCGCCGCAACAAG_58 | Leaf greenness | 1B | [637,948,814-637,949,773](http://ensembl.gramene.org/Triticum_aestivum/Location/View?db=core;g=TraesCS1B02G412000;r=1B:637948814-637949773;t=TraesCS1B02G412000.1;tl=Y4HtqRMAbVzVSdOW-6332-1943005) | protein binding | - |
| 7 | M16402 | TGCAGCAGCAGCAGCAGCCTGTATCAATGACGAACATGTATAGGTTAGAGACGGACTGAAGGGA_26 | Spike length | 2A | [204,885,066-204,888,307](http://ensembl.gramene.org/Triticum_aestivum/Location/View?db=core;g=TraesCS2A02G217600;r=2A:204885066-204888307;t=TraesCS2A02G217600.1;tl=Y4HtqRMAbVzVSdOW-6339-1943140) | protein kinase activity  ATP binding | protein phosphorylation |
| 8 | M33027 | TGCAGCGGTGGTCAAGCAGCGTCTGCTCCGCCGAGGCGGTGTGGTTGTCGAAAACGGCGAGCTC_13 | Emergence | 2A | [509,070,654-509,076,530](http://ensembl.gramene.org/Triticum_aestivum/Location/View?db=core;g=TraesCS2A02G295900;r=2A:509070654-509076530;t=TraesCS2A02G295900.1;tl=Y4HtqRMAbVzVSdOW-6342-1943256) | chromatin binding  thiol-dependent ubiquitin-specific protease activity  nucleosome binding  histone binding | histone deubiquitination  protein deubiquitination  negative regulation of transcription, DNA-templated |

Table S6

Continued.

| 9 | M50692 | TGCAGGGCTAAGCACAAGATGTCGTGCATCCTATCATGGGAGAACACCAAGAAGGCGGCGGTCC_32 | Canopy temperature | 2B | [21,949,723-21,954,034](http://ensembl.gramene.org/Triticum_aestivum/Location/View?db=core;g=TraesCS2B02G044900;r=2B:21949723-21954034;tl=Y4HtqRMAbVzVSdOW-6343-1943368) | ADP binding | - |
| --- | --- | --- | --- | --- | --- | --- | --- |
| 10 | M21931 | TGCAGCCAGCCACCACCACCACCACGATCACTGTGTCCGCCACCTTGGCCCAGTTCGAATGTGA_55 | Grain yield | 2B | [23,118,714-23,124,318](http://ensembl.gramene.org/Triticum_aestivum/Location/View?db=core;g=TraesCS2B02G046600;r=2B:23118714-23124318;t=TraesCS2B02G046600.1;tl=F0LHQuzF2iPXXiw8-9077-3601213) | RNA binding  metal ion binding | response to abscisic acid  3'-UTR-mediated mRNA stabilization |
| 11 | M23970 | TGCAGCCCGCAGGTGCATCGGGGACTTACAGTTCAAGCTAAAGATCCACAGAAGCAGAAGAGCT_41 | Grain filling period | 3A | [509,564,225-509,567,338](http://ensembl.gramene.org/Triticum_aestivum/Location/View?db=core;g=TraesCS3A02G280700;r=3A:509564225-509567338;t=TraesCS3A02G280700.1;tl=F0LHQuzF2iPXXiw8-9080-3601761) | calcium ion binding | - |
| 12 | M58206 | TGCAGTCCAGAGCATCCAGCTCGATGACGCCCCAGTTGCTGCCGACGGACACCATCTGCTCGCC_46 | Grain yield, seed number and spike weight | 3B | [25,416,412-25,417,913](http://ensembl.gramene.org/Triticum_aestivum/Location/View?db=core;g=TraesCS3B02G050900;r=3B:25416412-25417913;t=TraesCS3B02G050900.1;tl=F0LHQuzF2iPXXiw8-9084-3604381) | protein binding  zinc ion binding  metal ion binding | ubiquitin-dependent protein catabolic process  multicellular organism development  protein ubiquitination |
| 13 | M58207 | TGCAGTCCAGAGCATCCAGCTCGATGACGCCGCAGTTGCTGCCGACAGACACCATCTGCTCGCC_46 | Grain yield, seed number and spike weight | 3B | [25,416,412-25,417,913](http://ensembl.gramene.org/Triticum_aestivum/Location/View?db=core;g=TraesCS3B02G050900;r=3B:25416412-25417913;t=TraesCS3B02G050900.1;tl=F0LHQuzF2iPXXiw8-9085-3604390) | protein binding  zinc ion binding  metal ion binding | ubiquitin-dependent protein catabolic process  multicellular organism development  protein ubiquitination |
| 14 | M31753 | TGCAGCGGCAGGACGACGCCACTGAGTAGAGATGCAGGTATGACCGCATGACCTTCCTGGGCTT_20 | Anthesis | 3B | [44,856,819-44,857,576](http://ensembl.gramene.org/Triticum_aestivum/Location/View?db=core;g=TraesCS3B02G074100;r=3B:44856819-44857576;t=TraesCS3B02G074100.1;tl=F0LHQuzF2iPXXiw8-9086-3604421) | enzyme inhibitor activity | negative regulation of catalytic activity |
| 15 | M16394 | TGCAGCAGCAGCAGCAGCAGCAAGCGAGAAACGAGCAGAAAGAAGTCAAGCAAAGGCACGAGGA_11 | Leaf greenness | 3D | [13,305,844-13,307,578](http://ensembl.gramene.org/Triticum_aestivum/Location/View?db=core;g=TraesCS3D02G037000;r=3D:13305844-13307578;t=TraesCS3D02G037000.1;tl=F0LHQuzF2iPXXiw8-9089-3604516) | - | abscisic acid biosynthetic process  xanthophyll biosynthetic process |
| 16 | M52526 | TGCAGGTATAACAGCATCATTATCCAACCTACCCCAATGACTCTCGAGCTCAAGCCCCCTCTGG_26 | Grain yield | 3D | [567,197,168-567,200,123](http://ensembl.gramene.org/Triticum_aestivum/Location/View?db=core;g=TraesCS3D02G462900;r=3D:567197168-567200123;t=TraesCS3D02G462900.1;tl=F0LHQuzF2iPXXiw8-9090-3604550) | - | response to UV |

Table S6

Continued.

| 17 | M61323 | TGCAGTGGATCGCGGCACGGAGTTCGCCAAGATGACACCAATGTCAACTTCAGCAAGCCCGCGC_17 | Thousand kernel weight | 4A | [312,669,507-312,708,877](http://ensembl.gramene.org/Triticum_aestivum/Location/View?db=core;g=TraesCS4A02G156500;r=4A:312669507-312708877;t=TraesCS4A02G156500.1;tl=F0LHQuzF2iPXXiw8-9093-3606359) | cation transmembrane transporter activity  calcium:proton antiporter activity | cation transport  calcium ion transport  transmembrane transport |
| --- | --- | --- | --- | --- | --- | --- | --- |
| 18 | M43789 | TGCAGGACTGGAGATCAGCACAGGCCGTCTTTTGCTCCACAACGCCAGGGGCCCAGGAAGAAAG_25 | Grain filling period | 4B | [386,749,723-386,751,143](http://ensembl.gramene.org/Triticum_aestivum/Location/View?db=core;g=TraesCS4B02G176700;r=4B:386749723-386751143;t=TraesCS4B02G176700.1;tl=F0LHQuzF2iPXXiw8-9094-3606565) | DNA binding  DNA-binding transcription factor activity  sequence-specific DNA binding | regulation of transcription, DNA-templated |
| 19 | M736 | TGCAGAAAGGTACCACTCATTCGTACATCACTCCAACTGATGTATGAAGGTTGTTCATGGCGAC_18 | Spike weight | 4B | [481,233,765-481,237,258](http://ensembl.gramene.org/Triticum_aestivum/Location/View?db=core;g=TraesCS4B02G230100;r=4B:481233765-481237258;t=TraesCS4B02G230100.1;tl=F0LHQuzF2iPXXiw8-9097-3606676) | hydrolase activity | phosphatidylinositol dephosphorylation |
| 20 | M2830 | TGCAGAATGCTACTAAATGCTTCTCTGACATGTTGTCTCCGTCCGTCTGTGAGTTGACGGACGC_38 | Spike weight | 4B | [516,951,473-516,957,806](http://ensembl.gramene.org/Triticum_aestivum/Location/View?db=core;g=TraesCS4B02G250400;r=4B:516951473-516957806;t=TraesCS4B02G250400.1;tl=F0LHQuzF2iPXXiw8-9098-3606706) | hydrolase activity | cytokinin biosynthetic process |
| 21 | M48893 | TGCAGGCTCCGCTAAACCCTAGACTTGACGGCGAGGGTGCGTCGGGTGGGGAAAGGGGGAGAAA_11 | Seed number | 4D | [318,493,437-318,496,592](http://ensembl.gramene.org/Triticum_aestivum/Location/View?db=core;g=TraesCS4D02G182300;r=4D:318493437-318496592;tl=F0LHQuzF2iPXXiw8-9099-3606775) | GTPase activity | - |
| 22 | M43176 | TGCAGGACACAATCCCCACGAGTGTGCTGCCGCTCTGCCGCAGCGTGTATGCGGGCGCGTGGTT_17 | Grain filling period | 5A | [705,842,223-705,847,913](http://ensembl.gramene.org/Triticum_aestivum/Location/View?db=core;g=TraesCS5A02G553300;r=5A:705842223-705847913;tl=15nu9HkaLidxQ7xp-9395-3661686) | protein binding  methyltransferase activity | DNA methylation |
| 23 | M46084 | TGCAGGCACGATGGAGCCGCTCGCGTTGTCGCCGAGATCGGAAGAGCGGGATCACCGACTGCCC_8 | Leaf greenness | 5B | [408,972,574-408,974,941](http://ensembl.gramene.org/Triticum_aestivum/Location/View?db=core;g=TraesCS5B02G231900;r=5B:408972574-408974941;t=TraesCS5B02G231900.1;tl=15nu9HkaLidxQ7xp-9398-3661781) | - | male meiotic nuclear division  female meiotic nuclear division |
| 24 | M4772 | TGCAGACTCACACACAAGCTGCTACAACTAAGCGCTGGGCAGATACATCCACCCGAGATCGGAA_44 | Thousand kernel weight | 5B | [435,156,029-435,159,077](http://ensembl.gramene.org/Triticum_aestivum/Location/View?db=core;g=TraesCS5B02G252500;r=5B:435156029-435159077;t=TraesCS5B02G252500.1;tl=15nu9HkaLidxQ7xp-9399-3661790) | protein kinase activity  ATP binding | protein phosphorylation |

Table S6

Continued.

| 25 | M51975 | TGCAGGGTTCAGCTGAACTGCGTGCAGGCATGCCAAATTTTCTCGGCTGATCTAGTGAAATCTT_25 | Grain yield | 6A | [499,196,996-499,206,172](http://ensembl.gramene.org/Triticum_aestivum/Location/View?db=core;g=TraesCS6A02G272400;r=6A:499196996-499206172;t=TraesCS6A02G272400.1;tl=15nu9HkaLidxQ7xp-9402-3661916) | DNA binding | DNA replication |
| --- | --- | --- | --- | --- | --- | --- | --- |
| 26 | M54066 | TGCAGGTGCTCACGGGGCTGCTCGACCTCAATATCGTCGAGCAGGTACAGGAGGCGCTCAGGAG_34 | Spike length | 6A | [571,844,755-571,849,919](http://ensembl.gramene.org/Triticum_aestivum/Location/View?db=core;g=TraesCS6A02G338200;r=6A:571844755-571849919;tl=15nu9HkaLidxQ7xp-9403-3661932) | protein kinase activity  ATP binding | protein phosphorylation |
| 27 | M6597 | TGCAGAGGCACGTGAACAGCTCCTTCTTGGACATGGAGCACTCGCGGCAGTCATCGTCGTCGGC_9 | Seed number | 6A | [601,230,996-601,233,757](http://ensembl.gramene.org/Triticum_aestivum/Location/View?db=core;g=TraesCS6A02G381900;r=6A:601230996-601233757;t=TraesCS6A02G381900.1;tl=15nu9HkaLidxQ7xp-9404-3661945) | protein binding | - |
| 28 | M18648 | TGCAGCAGTGGAGTCCGTCGTCCATCTGGCTGGACCCTTGCAGGCGGTAAACCCACACCACCCA_51 | Grain yield and spike weight | 6B | [569,820,759-569,828,281](http://ensembl.gramene.org/Triticum_aestivum/Location/View?db=core;g=TraesCS6B02G321600;r=6B:569820759-569828281;t=TraesCS6B02G321600.1;tl=15nu9HkaLidxQ7xp-9407-3662165) | - | malate transport |
| 29 | M46504 | TGCAGGCATATGCTCGCCCCACATGTTCGTAGACAGGCTATCCTGCCGTTACGCATTGTGGTAC_30 | Grain filling period | 6B | [534,921,073-534,927,092](http://ensembl.gramene.org/Triticum_aestivum/Location/View?db=core;g=TraesCS6B02G298000;r=6B:534921073-534927092;tl=15nu9HkaLidxQ7xp-9409-3662202) | guanyl-nucleotide exchange factor activity  Rho guanyl-nucleotide exchange factor activity | - |
| 30 | M29539 | TGCAGCGCACGCGCGGACGCTGGCCCCAACATGGCTGCTCGGCGGTGGCCGAGATCGGAAGAGC_30 | Grain filling period | 7A | [540,771,636-540,775,107](http://ensembl.gramene.org/Triticum_aestivum/Location/View?db=core;g=TraesCS7A02G367300;r=7A:540771636-540775107;t=TraesCS7A02G367300.1;tl=15nu9HkaLidxQ7xp-9414-3662232) | protein binding | - |
| 31 | M10316 | TGCAGATTGGGCTTGAGGAAATCTAACAAAACTTGGTGGATCGGCAAAGCCTGGATGAAATTCA_6 | Seed number | 7B | [675,187,632-675,190,824](http://ensembl.gramene.org/Triticum_aestivum/Location/View?db=core;g=TraesCS7B02G406400;r=7B:675187632-675190824;t=TraesCS7B02G406400.1;tl=Ydg12nXF6ASREgu3-9461-3667905) | DNA binding | - |
| 32 | M49191 | TGCAGGCTGGGCTTGAGGAAATCTAACAAAACTTGGTGGATCGGCAAAGCCTGGATGAAATTCA_6 | Seed number | 7B | [675,187,632-675,190,824](http://ensembl.gramene.org/Triticum_aestivum/Location/View?db=core;g=TraesCS7B02G406400;r=7B:675187632-675190824;t=TraesCS7B02G406400.1;tl=Ydg12nXF6ASREgu3-9462-3667916) | DNA binding | - |

Table S7

Description of expected MTAs using original SNPs for agronomic traits of Iranian wheat accessions exposed to the rain-fed condition

| Row | Marker | sequence | Trait | Chromosome | Position  (bp) | Molecular process | Biological process |
| --- | --- | --- | --- | --- | --- | --- | --- |
| 1 | M12246 | TGCAGCAAGCCAACAACTGCATTGTTTCCTATGATCCATTGAGAGGCATAGACACGCTTTGATT_12 | Days to heading | 1A | [522,189,569-522,191,557](http://ensembl.gramene.org/Triticum_aestivum/Location/View?db=core;g=TraesCS1A02G334800;r=1A:522189569-522191557;t=TraesCS1A02G334800.1;tl=dmRfdrx5yQhR63IW-18993-9011662) | monooxygenase activity  iron ion binding  oxidoreductase activity  oxidoreductase activity, acting on paired donors, with incorporation or reduction of molecular oxygen  heme binding  metal ion binding  (Cytochrome P450 superfamily) | oxidation-reduction process |
| 2 | M60456 | TGCAGTGCACGGTCGGCTACGCAACCACCACCGCCGCTGCTGCTGCTGCTACTCCCGCGGCGCG_32 | Grain filling period | 1A | [11,811,881-11,815,829](http://ensembl.gramene.org/Triticum_aestivum/Location/View?db=core;g=TraesCS1A02G024100;r=1A:11811881-11815829;t=TraesCS1A02G024100.1;tl=dmRfdrx5yQhR63IW-18996-8957729) | hydrolase activity, hydrolyzing O-glycosyl compounds  beta-galactosidase activity  hydrolase activity  hydrolase activity, acting on glycosyl bonds  carbohydrate binding | carbohydrate metabolic process  metabolic process |
| 3 | M63808 | TGCAGTTGAAGTCGCGGTGGATGACGGCGGGGGAGGTGTGCTCGTGCAGAAACTCCAGCGCGCG_49 | Spike and straw weight | 1B | [457,750,965-457,756,510](http://ensembl.gramene.org/Triticum_aestivum/Location/View?db=core;g=TraesCS1B02G260300;r=1B:457750965-457756510;t=TraesCS1B02G260300.1;tl=dmRfdrx5yQhR63IW-19003-8961228) | protein kinase activity  ATP binding | protein phosphorylation |
| 4 | M35345 | TGCAGCTATACATGCGGCGACGCAGAAAGTACCACCGAGATCGGAAGAGCGGGATCACCGACTG_14 | Anthesis | 1B | [637,815,266-637,817,819](http://ensembl.gramene.org/Triticum_aestivum/Location/View?db=core;g=TraesCS1B02G411600;r=1B:637815266-637817819;t=TraesCS1B02G411600.1;tl=dmRfdrx5yQhR63IW-19006-8962249) | protein binding  (F-box domain) | - |
| 5 | M2237 | TGCAGAAGGGGACGCCTCGGAATCTACGGCAGAGGACCGCCTCAGCGGCCTTCCCGACGGCGTC_30 | Spike weight and spike length | 1B | [26,855,662-26,857,170](http://ensembl.gramene.org/Triticum_aestivum/Location/View?db=core;g=TraesCS1B02G047100;r=1B:26855662-26857170;t=TraesCS1B02G047100.1;tl=dmRfdrx5yQhR63IW-19008-8962925) | protein binding  (F-box domain) | - |

Table S7

Continued.

| 6 | M40663 | TGCAGCTTCACCAGCGACTTGAGGGCGCGGAGAGCCTTCCTGGCCTGCCGAGATCGGAAGAGCG_27 | Spike length | 1D | [432,807,534-432,809,474](http://ensembl.gramene.org/Triticum_aestivum/Location/View?db=core;g=TraesCS1D02G344200;r=1D:432807534-432809474;t=TraesCS1D02G344200.1;tl=dmRfdrx5yQhR63IW-19011-8963294) | protein binding  (IQ motif, EF-hand binding site) | - |
| --- | --- | --- | --- | --- | --- | --- | --- |
| 7 | M33741 | TGCAGCGTGCCTGTGGCTATACGTACTGATCGTTTCCCCGTGTTCCTCCACACGGGCAGGTTCG_18 | Straw weight | 2A | [125,251,161-125,253,229](http://ensembl.gramene.org/Triticum_aestivum/Location/View?db=core;g=TraesCS2A02G170200;r=2A:125251161-125253229;tl=dmRfdrx5yQhR63IW-19013-8963305) | strictosidine synthase activity  (key enzyme in alkaloid biosynthesis) | biosynthetic process |
| 8 | M28880 | TGCAGCGAGGAGATGGCGGTATCCGTGGCTGCCGCGCCGAGATCGGAAGAGCGGGATCACCGAC_20 | Anthesis | 2A | [682,488,148-682,490,689](http://ensembl.gramene.org/Triticum_aestivum/Location/View?db=core;g=TraesCS2A02G429100;r=2A:682488148-682490689;tl=dmRfdrx5yQhR63IW-19015-8963384) | structural constituent of ribosome  (Ribosomal protein L22/L17 superfamily) | translation |
| 9 | M64783 | TGCAGTTTCAATCTGATTGATTATTTCCTTTGCTCGCGATGGATGGATGCAGGTGACAGGGACC_23 | Thousand kernel weight | 2A | [714,030,047-714,031,310](http://ensembl.gramene.org/Triticum_aestivum/Location/View?db=core;g=TraesCS2A02G472500;r=2A:714030047-714031310;t=TraesCS2A02G472500.1;tl=dmRfdrx5yQhR63IW-19017-8987892) | protein binding  (BTB/POZ domain) | - |
| 10 | M26577 | TGCAGCCTCCAATCGTGTACACACCTCCGTAAACAGATCTCGATTCTTCACTCCCTGTAGAGAG_5 | Thousand kernel weight | 2B | [134,240,300-134,249,722](http://ensembl.gramene.org/Triticum_aestivum/Location/View?db=core;g=TraesCS2B02G160800;r=2B:134240300-134249722;t=TraesCS2B02G160800.1;tl=dmRfdrx5yQhR63IW-19021-8992421) | protein binding  (Armadillo)  Involved in  membrane | - |
| 11 | M15903 | TGCAGCAGAGAATAATAGATGGAGGGAGGGGTGGTGCAAGTATAGCACCCGAGATCGGAAGAGC_41 | Spike weight | 2B | [47,175,539-47,181,332](http://ensembl.gramene.org/Triticum_aestivum/Location/View?db=core;g=TraesCS2B02G084300;r=2B:47175539-47181332;t=TraesCS2B02G084300.1;tl=dmRfdrx5yQhR63IW-19022-8993969) | ADP binding  (NB-ARC) | - |
| 12 | M42282 | TGCAGGAACCAAACACCCGCAGAGCATCATCAGGAACAGCAACCCAAGGAGACTAACACTAGCA_42 | Grain yield | 2B | [140,843,932-140,847,330](http://ensembl.gramene.org/Triticum_aestivum/Location/View?db=core;g=TraesCS2B02G167900;r=2B:140843932-140847330;tl=ffxTCE8YCmj1TJXz-19194-9215879) | DNA-binding transcription factor activity  (INVOLVED IN Basic-leucine zipper domain) | regulation of transcription, DNA-templated  response to endoplasmic reticulum stress |
| 13 | M39019 | TGCAGCTGCGCGGCCGTGATCCCGACGAGGCCCATACCCTCCGCGAGGCCGCGCCCGCGGCCTC_10 | Seed number | 3A | [542,734,930-542,736,059](http://ensembl.gramene.org/Triticum_aestivum/Location/View?db=core;g=TraesCS3A02G306200;r=3A:542734930-542736059;t=TraesCS3A02G306200.1;tl=ffxTCE8YCmj1TJXz-19203-9216152) | sequence-specific DNA binding  (Transcription factor TGA like domain) | transcription, DNA-templated |

Table S7

Continued.

| 14 | M53129 | TGCAGGTCCTCGTCCCCAGCGGCGACTATGCTGTTGCTGGCGGGGAGTACGAAGTCGACCTTGA_24 | Days to heading | 3A | [737,114,170-737,114,739](http://ensembl.gramene.org/Triticum_aestivum/Location/View?db=core;g=TraesCS3A02G519100;r=3A:737114170-737114739;t=TraesCS3A02G519100.1;tl=ffxTCE8YCmj1TJXz-19208-9216227) | rRNA N-glycosylase activity  (Ribosome-inactivating protein) | negative regulation of translation |
| --- | --- | --- | --- | --- | --- | --- | --- |
| 15 | M23813 | TGCAGCCCGAACCCGCGCTCGTAGTACCCGAGGTAGGGGACGCCGTCGGTGCAGTGGACCTGGA_40 | Grain filling period | 3B | [24,911,564-24,944,677](http://ensembl.gramene.org/Triticum_aestivum/Location/View?db=core;g=TraesCS3B02G049100;r=3B:24911564-24944677;tl=ffxTCE8YCmj1TJXz-19210-9216467) | nucleotide binding  protein kinase activity  protein serine/threonine kinase activity  ATP binding  polysaccharide binding | protein phosphorylation |
| 16 | M46075 | TGCAGGCACGACCGCATGACCTTCTCGAACTTGGCGTCCTTGGCATGGGCGAGCGCAGACTCGA_25 | Peduncle length | 3B | [44,856,819-44,857,576](http://ensembl.gramene.org/Triticum_aestivum/Location/View?db=core;g=TraesCS3B02G074100;r=3B:44856819-44857576;t=TraesCS3B02G074100.1;tl=ffxTCE8YCmj1TJXz-19211-9216536) | enzyme inhibitor activity  (Pectinesterase inhibitor domain) | negative regulation of catalytic activity |
| 17 | M63923 | TGCAGTTGAGTGTGTCGAGTAAAGCGAAGGCTGAAAGGACGACATTCTGATTGGAATCAAATGC_33 | Plant height | 3B | [756,822,502-756,824,850](http://ensembl.gramene.org/Triticum_aestivum/Location/View?db=core;g=TraesCS3B02G514300;r=3B:756822502-756824850;t=TraesCS3B02G514300.1;tl=ffxTCE8YCmj1TJXz-19215-9216799) | methyltransferase activity  (SAM dependent carboxyl methyltransferase) | - |
| 18 | M13731 | TGCAGCACAGGGATCATGAACGGCTTGGACTCGTTAGTGAACTCGCCGAGATCGGAAGAGCGGG_35 | Days to heading | 3B | [770,952,582-770,953,739](http://ensembl.gramene.org/Triticum_aestivum/Location/View?db=core;g=TraesCS3B02G529100;r=3B:770952582-770953739;t=TraesCS3B02G529100.1;tl=ffxTCE8YCmj1TJXz-19216-9216833) | hydrolase activity, hydrolyzing O-glycosyl compounds  (Glycoside hydrolase family 17) | carbohydrate metabolic process |
| 19 | M61706 | TGCAGTGGGTCGTCGGAGCATCCAATCAGATCTCCACTACACGAACGAGACTAGCAGCAAGAGG_43 | Thousand kernel weight | 3B | [783,413,489-783,414,580](http://ensembl.gramene.org/Triticum_aestivum/Location/View?db=core;g=TraesCS3B02G547700;r=3B:783413489-783414580;tl=ffxTCE8YCmj1TJXz-19217-9216847) | GTPase activity  GTP binding  (Small GTPase AND Small GTP-binding protein domain) | - |
| 20 | M11229 | TGCAGCAACACATCCAAGGCACAGCCACTCCTCTCCCCAACACCTCTCCTCCTCCGCGTGAGCT_20 | Canopy temperature | 3B | [511,034,602-511,051,546](http://ensembl.gramene.org/Triticum_aestivum/Location/View?db=core;g=TraesCS3B02G317300;r=3B:511034602-511051546;t=TraesCS3B02G317300.1;tl=ffxTCE8YCmj1TJXz-19219-9230380) | serine-type endopeptidase activity  hydrolase activity  (Peptidase S8/S53 domain AND serine-type endopeptidase activity) | proteolysis |

Table S7

Continued.

| 21 | M425 | TGCAGAAACATACTCGACTCTTTTGAGCAGGATGCAGCGCGATACGGCTGCCGAGATCGGAAGA_11 | Plant height | 4A |  | nucleic acid binding  RNA binding | - |
| --- | --- | --- | --- | --- | --- | --- | --- |
| 22 | M39416 | TGCAGCTGGACCCTGGAAACGCCGAGATGGAGAAAGTGTTCATGTAAGCTATTTACCAAGAATG_54 | Grain filling period | 4B | [17,491,425-17,493,654](http://ensembl.gramene.org/Triticum_aestivum/Location/View?db=core;g=TraesCS4B02G024700;r=4B:17491425-17493654;t=TraesCS4B02G024700.1;tl=IhXtRfMOytOfnC96-19274-9242596) | protein binding  (Tetratricopeptide-like helical domain superfamily) | - |
| 23 | M30688 | TGCAGCGCGCGCTCCAGGTTGCCGTCCCTCACCTGCACCACGATCCCCCGCGCCGCCTGCGTCT_56 | Spike weight | 4B | [24,855,498-24,858,072](http://ensembl.gramene.org/Triticum_aestivum/Location/View?db=core;g=TraesCS4B02G033900;r=4B:24855498-24858072;t=TraesCS4B02G033900.1;tl=IhXtRfMOytOfnC96-19275-9242617) | structural constituent of ribosome  (Ribosomal protein S21) | translation |
| 24 | M50807 | TGCAGGGCTTCGTGATCTCAGATTGGATGGGCATTGACAAGCTCACCACCCCTTATGGGGCGGA_46 | Leaf greenness | 5A | [585,396,508-585,399,414](http://ensembl.gramene.org/Triticum_aestivum/Location/View?db=core;g=TraesCS5A02G389100;r=5A:585396508-585399414;t=TraesCS5A02G389100.1;tl=IhXtRfMOytOfnC96-19281-9242711) | hydrolase activity, hydrolyzing O-glycosyl compounds  hydrolase activity  (Glycoside hydrolase family 3 C-terminal domain superfamily) | carbohydrate metabolic process |
| 25 | M22451 | TGCAGCCATGAGGCCGAGACACTCGGCGGTGAGAGGCTTGAAGATGCGCTGCTCATCGGAGTCG_26 | Grain yield | 5A | [692,371,595-692,373,001](http://ensembl.gramene.org/Triticum_aestivum/Location/View?db=core;g=TraesCS5A02G535900;r=5A:692371595-692373001;t=TraesCS5A02G535900.1;tl=IhXtRfMOytOfnC96-19283-9242760) | transferase activity, transferring acyl groups other than amino-acyl groups | - |
| 26 | M64789 | TGCAGTTTCACAGTCCCAGCAGCAATGAGAAATGCGAAGGAATATACTGCACGCCAAAGATGCA_14 | Spike weight | 5A | [698,512,064-698,517,752](http://ensembl.gramene.org/Triticum_aestivum/Location/View?db=core;g=TraesCS5A02G542700;r=5A:698512064-698517752;t=TraesCS5A02G542700.1;tl=IhXtRfMOytOfnC96-19285-9242785) | protein kinase activity  ubiquitin-protein transferase activity  ATP binding | protein phosphorylation  protein ubiquitination |
| 27 | M2395 | TGCAGAAGTTGAAAAGTAGACCAAGCAAGCTATCTCACACTCCATACCTGTTCGATTGAAGCCA_39 | Days to physiological maturity | 5B | [34,717,367-34,722,525](http://ensembl.gramene.org/Triticum_aestivum/Location/View?db=core;g=TraesCS5B02G031600;r=5B:34717367-34722525;t=TraesCS5B02G031600.1;tl=IhXtRfMOytOfnC96-19289-9242798) | ADP binding | - |
| 28 | M31995 | TGCAGCGGCGACGGCATGAGCGACCTCTGGCCCGTCGGCTTCGGCGGCGGCGTCGCCATGGGCT_18 | Plant height | 5B | [289,256,291-289,259,208](http://ensembl.gramene.org/Triticum_aestivum/Location/View?db=core;g=TraesCS5B02G156800;r=5B:289256291-289259208;t=TraesCS5B02G156800.1;tl=IhXtRfMOytOfnC96-19292-9242841) | nucleic acid binding  zinc ion binding  (Zinc finger, CCHC-type) | - |

Table S7

Continued.

| 29 | M17000 | TGCAGCAGCCTGAAAACAGTGCTCCCATCAGATACAGAAACTGGCAACTCTAACACAAAGATGT_47 | Peduncle length | 5B | [509,184,617-509,187,519](http://ensembl.gramene.org/Triticum_aestivum/Location/View?db=core;g=TraesCS5B02G325200;r=5B:509184617-509187519;tl=IhXtRfMOytOfnC96-19296-9242964) | - | proteasome assembly |
| --- | --- | --- | --- | --- | --- | --- | --- |
| 30 | M25701 | TGCAGCCGCTCTTCGGCGGCTCTTGCATCGATGAGGTCGCGGGTGGGGCTGATGCGGAAAAGTG_19 | Peduncle length | 5B | [513,711,454-513,713,217](http://ensembl.gramene.org/Triticum_aestivum/Location/View?db=core;g=TraesCS5B02G329700;r=5B:513711454-513713217;t=TraesCS5B02G329700.1;tl=w9MYUKV7u7EbZSzs-19351-9245543) | D-arabinono-1,4-lactone oxidase activity  oxidoreductase activity  flavin adenine dinucleotide binding  FAD binding | oxidation-reduction process |
| 31 | M1295 | TGCAGAACCATACGAAGAAAATGCATGTGAGCAGCAGGACGGCTGCTGACACCGAGATCGGAAG_12 | Grain filling period | 5B | [532,931,865-532,935,285](http://ensembl.gramene.org/Triticum_aestivum/Location/View?db=core;g=TraesCS5B02G353400;r=5B:532931865-532935285;t=TraesCS5B02G353400.1;tl=w9MYUKV7u7EbZSzs-19352-9245573) | nucleotide binding  protein kinase activity  protein serine/threonine kinase activity  ATP binding  kinase activity  transferase activity | protein phosphorylation  phosphorylation  recognition of pollen |
| 32 | M25700 | TGCAGCCGCTCTTCGGCGGCTCTTGCATCGATGAGCTCGCGGGTGCGGGTAAGGGGCAAGTCGT_35 | Plant height | 5B | [513,646,921-513,649,139](http://ensembl.gramene.org/Triticum_aestivum/Location/View?db=core;g=TraesCS5B02G329600;r=5B:513646921-513649139;t=TraesCS5B02G329600.1;tl=w9MYUKV7u7EbZSzs-19353-9245583) | catalytic activity  D-arabinono-1,4-lactone oxidase activity  oxidoreductase activity  flavin adenine dinucleotide binding  FAD binding | oxidation-reduction process |
| 33 | M13078 | TGCAGCAATGACTCATATCAGCAGAAAACAATGATCAAGTTAGCCATGTACTACATGCAATGTG_14 | Grain filling period | 5B | [545,940,819-545,944,476](http://ensembl.gramene.org/Triticum_aestivum/Location/View?db=core;g=TraesCS5B02G367400;r=5B:545940819-545944476;t=TraesCS5B02G367400.1;tl=w9MYUKV7u7EbZSzs-19354-9245611) | serine-type endopeptidase activity  peptidase activity  serine-type peptidase activity  hydrolase activity | proteolysis |
| 34 | M33549 | TGCAGCGTCGTTCGTTGATCTTTTTGGTATGCCGATCCAAGCAGAGGGTACCCCAAGGAAGCTT_49 | Leaf greenness | 5B | [545,940,819-545,944,476](http://ensembl.gramene.org/Triticum_aestivum/Location/View?db=core;g=TraesCS5B02G367400;r=5B:545940819-545944476;t=TraesCS5B02G367400.1;tl=w9MYUKV7u7EbZSzs-19355-9245634) | serine-type endopeptidase activity  peptidase activity  serine-type peptidase activity  hydrolase activity | proteolysis |

Table S7

Continued.

| 35 | M2933 | TGCAGAATTCAGATAGTAATGTCAGACTCGACGCGGCATTCGTTATCCCACTAGAACGAGGTTC_32 | Straw weight | 5B | [549,673,160-549,675,414](http://ensembl.gramene.org/Triticum_aestivum/Location/View?db=core;g=TraesCS5B02G370700;r=5B:549673160-549675414;t=TraesCS5B02G370700.1;tl=w9MYUKV7u7EbZSzs-19356-9245646) | nucleic acid binding  RNA binding | - |
| --- | --- | --- | --- | --- | --- | --- | --- |
| 36 | M57846 | TGCAGTCAGAGATGATCAAGTTAAGGTCGTCGAACCCGTCATGGCAGCCGCCGCCGAGATCGGA_17 | Seed number | 5B | [637,387,009-637,389,605](http://ensembl.gramene.org/Triticum_aestivum/Location/View?db=core;g=TraesCS5B02G461900;r=5B:637387009-637389605;tl=w9MYUKV7u7EbZSzs-19357-9245651) | protein binding  (BTB/POZ domain) | - |
| 37 | M63646 | TGCAGTTCTGAACTCTCAACAGTAGCCACAGATGTTTATAAACCTCTTGGTGTCAATCATCGAC_11 | Plant height | 5D | [429,731,223-429,733,516](http://ensembl.gramene.org/Triticum_aestivum/Location/View?db=core;g=TraesCS5D02G341600;r=5D:429731223-429733516;t=TraesCS5D02G341600.1;tl=w9MYUKV7u7EbZSzs-19359-9245696) | protein binding | - |
| 38 | M1727 | TGCAGAAGAAGGCTGGCGGAGGCGGTAGTCGAGCTGGCGCTGGTGACCAATGGCGATGCCCTGG_26 | Grain filling period | 5D | [505,822,665-505,824,306](http://ensembl.gramene.org/Triticum_aestivum/Location/View?db=core;g=TraesCS5D02G462000;r=5D:505822665-505824306;t=TraesCS5D02G462000.1;tl=w9MYUKV7u7EbZSzs-19361-9245748) | nucleic acid binding  (Zinc finger C2H2-type) | - |
| 39 | M64051 | TGCAGTTGCCAAAGTATGTGGCAGGGAGGGGCGGCTTCAACCGTGCCCGAGATCGGAAGAGCGG_34 | Anthesis | 5D | [560,186,102-560,187,499](http://ensembl.gramene.org/Triticum_aestivum/Location/View?db=core;g=TraesCS5D02G558200;r=5D:560186102-560187499;t=TraesCS5D02G558200.1;tl=w9MYUKV7u7EbZSzs-19364-9245808) | transferase activity, transferring acyl groups other than amino-acyl groups | - |
| 40 | M38998 | TGCAGCTGCGCCTTAACGCTGCTGTGATTTGCGACCCAAATCTGCACATCATTCAAGCCCGCAT_8 | Peduncle length | 6A | [558,980,931-558,984,101](http://ensembl.gramene.org/Triticum_aestivum/Location/View?db=core;g=TraesCS6A02G325300;r=6A:558980931-558984101;t=TraesCS6A02G325300.1;tl=w9MYUKV7u7EbZSzs-19365-9245827) | hydrolase activity, hydrolyzing O-glycosyl compounds  (Glycoside hydrolase family 17) | carbohydrate metabolic process |
| 41 | M48658 | TGCAGGCGTTGTCCTCCTGCCGCCGCCTCGACCACGCCCGCCACGTCGCGCCAAGGCCCACGGC_50 | Leaf greenness | 6B | [40,449,889-40,453,113](http://ensembl.gramene.org/Triticum_aestivum/Location/View?db=core;g=TraesCS6B02G061000;r=6B:40449889-40453113;t=TraesCS6B02G061000.1;tl=w9MYUKV7u7EbZSzs-19370-9245992) | protein kinase activity  protein binding  ATP binding | protein phosphorylation |
| 42 | M46504 | TGCAGGCATATGCTCGCCCCACATGTTCGTAGACAGGCTATCCTGCCGTTACGCATTGTGGTAC_30 | Grain filling period | 6B | [534,921,073-534,927,092](http://ensembl.gramene.org/Triticum_aestivum/Location/View?db=core;g=TraesCS6B02G298000;r=6B:534921073-534927092;tl=w9MYUKV7u7EbZSzs-19371-9246012) | guanyl-nucleotide exchange factor activity  Rho guanyl-nucleotide exchange factor activity  (PRONE domain AND | - |
| 43 | M30520 | TGCAGCGCGACCCCTCTGCTGGCGAGCTGGGTTGGCCCATATATGTCTGCTTATTTTATAAAAA_57 | Days to emergence | 6B | [532,043,561-532,045,921](http://ensembl.gramene.org/Triticum_aestivum/Location/View?db=core;g=TraesCS6B02G296200;r=6B:532043561-532045921;t=TraesCS6B02G296200.1;tl=w9MYUKV7u7EbZSzs-19372-9246020) | protein binding  anaphase-promoting complex binding  ubiquitin-protein transferase activator activity | positive regulation of ubiquitin protein ligase activity |

Table S7

Continued.

| 44 | M51526 | TGCAGGGTACGTGAGTGATTAAACTGGCTGAGTTAATTGTGATCGGCATTTGATGGTTATGGCC_47 | Grain yield and spike weight | 6B | [664,500,180-664,501,715](http://ensembl.gramene.org/Triticum_aestivum/Location/View?db=core;g=TraesCS6B02G390100;r=6B:664500180-664501715;tl=w9MYUKV7u7EbZSzs-19375-9246065) | - | asymmetric cell division |
| --- | --- | --- | --- | --- | --- | --- | --- |
| 45 | M64814 | TGCAGTTTCAGTCAAACTCTGTTTCTGACAAACTGAAGGTAATAAATACTACAGTAGCACGAAT_48 | Grain filling period | 6D | [467,187,916-467,194,390](http://ensembl.gramene.org/Triticum_aestivum/Location/View?db=core;g=TraesCS6D02G393300;r=6D:467187916-467194390;t=TraesCS6D02G393300.1;tl=1tLJXvXStOe0KdDG-19534-9257962) | protein binding  (Leucine-rich repeat) | - |
| 46 | M45739 | TGCAGGATTTGGGTGCCACCACATGACGTGACCAAGACCTTGATTGACAACTTTGAGAAGAAGA_46 | Days to emergence | 7A | [30,137,182-30,141,326](http://ensembl.gramene.org/Triticum_aestivum/Location/View?db=core;g=TraesCS7A02G061300;r=7A:30137182-30141326;t=TraesCS7A02G061300.1;tl=1tLJXvXStOe0KdDG-19535-9258068) | protein binding  (Tetratricopeptide-like helical domain superfamily) | - |
| 47 | M21028 | TGCAGCCACAGCGACGTAGCCGACGGCGGCCAGTCCCTGGACGTCACGCTCTTCGACCACCGTG_17 | Grain yield | 7A | [41,047,488-41,048,806](http://ensembl.gramene.org/Triticum_aestivum/Location/View?db=core;g=TraesCS7A02G075300;r=7A:41047488-41048806;t=TraesCS7A02G075300.1;tl=1tLJXvXStOe0KdDG-19536-9258095) | protein binding  (F-box domain) | - |
| 48 | M56337 | TGCAGTACCGCTCTTCCCGAGCTGGCACTACTGTTCCACCCGTCCAACGATCTGTTGGGGCATC_32 | Grain yield | 7A | [80,142,837-80,144,941](http://ensembl.gramene.org/Triticum_aestivum/Location/View?db=core;g=TraesCS7A02G124400;r=7A:80142837-80144941;t=TraesCS7A02G124400.1;tl=1tLJXvXStOe0KdDG-19539-9258133) | galactoside 2-alpha-L-fucosyltransferase activity  (Xyloglucan fucosyltransferase) | fucosylation  cell wall biogenesis |
| 49 | M24471 | TGCAGCCCTGAGATGCCCTTCTCCCTCCCCGTGAGCCTCCTGGCAGCAGGAACGAATGCCCGAG_44 | Thousand kernel weight | 7A | [603,023,754-603,026,488](http://ensembl.gramene.org/Triticum_aestivum/Location/View?db=core;g=TraesCS7A02G412100;r=7A:603023754-603026488;t=TraesCS7A02G412100.1;tl=1tLJXvXStOe0KdDG-19542-9259544) | transmembrane transporter activity | transmembrane transport |
| 50 | M43138 | TGCAGGACAAGACATAGGAACCAAACCTCCTAAGTCAGCAGCAGAAATGGCCCGATCGACAGCT_47 | Grain filling period | 7B | [86,806,273-86,807,807](http://ensembl.gramene.org/Triticum_aestivum/Location/View?db=core;g=TraesCS7B02G077100;r=7B:86806273-86807807;t=TraesCS7B02G077100.1;tl=1tLJXvXStOe0KdDG-19549-9259661) | cyclin-dependent protein serine/threonine kinase inhibitor activity | cell cycle arrest  negative regulation of cyclin-dependent protein serine/threonine kinase activity |

Table S7

Continued.

| 51 | M59840 | TGCAGTGAAAGGTCTGGTAGACTGGCGTGTGTGCTACATGTGATGTGCGTGTGCTTCGTCAGTA_60 | Spike weight | 7B | [621,396,199-621,404,428](http://ensembl.gramene.org/Triticum_aestivum/Location/View?db=core;g=TraesCS7B02G359300;r=7B:621396199-621404428;t=TraesCS7B02G359300.1;tl=1tLJXvXStOe0KdDG-19550-9260101) | Membrane transport protein | auxin-activated signaling pathway  transmembrane transport |
| --- | --- | --- | --- | --- | --- | --- | --- |
| 52 | M48015 | TGCAGGCGCCCGTTGGCGAGTAATGCTAGACGTACAGGTTTCTAGGGGGGTTTACAGGCAGCTT_10 | Leaf greenness | 7B | [483,531,381-483,541,977](http://ensembl.gramene.org/Triticum_aestivum/Location/View?db=core;g=TraesCS7B02G262500;r=7B:483531381-483541977;t=TraesCS7B02G262500.1;tl=1tLJXvXStOe0KdDG-19552-9259734) | protein kinase activity  ATP binding  (Serine/threonine-protein kinase, active site AND Protein kinase domain) | protein phosphorylation |
| 53 | M51059 | TGCAGGGGCAGGTGGACAAGGCCACGGCGCACAGCGCCGCCGACAGGGAGGCGGCCGAGACGAG_37 | Spike length | 7B | [435,787,250-435,791,035](http://ensembl.gramene.org/Triticum_aestivum/Location/View?db=core;g=TraesCS7B02G232400;r=7B:435787250-435791035;t=TraesCS7B02G232400.1;tl=1tLJXvXStOe0KdDG-19554-9259838) | zinc ion binding  oxidoreductase activity  metal ion binding  (Alcohol dehydrogenase, zinc-type, conserved site, Polyketide synthase, enoylreductase domain) | oxidation-reduction process |
| 54 | M53016 | TGCAGGTCCCATGGCCTCTACCATAGTCGAACGGAGGTGGATGCGCTTTGAGGTGGATGCCTGA_35 | Grain filling period | 7B | [15,713,548-15,714,633](http://ensembl.gramene.org/Triticum_aestivum/Location/View?db=core;g=TraesCS7B02G018700;r=7B:15713548-15714633;t=TraesCS7B02G018700.1;tl=1tLJXvXStOe0KdDG-19555-9259908) | DNA binding  DNA-binding transcription factor activity  (NA-binding domain superfamily, AP2/ERF domain) | regulation of transcription, DNA-templated |

Table S8

Description of expected MTAs using imputed SNPs for agronomic traits of Iranian wheat accessions exposed to the well-watered condition

| ROW | Marker | sequence | Trait | Chromosome | Position  (bp) | Molecular process | Biological process |
| --- | --- | --- | --- | --- | --- | --- | --- |
| 1 | M36032 | TGCAGCTCATCACTAGTCTCGCGCTCGGGCAGCAGGACCGAGCTCGTCTCGCGCCCG_25 | Grain yield and spike weight | 1A | [206,792,054-206,805,538](http://ensembl.gramene.org/Triticum_aestivum/Location/View?db=core;g=TraesCS1A02G135100;r=1A:206792054-206805538;tl=N3avDUbC3riguCS7-15246-7677851) | nucleotide binding  DNA binding  damaged DNA binding  ATP binding  mismatched DNA binding | DNA repair  pyrimidine dimer repair  cellular response to DNA damage stimulus  negative regulation of reciprocal meiotic recombination |
| 2 | M37707 | TGCAGCTCGTTATATAACAAGCCAACACTCACTTTGGTAATTCACCCAAGGGTCAAAGAAAGGA_35 | Physiological maturity | 1A | [34,425,461-34,427,079](http://ensembl.gramene.org/Triticum_aestivum/Location/View?db=core;g=TraesCS1A02G052400;r=1A:34425461-34427079;t=TraesCS1A02G052400.1;tl=U5LwI1W2R0J4s6kV-9504-3675530) | ubiquitin-protein transferase activity | protein ubiquitination |
| 3 | M38766 | TGCAGCTGCCATCCATCGCCCCGTCATGGCTGTCGTGGCAGCCGCCGCACCAAGCGGCCTTCGC_25 | Grain filling period | 1A | [155,068,692-155,077,135](http://ensembl.gramene.org/Triticum_aestivum/Location/View?db=core;g=TraesCS1A02G127600;r=1A:155068692-155077135;tl=U5LwI1W2R0J4s6kV-9515-3727734) | nucleic acid binding  DNA binding  ATP-dependent DNA helicase activity  ATP binding  ATP-dependent helicase activity  hydrolase activity, acting on acid anhydrides, in phosphorus-containing anhydrides | nucleobase-containing compound metabolic process |

Table S8

Continued.

| 4 | M34075 | TGCAGCGTTCGACCAGCTCATCACCCGCTTCCGAGATCGGAAGAGCGGGATCACCGACTGCCCA_19 | Leaf greenness | 1A | [60,954,701-60,956,424](http://ensembl.gramene.org/Triticum_aestivum/Location/View?db=core;g=TraesCS1A02G078100;r=1A:60954701-60956424;t=TraesCS1A02G078100.1;tl=U5LwI1W2R0J4s6kV-9530-3780577) | peroxidase activity  oxidoreductase activity  heme binding  metal ion binding | response to oxidative stress  hydrogen peroxide catabolic process  oxidation-reduction process  cellular oxidant detoxification |
| --- | --- | --- | --- | --- | --- | --- | --- |
| 5 | M10506 | TGCAGATTTGATTAGCTTGCTTGGATGTACTCCAACTATTAGATACACAGTATGATCACAGTAC_37 | Grain filling period | 1A | [471,503,708-471,507,782](http://ensembl.gramene.org/Triticum_aestivum/Location/View?db=core;g=TraesCS1A02G276500;r=1A:471503708-471507782;t=TraesCS1A02G276500.1;tl=FNJaxdSPEg4yibly-10506-4050939) | GTPase activity  GTP binding | - |
| 6 | M36962 | TGCAGCTCCTCGTCGGTGGTGTCGCGGGTGAGGCTCTTCTGCCGTCAGCGTGGACCGAGATCGG_45 | Spike length | 4A | [629,034,391-629,106,268](http://ensembl.gramene.org/Triticum_aestivum/Location/View?db=core;g=TraesCS4A02G354700;r=4A:629034391-629106268;t=TraesCS4A02G354700.1;tl=FNJaxdSPEg4yibly-10509-4054002) | ATP binding  ATPase activity | - |
| 7 | M64294 | TGCAGTTGGCAGCCAGCTAGTTGGCGCAGTCGGTAGCCG_32 | Spike length | 4A | [722,287,491-722,294,625](http://ensembl.gramene.org/Triticum_aestivum/Location/View?db=core;g=TraesCS4A02G456900;r=4A:722287491-722294625;t=TraesCS4A02G456900.1;tl=FNJaxdSPEg4yibly-10512-4078631) | protein kinase activity  ATP binding | protein phosphorylation |
| 8 | M29072 | TGCAGCGATCACAAAAAGTGAAAGGTTGCTAGCTGCAAGCTTGCATCAGCTAGCACAGTTAGGA_28 | Grain filling period | 4A | [595,046,187-595,056,137](http://ensembl.gramene.org/Triticum_aestivum/Location/View?db=core;g=TraesCS4A02G292200;r=4A:595046187-595056137;tl=FNJaxdSPEg4yibly-10513-4079280) | DNA binding  sequence-specific DNA binding | regulation of transcription, DNA-templated |
| 9 | M8354 | TGCAGATCCCGTGTCAACTACTACACTGTCTCACATCACGTCTGGGAATGACCAGTGATTTTCA_35 | Leaf greenness | 4A | [738,980,242-738,983,792](http://ensembl.gramene.org/Triticum_aestivum/Location/View?db=core;g=TraesCS4A02G485100;r=4A:738980242-738983792;t=TraesCS4A02G485100.1;tl=FNJaxdSPEg4yibly-10518-4083898) | iron ion binding  oxidoreductase activity, acting on paired donors, with incorporation or reduction of molecular oxygen  heme binding | oxidation-reduction process |

Table S8

Continued.

| 10 | M22355 | TGCAGCCATCCAGACAAATAAGTCCAGTTGAAGTGCAGAACATGGAGGCCAATCGCACACTGGA_57 | Leaf greenness | 4B | [643,020,569-643,025,121](http://ensembl.gramene.org/Triticum_aestivum/Location/View?db=core;g=TraesCS4B02G350300;r=4B:643020569-643025121;t=TraesCS4B02G350300.1;tl=FNJaxdSPEg4yibly-10520-4083939) | protein kinase activity  ubiquitin-protein transferase activity  ATP binding | protein phosphorylation  protein ubiquitination |
| --- | --- | --- | --- | --- | --- | --- | --- |
| 11 | M1239 | TGCAGAACATGCCCTTCATCCATGCAATAAGCACATAACAATAAAAATTTTCTTTTGAGAATCA_46 | Emergence | 4B | [285,530,769-285,580,442](http://ensembl.gramene.org/Triticum_aestivum/Location/View?db=core;g=TraesCS4B02G156000;r=4B:285530769-285580442;t=TraesCS4B02G156000.1;tl=FNJaxdSPEg4yibly-10523-4084017) | protein binding  ubiquitin protein ligase activity | protein ubiquitination |
| 12 | M20572 | TGCAGCCAAAGCTCGATAGGCCCCTCGGCGGGATCTCGAAGGCGAAGGAATGAGGAAGGTGAAT_42 | Grain filling period | 4B | [168,159,714-168,192,894](http://ensembl.gramene.org/Triticum_aestivum/Location/View?db=core;g=TraesCS4B02G128600;r=4B:168159714-168192894;t=TraesCS4B02G128600.1;tl=FNJaxdSPEg4yibly-10527-4104188) | drug transmembrane transporter activity  antiporter activity | drug transmembrane transport  transmembrane transport |
| 13 | M33754 | TGCAGCGTGCGCCGCTTTTTCCCTGGCCTTCTTGGCCCTGTTGTGCCTGTCGGTGGTGACGGCC_59 | Spike length | 4B | [386,744,409-386,747,753](http://ensembl.gramene.org/Triticum_aestivum/Location/View?db=core;g=TraesCS4B02G176500;r=4B:386744409-386747753;tl=FNJaxdSPEg4yibly-10534-4169344)  [386,746,396-386,748,710](http://ensembl.gramene.org/Triticum_aestivum/Location/View?db=core;g=TraesCS4B02G176600;r=4B:386746396-386748710;t=TraesCS4B02G176600.1;tl=FNJaxdSPEg4yibly-10534-4169344) | catalytic activity  tRNA-specific adenosine deaminase activity  zinc ion binding  hydrolase activity  lipoyl(octanoyl) transferase activity | tRNA modification  cellular protein modification process  protein lipoylation |
| 14 | M34314 | TGCAGCTAACTAGCCTGAGATAATGCCAGCAACTCTGCTCGGTAGCTTTCTTAAGAAGGCCTTA_45 | Spike length | 4B | [386,744,409-386,747,753](http://ensembl.gramene.org/Triticum_aestivum/Location/View?db=core;g=TraesCS4B02G176500;r=4B:386744409-386747753;tl=4BMTbfkITbweclIQ-14737-7312133) | catalytic activity tRNA-specific adenosine deaminase activity  zinc ion binding  hydrolase activity | tRNA modification |

Table S8

Continued.

| 15 | M36277 | TGCAGCTCCACGAGACATTTTCGTGCTTCGTGATGATGAAGAAGAAGAACTGCCTCTGAAGGAG_10 | Grain filling period | 4B | [34,051,499-34,055,077](http://ensembl.gramene.org/Triticum_aestivum/Location/View?db=core;g=TraesCS4B02G047000;r=4B:34051499-34055077;t=TraesCS4B02G047000.1;tl=EUFtPztZxIDNhutm-13061-5572521) | protein binding  methyltransferase activity | DNA methylation |
| --- | --- | --- | --- | --- | --- | --- | --- |
| 16 | M43789 | TGCAGGACTGGAGATCAGCACAGGCCGTCTTTTGCTCCACAACGCCAGGGGCCCAGGAAGAAAG_25 | Grain filling period | 4B | [386,749,723-386,751,143](http://ensembl.gramene.org/Triticum_aestivum/Location/View?db=core;g=TraesCS4B02G176700;r=4B:386749723-386751143;t=TraesCS4B02G176700.1;tl=EUFtPztZxIDNhutm-13068-5580267) | DNA binding  DNA-binding transcription factor activity  sequence-specific DNA binding | regulation of transcription, DNA-templated |
| 17 | M6507 | TGCAGAGGAGTATTCATACTAATACTAGCTAAATGTTTTACGGAGTAATCAGTTACAAATTTTG_33 | Grain filling period | 4B | [386,201,155-386,205,040](http://ensembl.gramene.org/Triticum_aestivum/Location/View?db=core;g=TraesCS4B02G175800;r=4B:386201155-386205040;t=TraesCS4B02G175800.1;tl=EUFtPztZxIDNhutm-13073-5580616) | DNA-binding transcription factor activity | regulation of transcription, DNA-templated |
| 18 | M6508 | TGCAGAGGAGTATTCTTACTAATACTAGCTAAACGTTTTACGGAGTAATCAGTTACAAATTTTG_33 | Grain filling period | 4B | [386,201,155-386,205,040](http://ensembl.gramene.org/Triticum_aestivum/Location/View?db=core;g=TraesCS4B02G175800;r=4B:386201155-386205040;t=TraesCS4B02G175800.1;tl=EUFtPztZxIDNhutm-13074-5580628) | DNA-binding transcription factor activity | regulation of transcription, DNA-templated |
| 19 | M8246 | TGCAGATCCAGAGGTTAGCTATTCCTTGCAGCTTATATCATGGAACAGGACACCAAGTCATTGC_46 | Grain filling period | 4B | [207,093,023-207,104,105](http://ensembl.gramene.org/Triticum_aestivum/Location/View?db=core;g=TraesCS4B02G145600;r=4B:207093023-207104105;tl=EUFtPztZxIDNhutm-13075-5580640) | nucleic acid binding  RNA binding | mRNA processing |
| 20 | M48126 | TGCAGGCGCGGTAGGCGGACGCGAGTGCGACCAGGCGGAGGAGGAGGCCGAGATCGGAAGAGCG_27 | Spike length | 4B | [33,624,155-33,639,107](http://ensembl.gramene.org/Triticum_aestivum/Location/View?db=core;g=TraesCS4B02G046600;r=4B:33624155-33639107;tl=EUFtPztZxIDNhutm-13077-5580856) | nucleotide binding  protein kinase activity  protein serine/threonine kinase activity  ATP binding  kinase activity  transferase activity | protein phosphorylation  phosphorylation |

Table S8

Continued.

| 21 | M736 | TGCAGAAAGGTACCACTCATTCGTACATCACTCCAACTGATGTATGAAGGTTGTTCATGGCGAC_18 | Spike weight | 4B | [481,233,765-481,237,258](http://ensembl.gramene.org/Triticum_aestivum/Location/View?db=core;g=TraesCS4B02G230100;r=4B:481233765-481237258;t=TraesCS4B02G230100.1;tl=EUFtPztZxIDNhutm-13078-5580867) | hydrolase activity | phosphatidylinositol dephosphorylation |
| --- | --- | --- | --- | --- | --- | --- | --- |
| 22 | M38045 | TGCAGCTCTGGAATTTGAGAACATGGGTGATACTATCCAACTTCTGGACGAGAATCCGAGATCG_21 | Leaf greenness | 4B | [262,707,612-262,727,077](http://ensembl.gramene.org/Triticum_aestivum/Location/View?db=core;g=TraesCS4B02G153400;r=4B:262707612-262727077;t=TraesCS4B02G153400.1;tl=EUFtPztZxIDNhutm-13083-5589854) | - | regulation of proteasomal ubiquitin-dependent protein catabolic process |
| 23 | M52153 | TGCAGGTAAAGAAGATGAAAGAGCTAAGCGGCGGCTATAACATTGTTGGTCTGTCGCAGGTATA_11 | Spike length | 4B | [648,498,795-648,502,912](http://ensembl.gramene.org/Triticum_aestivum/Location/View?db=core;g=TraesCS4B02G358000;r=4B:648498795-648502912;t=TraesCS4B02G358000.1;tl=EUFtPztZxIDNhutm-13085-5589892) | palmitoyl hydrolase activity | macromolecule depalmitoylation |
| 24 | M40819 | TGCAGCTTCCATTTCATTCCTTCCTGCGCCATGGGTAACAAAAATTCAACTTCTTCAGTTAACA_32 | Spike length | 4B | [667,563,369-667,564,460](http://ensembl.gramene.org/Triticum_aestivum/Location/View?db=core;g=TraesCS4B02G392200;r=4B:667563369-667564460;t=TraesCS4B02G392200.1;tl=EUFtPztZxIDNhutm-13086-5589912) | protein binding | - |
| 25 | M52712 | TGCAGGTCACAACACCTCCATCTCTGAATCGAATTATTAGTGTGTTGCAATGCAACAAGGAAGG_30 | Spike weight | 4D | [3,396,041-3,400,090](http://ensembl.gramene.org/Triticum_aestivum/Location/View?db=core;g=TraesCS4D02G006100;r=4D:3396041-3400090;t=TraesCS4D02G006100.1;tl=EUFtPztZxIDNhutm-13088-5589987) | beta-amylase activity  hydrolase activity  hydrolase activity, acting on glycosyl bonds  amylopectin maltohydrolase activity | polysaccharide catabolic process  carbohydrate metabolic process  metabolic process |
| 26 | M42980 | TGCAGGAATGATTTAACTTTTAACAGAGTAGCTATTGTTAATTTCTTGCAGGTTATCTTCAGGG_23 | Emergence | 5A | [471,686,053-471,695,176](http://ensembl.gramene.org/Triticum_aestivum/Location/View?db=core;g=TraesCS5A02G255900;r=5A:471686053-471695176;t=TraesCS5A02G255900.1;tl=AR78Pl1sbj1Aaksc-15024-7491281) | serine-type endopeptidase activity  peptidase activity  hydrolase activity | proteolysis |

Table S8

Continued.

| 27 | M57386 | TGCAGTATCGCAAGAGTAAAATGAAGTAGACAAAAACCTTGTATCATTAAAAGAGGCAGTCACC_18 | Emergence | 5A | [467,397,067-467,403,109](http://ensembl.gramene.org/Triticum_aestivum/Location/View?db=core;g=TraesCS5A02G251700;r=5A:467397067-467403109;t=TraesCS5A02G251700.1;tl=AR78Pl1sbj1Aaksc-15029-7492115) | serine-type endopeptidase activity  serine-type peptidase activity  serine-type exopeptidase activity | proteolysis |
| --- | --- | --- | --- | --- | --- | --- | --- |
| 28 | M1047 | TGCAGAAATTTTCATGTAATCGTGCTCTAGGCACTCTCTTATTTCTATGGGAATTATATGTTGT_7 | Spike length | 5A | [589,583,883-589,585,788](http://ensembl.gramene.org/Triticum_aestivum/Location/View?db=core;g=TraesCS5A02G394500;r=5A:589583883-589585788;t=TraesCS5A02G394500.1;tl=AR78Pl1sbj1Aaksc-15032-7497468) | transferase activity  transferase activity, transferring hexosyl groups | - |
| 29 | M49504 | TGCAGGGAAGTTGATATATTCGAATAGCCGTGTCCCTCGGTAAAAGGATGACACGGAGTTGTAC_12 | Grain filling period | 5A | [130,088,479-130,098,402](http://ensembl.gramene.org/Triticum_aestivum/Location/View?db=core;g=TraesCS5A02G093700;r=5A:130088479-130098402;t=TraesCS5A02G093700.1;tl=AR78Pl1sbj1Aaksc-15034-7581261) | catalytic activity  transferase activity, transferring phosphorus-containing groups  mannose-ethanolamine phosphotransferase activity | GPI anchor biosynthetic process |
| 30 | M36886 | TGCAGCTCCTCCAGCTCCGCATCGGCCTCCGCCTCTAACTCGATGAAATGCCGAGATCGGAAGA_20 | Days to heading | 5B | [96,755,686-96,758,071](http://ensembl.gramene.org/Triticum_aestivum/Location/View?db=core;g=TraesCS5B02G079300;r=5B:96755686-96758071;t=TraesCS5B02G079300.1;tl=AR78Pl1sbj1Aaksc-15036-7492699) | nucleic acid binding  ATP binding | - |
| 31 | M36714 | TGCAGCTCCGCGTCAGTGGTGTCGCGGGTGAGGCTCTTCTGCTCGTCGGCGCGGATCGGAACTT_10 | Spike length | 5B | [287,752,969-287,780,293](http://ensembl.gramene.org/Triticum_aestivum/Location/View?db=core;g=TraesCS5B02G155600;r=5B:287752969-287780293;t=TraesCS5B02G155600.1;tl=AR78Pl1sbj1Aaksc-15039-7610389) | ATP binding  ATPase activity | - |
| 32 | M36808 | TGCAGCTCCGTGTCAGTGGTGTCGCGGGTGAGGCTCTTCTGCTCATCGGCGCGGATCGGAACTT_44 | Spike length | 5B | [287,752,969-287,780,293](http://ensembl.gramene.org/Triticum_aestivum/Location/View?db=core;g=TraesCS5B02G155600;r=5B:287752969-287780293;t=TraesCS5B02G155600.1;tl=AR78Pl1sbj1Aaksc-15040-7618580) | ATP binding  ATPase activity | - |

Table S8

Continued.

| 33 | M61151 | TGCAGTGCTTTCACACGATGCCCTTCAGAAAAGGAACACAGAGGAAAACAGAGTAGCTCAAGAA_28 | Spike length | 5B | [94,120,726-94,126,261](http://ensembl.gramene.org/Triticum_aestivum/Location/View?db=core;g=TraesCS5B02G078300;r=5B:94120726-94126261;t=TraesCS5B02G078300.1;tl=AR78Pl1sbj1Aaksc-15041-7627346) | catalytic activity  6-phosphofructokinase activity  ATP binding  kinase activity  transferase activity  metal ion binding  diphosphate-fructose-6-phosphate 1-phosphotransferase activity | glycolytic process  metabolic process  phosphorylation  carbohydrate phosphorylation  glycolytic process through fructose-6-phosphate |
| --- | --- | --- | --- | --- | --- | --- | --- |
| 34 | M2927 | TGCAGAATTCAATAATTAATCAACGACTTAGAAAGAATGAAAGGGCAAAAATTTGTGGAAATTG_20 | Spike length | 5B | [584,085,197-584,096,517](http://ensembl.gramene.org/Triticum_aestivum/Location/View?db=core;g=TraesCS5B02G408500;r=5B:584085197-584096517;t=TraesCS5B02G408500.1;tl=AR78Pl1sbj1Aaksc-15047-7629377) | drug transmembrane transporter activity  antiporter activity | drug transmembrane tnsport  transmembrane transport |
| 35 | M60899 | TGCAGTGCGCACCATTTCTGTGTTTGTGTTAATCGATCGATCTTGTTGAGCTATAGCAGGGCAT_52 | Leaf greenness | 5B | [684,227,667-684,231,381](http://ensembl.gramene.org/Triticum_aestivum/Location/View?db=core;g=TraesCS5B02G522900;r=5B:684227667-684231381;t=TraesCS5B02G522900.1;tl=yLpcrpWqJbYDirFr-15227-7664038) | drug transmembrane transporter activity | drug transmembrane transport  transmembrane transport |
| 36 | M39294 | TGCAGCTGCTGGGCGCAGGCCTCGCAGTGCATGTTGACGAGCAGCTCCACGGTGCGGGCCTCGA_63 | Spike length | 5B | [712,436,278-712,438,265](http://ensembl.gramene.org/Triticum_aestivum/Location/View?db=core;g=TraesCS5B02G571000;r=5B:712436278-712438265;tl=yLpcrpWqJbYDirFr-15230-7664052) | metal ion binding | metal ion transport |

Table S8

Continued.

| 37 | M41278 | TGCAGCTTGATCTCGTGAACGAGGCAGAGATCGGCCTGGAGGCGATATATGAGGCACTCGGGCA_59 | Spike length | 5B | [712,849,467-712,851,947](http://ensembl.gramene.org/Triticum_aestivum/Location/View?db=core;g=TraesCS5B02G571500;r=5B:712849467-712851947;t=TraesCS5B02G571500.1;tl=yLpcrpWqJbYDirFr-15231-7664067) | protein binding | - |
| --- | --- | --- | --- | --- | --- | --- | --- |
| 38 | M41279 | TGCAGCTTGATCTCGTGAATGAGGCAGAGATCGGCCTGGAGGCGATATATGAGGCACTCAGGCA_59 | Spike length | 5B | [712,849,467-712,851,947](http://ensembl.gramene.org/Triticum_aestivum/Location/View?db=core;g=TraesCS5B02G571500;r=5B:712849467-712851947;t=TraesCS5B02G571500.1;tl=yLpcrpWqJbYDirFr-15232-7664086) | protein binding | - |
| 39 | M50893 | TGCAGGGGACCGCACCTTTTGAGTCCTGTACGGGAGAAGGGTGATAAGGTTTTTGGGGAGTGCT_10 | Spike length | 5B | [308,548,305-308,552,970](http://ensembl.gramene.org/Triticum_aestivum/Location/View?db=core;g=TraesCS5B02G166300;r=5B:308548305-308552970;t=TraesCS5B02G166300.1;tl=yLpcrpWqJbYDirFr-15233-7664726) | catalytic activity  indole-3-glycerol-phosphate synthase activity | tryptophan metabolic process |
| 40 | M46473 | TGCAGGCAGTTCATGTACTCGTCACCGCCCTCCGACACTCGAAGCTCCCCGTGTTGGAGCTCGA_23 | Spike length | 5D | [481,183,554-481,185,531](http://ensembl.gramene.org/Triticum_aestivum/Location/View?db=core;g=TraesCS5D02G420800;r=5D:481183554-481185531;t=TraesCS5D02G420800.1;tl=yLpcrpWqJbYDirFr-15236-7676252) | DNA binding | regulation of transcription, DNA-templated |

Table S9

Description of expected MTAs using imputed SNPs for agronomic traits of Iranian wheat accessions exposed to the rain-fed condition

| Row | Marker | sequence | Trait | Chromosome | Position  (bp) | Molecular process | Biological process |
| --- | --- | --- | --- | --- | --- | --- | --- |
| 1 | M50966 | TGCAGGGGAGGGGCGAGGAAAAGCCTAGCCGCCGAAGCCGTAGAGGGTGCGGCCCTGGCGCTTG_20 | Grain yield | 1A | [485,012,383-485,012,973](http://ensembl.gramene.org/Triticum_aestivum/Location/View?db=core;g=TraesCS1A02G287800;r=1A:485012383-485012973;t=TraesCS1A02G287800.1;tl=QdmZhzXKlNvYQsSJ-19621-9277714) | DNA binding  (Histone H4)  protein heterodimerization activity  (Histone-fold) | nucleosome assembly  response to water deprivation |
| 2 | M13662 | TGCAGCACAGCAAGCAGCGGCTCAAGCCAAAGGAGTCGCAGGCATCGGGTCCTAGCCTCACCGA_59 | Thousand kernel weight | 1A | [591,147,143-591,152,457](http://ensembl.gramene.org/Triticum_aestivum/Location/View?db=core;g=TraesCS1A02G442500;r=1A:591147143-591152457;t=TraesCS1A02G442500.1;tl=QdmZhzXKlNvYQsSJ-19631-9277989) | metalloendopeptidase activity  (Peptidase M41-like)  ATP binding  (ATPase, AAA-type, core) | proteolysis |
| 3 | M27201 | TGCAGCCTGAGGGTGGAGGCGACGACGATGGCGAGGATGGCGAAGCAGGCCGAGATCGGAAGAG_25 | Grain filling | 4A | [13,745,915-13,749,251](http://ensembl.gramene.org/Triticum_aestivum/Location/View?db=core;g=TraesCS4A02G020100;r=4A:13745915-13749251;t=TraesCS4A02G020100.1;tl=QdmZhzXKlNvYQsSJ-19632-9278063) | symporter activity  (Lactose permease-like) | carbohydrate transport |
| 4 | M29508 | TGCAGCGCACCGCCACTACGGCGGAAGAGGTTGCGGCCGTAGCTGCCGCGGGCGCGTTTGGGTT_18 | Spike weight | 4A | [629,034,391-629,106,268](http://ensembl.gramene.org/Triticum_aestivum/Location/View?db=core;g=TraesCS4A02G354700;r=4A:629034391-629106268;t=TraesCS4A02G354700.1;tl=QdmZhzXKlNvYQsSJ-19636-9286259) | ATP binding  ATPase activity  (ABC transporter-like) | - |
| 5 | M38631 | TGCAGCTGCACCCGCTTGTTCTTGCCGATCACCCTGCTAGACGCCGCCGAGATCGGAAGAGCGG_44 | Days to heading and anthsis | 4A | [444,156,934-444,157,660](http://ensembl.gramene.org/Triticum_aestivum/Location/View?db=core;g=TraesCS4A02G174100;r=4A:444156934-444157660;t=TraesCS4A02G174100.1;tl=QdmZhzXKlNvYQsSJ-19638-9307123) | nucleic acid binding  (Zinc finger C2H2-type) | - |
| 6 | M64750 | TGCAGTTTATGTACGAACTTTGAGAATTCTCATCAGTGGCCAAACGCCCAAACTAACAATTGAA_34 | Canopy temperature | 4A | [630,897,051-630,899,273](http://ensembl.gramene.org/Triticum_aestivum/Location/View?db=core;g=TraesCS4A02G357600;r=4A:630897051-630899273;t=TraesCS4A02G357600.1;tl=v8mizTk4aZtxRyOm-20519-9679357) | DNA binding  (B3 DNA binding domain) | transcription, DNA-templated  regulation of transcription, DNA-templated |

Table S9

Continued.

| 7 | M56381 | TGCAGTACCTTTATTTGGAAGGATATGTAAAAGTTGGTTGGGTGGTACGTACTCCCTCCGTCCG_61 | Spike length | 4A | [501,125,479-501,129,913](http://ensembl.gramene.org/Triticum_aestivum/Location/View?db=core;g=TraesCS4A02G207900;r=4A:501125479-501129913;t=TraesCS4A02G207900.1;tl=v8mizTk4aZtxRyOm-20527-9763127) | nucleic acid binding  (DNA/RNA-binding protein Alba-like, Alba-like domain superfamily) |  |
| --- | --- | --- | --- | --- | --- | --- | --- |
| 8 | M38858 | TGCAGCTGCCGCTCCTCCAAGTTGCCCTTCGCTGCGCAAAGGACCTCGATCTCATCCGTTGCAT_5 | Peduncle length | 4A | [235,648,062-235,662,721](http://ensembl.gramene.org/Triticum_aestivum/Location/View?db=core;g=TraesCS4A02G143200;r=4A:235648062-235662721;tl=v8mizTk4aZtxRyOm-20529-9765456) | -  (Retinoblastoma-related protein) | G1/S transition of mitotic cell cycle  transcription, DNA-templated  regulation of transcription by RNA polymerase II  regulation of cell cycle |
| 9 | M25815 | TGCAGCCGTACGCGCAGTCCACCATCACGAACCGCCCGTGCCGCAAGCTCTCCTACAAGTTCTT_26 | Days to emergence | 4A | [379,016,349-379,045,201](http://ensembl.gramene.org/Triticum_aestivum/Location/View?db=core;g=TraesCS4A02G164300;r=4A:379016349-379045201;t=TraesCS4A02G164300.1;tl=v8mizTk4aZtxRyOm-20532-9767143) | arabinosyltransferase activity | protein glycosylation  cell wall pectin biosynthetic process  root hair cell development |
| 10 | M19485 | TGCAGCATCTCGCTGCTCCACGGGGACCCGTCGGCATCAGCGCCGAGATCGGAAGAGCGGGATC_20 | Days to heading and anthesis | 4A | [739,151,566-739,154,805](http://ensembl.gramene.org/Triticum_aestivum/Location/View?db=core;g=TraesCS4A02G485700;r=4A:739151566-739154805;tl=v8mizTk4aZtxRyOm-20537-9768496) | hydrolase activity, hydrolyzing O-glycosyl compounds  hydrolase activity  hydrolase activity, acting on glycosyl bonds  (Glycoside hydrolase, family 32) | carbohydrate metabolic process  metabolic process |

Table S9

Continued.

| 11 | M11116 | TGCAGCAAATTAATCTAGCTTTTAGTTTCCTTCAGGTATTTTGGATATGCCAGCAAATCGAAAG_29 | Peduncle length | 4A | [739,791,132-739,802,785](http://ensembl.gramene.org/Triticum_aestivum/Location/View?db=core;g=TraesCS4A02G487200;r=4A:739791132-739802785;t=TraesCS4A02G487200.1;tl=v8mizTk4aZtxRyOm-20539-9768740) | ADP binding  (NB-ARC) | - |
| --- | --- | --- | --- | --- | --- | --- | --- |
| 12 | M5972 | TGCAGAGCCAGGAGACGCAGGAGAACCCCTACGCGTACCAGGACGAGACCACCAAGAAGAGGCC_23 | Peduncle length | 4B | [1,696,842-1,698,167](http://ensembl.gramene.org/Triticum_aestivum/Location/View?db=core;g=TraesCS4B02G002800;r=4B:1696842-1698167;t=TraesCS4B02G002800.1;tl=v8mizTk4aZtxRyOm-20540-9768762) | DNA binding  protein heterodimerization activity  (Histone H2B, Histone H2A/H2B/H3, AND Histone-fold) | - |
| 13 | M36083 | TGCAGCTCATCTCCTAGCTAGCTGCTCCATCAGTCCTTCCTTCCTCGTCGCTCCAATGGCGCTC_31 | Grain filling period | 4B | [20,583,065-20,588,655](http://ensembl.gramene.org/Triticum_aestivum/Location/View?db=core;g=TraesCS4B02G027100;r=4B:20583065-20588655;t=TraesCS4B02G027100.1;tl=v8mizTk4aZtxRyOm-20541-9769084) | lipase activity  hydrolase activity, acting on ester bonds  (Lipase, GDSL, active site) | lipid metabolic process |
| 14 | M30688 | TGCAGCGCGCGCTCCAGGTTGCCGTCCCTCACCTGCACCACGATCCCCCGCGCCGCCTGCGTCT_56 | Thousand kernel weight | 4B | [24,855,498-24,858,072](http://ensembl.gramene.org/Triticum_aestivum/Location/View?db=core;g=TraesCS4B02G033900;r=4B:24855498-24858072;t=TraesCS4B02G033900.1;tl=v8mizTk4aZtxRyOm-20542-9769316) | structural constituent of ribosome  (Ribosomal protein S21) | translation |
| 15 | M736 | TGCAGAAAGGTACCACTCATTCGTACATCACTCCAACTGATGTATGAAGGTTGTTCATGGCGAC_18 | Straw weight | 4B | [481,233,765-481,237,258](http://ensembl.gramene.org/Triticum_aestivum/Location/View?db=core;g=TraesCS4B02G230100;r=4B:481233765-481237258;t=TraesCS4B02G230100.1;tl=pPIrprHtr8pUgaGc-20702-9867240) | hydrolase activity | phosphatidylinositol dephosphorylation |
| 16 | M55557 | TGCAGGTTTTGCCTAAGAAAAACTCAGAATTCACTCAAAAAAATCAGATTGCTGTAAACTGCAC_15 | Canopy temperature | 4B | [613,031,990-613,041,407](http://ensembl.gramene.org/Triticum_aestivum/Location/View?db=core;g=TraesCS4B02G322300;r=4B:613031990-613041407;t=TraesCS4B02G322300.1;tl=pPIrprHtr8pUgaGc-20706-9867315) | drug transmembrane transporter activity  antiporter activity  (Multi antimicrobial extrusion protein) | drug transmembrane transport  transmembrane transport |
| 17 | M55558 | TGCAGGTTTTGCCTAAGAAAAACTCAGAATTCACTGGAAAAAAATCAGATTGCTGTAAACTGCA_15 | Canopy temperature | [613,031,990-613,041,407](http://ensembl.gramene.org/Triticum_aestivum/Location/View?db=core;g=TraesCS4B02G322300;r=4B:613031990-613041407;t=TraesCS4B02G322300.1;tl=pPIrprHtr8pUgaGc-20707-9873452) |  | drug transmembrane transporter activity  antiporter activity  (Multi antimicrobial extrusion protein) | drug transmembrane transport  transmembrane transport |

Table S9

Continued.

| 18 | M50187 | TGCAGGGCAGTCGAAGCAGTTGCTGGGTCAGAGGCGTGGAGTTGCACTGGAGCAACAGGAGTCG_54 | Spike length | 4B | [222,603,782-222,615,097](http://ensembl.gramene.org/Triticum_aestivum/Location/View?db=core;g=TraesCS4B02G149600;r=4B:222603782-222615097;t=TraesCS4B02G149600.1;tl=Rdx9g7l7vxfreuNz-20754-9899406) | transmembrane transporter activity  (Major facilitator, sugar transporter-like) | transmembrane transport |
| --- | --- | --- | --- | --- | --- | --- | --- |
| 19 | M10940 | TGCAGCAAAGGAAAAGAAGAAACATGGCCGCCCGCGCCAACCCGCCGCTTCGTTTCCTCCCTCC_42 | Seed number | 5A | [26,153,880-26,157,442](http://ensembl.gramene.org/Triticum_aestivum/Location/View?db=core;g=TraesCS5A02G030200;r=5A:26153880-26157442;t=TraesCS5A02G030200.1;tl=5fcuDdSc5DnOR8Ld-20764-9912380) | GTP binding | ribosome biogenesis |
| 20 | M52925 | TGCAGGTCATTTTCCAGTAAATTGAGATAAACGATAAGGCTCCGAGATCGGAAGAGCGGGATCA_23 | Seed number | 5A | [675,957,554-675,960,737](http://ensembl.gramene.org/Triticum_aestivum/Location/View?db=core;g=TraesCS5A02G512100;r=5A:675957554-675960737;t=TraesCS5A02G512100.1;tl=5fcuDdSc5DnOR8Ld-20771-9912625) | structural constituent of ribosome  (Ribosomal protein L11/L12) | translation |
| 21 | M41689 | TGCAGCTTGTCGGTCCTCTCCGACATGGCGTCGAGCACCCGCCGAGTCTGGGCCGAGGGTTTGG_15 | Leaf greenness | 5B | [334,871,156-334,874,981](http://ensembl.gramene.org/Triticum_aestivum/Location/View?db=core;g=TraesCS5B02G184100;r=5B:334871156-334874981;t=TraesCS5B02G184100.1;tl=f6LT8sdyxOBo3vIH-22458-10686669) | catalytic activity  ATP binding  zinc ion binding  pyridoxal phosphate binding  cysteine desulfurase activity  (Cysteine desulfurase IscS) | iron-sulfur cluster assembly  [2Fe-2S] cluster assembly |
| 22 | M13458 | TGCAGCACACACAAAATGGGCATCCACGCCGATGTGCTTGGTGAGCTCATGCTTCACCGAGATC_8 | Spike length | 5B | [105,687,602-105,701,843](http://ensembl.gramene.org/Triticum_aestivum/Location/View?db=core;g=TraesCS5B02G083500;r=5B:105687602-105701843;t=TraesCS5B02G083500.1;tl=f6LT8sdyxOBo3vIH-22463-10687109) | metalloendopeptidase activity  transferase activity  transferase activity, transferring acyl groups  transferase activity, transferring acyl groups other than amino-acyl groups  metal ion binding  N(6)-L-threonylcarbamoyladenine synthase activity  (tRNA N6-adenosine threonylcarbamoyltransferase, TsaD) | tRNA threonylcarbamoyladenosine modification  proteolysis  tRNA processing |

Table S9

Continued.

| 23 | M36886 | TGCAGCTCCTCCAGCTCCGCATCGGCCTCCGCCTCTAACTCGATGAAATGCCGAGATCGGAAGA_20 | Plant height | 5B | [96,755,686-96,758,071](http://ensembl.gramene.org/Triticum_aestivum/Location/View?db=core;g=TraesCS5B02G079300;r=5B:96755686-96758071;t=TraesCS5B02G079300.1;tl=f6LT8sdyxOBo3vIH-22465-10688546) | nucleic acid binding  ATP binding  (DEAD/DEAH box helicase domain) | - |
| --- | --- | --- | --- | --- | --- | --- | --- |
| 24 | M59282 | TGCAGTCGTGGATAATGCACCTTGCGGTGTCAGGGGGTGACGTCAGCGATGAGTCCACCG_39 | Days to heading | 5B | [11,550,484-11,556,238](http://ensembl.gramene.org/Triticum_aestivum/Location/View?db=core;g=TraesCS5B02G011800;r=5B:11550484-11556238;t=TraesCS5B02G011800.1;tl=f6LT8sdyxOBo3vIH-22471-10705912) | catalytic activity  hydrolase activity, hydrolyzing O-glycosyl compounds  alpha-galactosidase activity  hydrolase activity  hydrolase activity, acting on glycosyl bonds  raffinose alpha-galactosidase activity  (Glycoside hydrolase, family 27) | carbohydrate metabolic process  metabolic process |
| 25 | M36808 | TGCAGCTCCGTGTCAGTGGTGTCGCGGGTGAGGCTCTTCTGCTCATCGGCGCGGATCGGAACTT_44 | Days to heading | 5B | [287,752,969-287,780,293](http://ensembl.gramene.org/Triticum_aestivum/Location/View?db=core;g=TraesCS5B02G155600;r=5B:287752969-287780293;t=TraesCS5B02G155600.1;tl=f6LT8sdyxOBo3vIH-22473-10715120) | ATP binding  ATPase activity  (ABC transporter-like) | - |
| 26 | M44154 | TGCAGGAGCACCAGCGCGGCAGCGGTGGCGACGACGGGGCTACCAGCTGCCCGCCGAGATCGGA_20 | Spike length | 5B | [363,662,473-363,670,095](http://ensembl.gramene.org/Triticum_aestivum/Location/View?db=core;g=TraesCS5B02G201100;r=5B:363662473-363670095;t=TraesCS5B02G201100.1;tl=f6LT8sdyxOBo3vIH-22477-10737740) | catalytic activity  hydrolase activity, hydrolyzing O-glycosyl compounds  cellulase activity  hydrolase activity  hydrolase activity, acting on glycosyl bonds | polysaccharide catabolic process  carbohydrate metabolic process  metabolic process  cellulose catabolic process |

Table S9

Continued.

| 27 | M17806 | TGCAGCAGGCAAGGTATCTCCAGGCGAACTATATCATCGCAATATACGAGCTTCAGGTGCTCCA_61 | Days to heading, anthsis and physiological maturity | 5B | [457,966,329-457,970,659](http://ensembl.gramene.org/Triticum_aestivum/Location/View?db=core;g=TraesCS5B02G272500;r=5B:457966329-457970659;t=TraesCS5B02G272500.1;tl=uaumsxiVjbi9jC3v-22709-10925784) | protein binding  (F-box-like domain superfamily) | - |
| --- | --- | --- | --- | --- | --- | --- | --- |
| 28 | M48027 | TGCAGGCGCCCGTTTGCAAATTGCACTGTAACGGGCGCCTGCGGCGCTAAATAGGAAAACCCCC_32 | Straw weight | 5B | [382,891,251-382,898,903](http://ensembl.gramene.org/Triticum_aestivum/Location/View?db=core;g=TraesCS5B02G212200;r=5B:382891251-382898903;t=TraesCS5B02G212200.1;tl=uaumsxiVjbi9jC3v-22713-10928915) | electron transfer activity  iron-sulfur cluster binding  2 iron, 2 sulfur cluster binding  (2Fe-2S ferredoxin-type iron-sulfur binding domain, Adrenodoxin, iron-sulphur binding site) | electron transport chain |
| 29 | M25701 | TGCAGCCGCTCTTCGGCGGCTCTTGCATCGATGAGGTCGCGGGTGGGGCTGATGCGGAAAAGTG_19 | Peduncle length | 5B | [513,711,454-513,713,217](http://ensembl.gramene.org/Triticum_aestivum/Location/View?db=core;g=TraesCS5B02G329700;r=5B:513711454-513713217;t=TraesCS5B02G329700.1;tl=uaumsxiVjbi9jC3v-22720-10930061) | D-arabinono-1,4-lactone oxidase activity  oxidoreductase activity  flavin adenine dinucleotide binding  FAD binding | oxidation-reduction process |
| 30 | M25700 | TGCAGCCGCTCTTCGGCGGCTCTTGCATCGATGAGCTCGCGGGTGCGGGTAAGGGGCAAGTCGT_35 | Peduncle length | 5B | [513,646,921-513,649,139](http://ensembl.gramene.org/Triticum_aestivum/Location/View?db=core;g=TraesCS5B02G329600;r=5B:513646921-513649139;t=TraesCS5B02G329600.1;tl=uaumsxiVjbi9jC3v-22721-10930090) | catalytic activity  D-arabinono-1,4-lactone oxidase activity  oxidoreductase activity  flavin adenine dinucleotide binding  FAD binding | oxidation-reduction process |

Table S9

Continued.

| 31 | M32200 | TGCAGCGGCGGCGGAGGGATCGGCGGTCGGCGTAGCGCCTGACGCTCCTATGGCCTTGGGGCCG_49 | Straw weight | 5B | [287,752,969-287,780,293](http://ensembl.gramene.org/Triticum_aestivum/Location/View?db=core;g=TraesCS5B02G155600;r=5B:287752969-287780293;t=TraesCS5B02G155600.1;tl=uaumsxiVjbi9jC3v-22731-10937649) | ATP binding  ATPase activity  (ABC transporter-like) | - |
| --- | --- | --- | --- | --- | --- | --- | --- |
| 32 | M1727 | TGCAGAAGAAGGCTGGCGGAGGCGGTAGTCGAGCTGGCGCTGGTGACCAATGGCGATGCCCTGG_26 | Grain filling | 5D | [505,822,665-505,824,306](http://ensembl.gramene.org/Triticum_aestivum/Location/View?db=core;g=TraesCS5D02G462000;r=5D:505822665-505824306;t=TraesCS5D02G462000.1;tl=uaumsxiVjbi9jC3v-22738-10946546) | nucleic acid binding  (Zinc finger C2H2-type) | - |
| 33 | M2717 | TGCAGAATCTCATGTTGAAAAACCTGAGCTTATCGCAAGCCCTAATGAGGTCGGTGATGTCGGA_51 | Seed number | 5D | [542,408,938-542,411,564](http://ensembl.gramene.org/Triticum_aestivum/Location/View?db=core;g=TraesCS5D02G521400;r=5D:542408938-542411564;t=TraesCS5D02G521400.1;tl=8fbN3OUTO1lCInrR-23601-11166966) | protein binding  (F-box domain) | - |
| 34 | M27533 | TGCAGCCTTACTTGCAGTCAGTCGCAGATGGAGGGGACTGACTATGTGTGCTGGTTTCTTTGTT_57 | Seed number | 5D | [549,631,107-549,635,538](http://ensembl.gramene.org/Triticum_aestivum/Location/View?db=core;g=TraesCS5D02G536100;r=5D:549631107-549635538;t=TraesCS5D02G536100.1;tl=8fbN3OUTO1lCInrR-23602-11167035) | transmembrane transporter activity  (Mitochondrial carrier domain protein) | transmembrane transport |
| 35 | M31854 | TGCAGCGGCCCAGCGAACGCCGCGGAGGCCGCCGAGTGCGGCGAGTACCACAGCAGGCCTGGCG_13 | Seed number | 5D | [29,608,990-29,619,747](http://ensembl.gramene.org/Triticum_aestivum/Location/View?db=core;g=TraesCS5D02G032300;r=5D:29608990-29619747;t=TraesCS5D02G032300.1;tl=8fbN3OUTO1lCInrR-23605-11167256) | ADP binding  (NB-ARC) | - |
| 36 | M63997 | TGCAGTTGCACTAGTTTTTCCTGGCTTGCTTGCTTGCTTCTTAGATGTACGTACGTACGTACGT_61 | Seed number | 5D | [563,599,879-563,600,451](http://ensembl.gramene.org/Triticum_aestivum/Location/View?db=core;g=TraesCS5D02G564600;r=5D:563599879-563600451;t=TraesCS5D02G564600.1;tl=8fbN3OUTO1lCInrR-23608-11167737) | DNA binding  (Homeobox-like domain superfamily) | - |
